# Chromosome-level genome assembly of a parent species of widely cultivated azaleas

**DOI:** 10.1038/s41467-020-18771-4

**Published:** 2020-10-19

**Authors:** Fu-Sheng Yang, Shuai Nie, Hui Liu, Tian-Le Shi, Xue-Chan Tian, Shan-Shan Zhou, Yu-Tao Bao, Kai-Hua Jia, Jing-Fang Guo, Wei Zhao, Na An, Ren-Gang Zhang, Quan-Zheng Yun, Xin-Zhu Wang, Chanaka Mannapperuma, Ilga Porth, Yousry Aly El-Kassaby, Nathaniel Robert Street, Xiao-Ru Wang, Yves Van de Peer, Jian-Feng Mao

**Affiliations:** 1 State Key Laboratory of Systematic and Evolutionary Botany, Institute of Botany, Chinese Academy of Sciences, 100093 Beijing, China; 2 Beijing Advanced Innovation Center for Tree Breeding by Molecular Design, National Engineering Laboratory for Tree Breeding, College of Biological Sciences and Technology, Beijing Forestry University, 100083 Beijing, China; 3 Department of Ecology and Environmental Science, Umeå Plant Science Centre, Umeå University, SE-901 87 Umeå, Sweden; 4 University of Chinese Academy of Sciences, 100049 Beijing, China; 5 Beijing Ori-Gene Science and Technology Co. Ltd, 102206 Beijing, China; 6 Umeå Plant Science Centre, Department of Plant Physiology, Umeå University, SE-901 87 Umeå, Sweden; 7 Département des Sciences du Bois et de la Forêt, Faculté de Foresterie, de Géographie et Géomatique, Université Laval Québec, Québec, QC G1V 0A6 Canada; 8 Department of Forest and Conservation Sciences, Faculty of Forestry, University of British Columbia, Vancouver, BC V6T 1Z4 Canada; 9 Department of Plant Biotechnology and Bioinformatics and VIB Center for Plant Systems Biology, Ghent University, 9052 Ghent, Belgium; 10 Centre for Microbial Ecology and Genomics, Department of Biochemistry, Genetics and Microbiology Genetics, University of Pretoria, Private bag X20, Pretoria, 0028 South Africa; 11 College of Horticulture, Nanjing Agricultural University, 210095 Nanjing, China

**Keywords:** Agricultural genetics, Evolutionary biology, Comparative genomics, Plant breeding

## Abstract

Azaleas (Ericaceae) comprise one of the most diverse ornamental plants, renowned for their cultural and economic importance. We present a chromosome-scale genome assembly for *Rhododendron simsii*, the primary ancestor of azalea cultivars. Genome analyses unveil the remnants of an ancient whole-genome duplication preceding the radiation of most Ericaceae, likely contributing to the genomic architecture of flowering time. Small-scale gene duplications contribute to the expansion of gene families involved in azalea pigment biosynthesis. We reconstruct entire metabolic pathways for anthocyanins and carotenoids and their potential regulatory networks by detailed analysis of time-ordered gene co-expression networks. MYB, bHLH, and WD40 transcription factors may collectively regulate anthocyanin accumulation in *R. simsii*, particularly at the initial stages of flower coloration, and with WRKY transcription factors controlling progressive flower coloring at later stages. This work provides a cornerstone for understanding the underlying genetics governing flower timing and coloration and could accelerate selective breeding in azalea.

## Abstract (web summary)

Azaleas are one of the most diverse ornamental plants and have cultural and economic importance. Here, the authors report a chromosome-scale genome assembly for the primary ancestor of the azalea cultivar *Rhododendro simsi* and identify transcription factors that may function in flower coloration at different stages.

## Introduction

The genus *Rhododendron* (Ericaceae) harbors more than 1000 species and 30,000 cultivars and is known for the outstanding beauty and diversity of its corollas^1,2^. During the 18th century, several Chinese azalea cultivars were introduced to Europe (especially to England and Belgium), facilitating their breeding for ornamental use^3^. *R. simsii* (potted azalea) is an evergreen species of the subgenus *Tsutsusi*, endemic to East Asia, and is the most widely cultivated *Rhododendron* species with numerous cultivars selected from a diversity of wild relatives and natural hybrids^3^. At present, the annual production of *R. simsii* hybrids has reached approximately 40 million pots in Belgium alone^3,4^. Although *R. simsii* natural blooming extends from the end of spring to the beginning of summer, some ‘easy-care and color-rich’ cultivars can bloom as early as Christmas and Spring Festivals, when few other flowering plants are available^2,3^. Currently, azalea cultivars have become the focus of intensive ornamental use through hybridizations and production programs, attaining prominence as pot plants and landscape shrubs in Europe, North American, and Asia^3^.

Breeding and development of new ornamental cultivars is mainly focused on flower color. Moreover, flower color is also of paramount importance for plant ecology and evolution^5^. Since the work of Gregor Mendel (1856–1863), flower color has successfully contributed to the elucidation of the fundamental principles of genetics, while more recently, genomics has provided insights into the evolution of the biochemical pathways and regulatory networks underlying phenotypic traits, including flower color^5^. So far, pigment analyses for azalea flowers were based on pigment type and composition percentage; however, their underlying genetic and regulatory mechanisms are largely unknown. Recently, some of the flavonoid biosynthesis structural genes were isolated from several *Rhododendron* species and cultivars, and the spatiotemporal expression patterns of some key node-genes were analyzed^3,4,6^. Although two *Rhododendron* genomes were released recently, both were obtained by Illumina short read sequencing^7,8^. The lack of a high-quality *Rhododendron* species whole-genome sequence has seriously hampered the unraveling of their color formation, in spite of the known long-term breeding history^3,8^.

Here, we present a chromosome-scale genome assembly for *R. simsii*. The genome of *R. simsii* is determined by a combination of long-read sequencing and Hi-C scaffolding technologies. In total, a 529 Mb genome sequence is assembled, and >91% of the genome could be anchored on 13 chromosomes with a scaffold N50 of 36 Mb. We detect the remnants of a whole genome duplication and find tandem and other small-scale gene duplications to be the key drivers for gene family expansions. Furthermore, we unravel the metabolic co-expression network of flower pigmentation and identified the structural genes, and their potential regulators, of flower coloring through time-ordered comparative transcriptome analyses. The availability of this reference genome sequence and information on the molecular basis and the genetic mechanisms governing flower color in *Rhododendron* present valuable resources for the development of consumer-oriented selective breeding novelties of azalea.

## Results

### *R. simsii* genome assembly and annotation

The genome size of *R. simsii* (Supplementary Fig. 1) estimated with *K*-mer analysis to be ~525 Mb (Supplementary Fig. 2) was larger than its size estimation by flow cytometry for yet unresolved reasons (Supplementary Table 1). We produced 100× coverage of PacBio long-read sequencing data, 170× coverage of short reads of PCR-free Illumina sequencing, and 100× coverage of Hi-C paired-end reads (Table 1 and Supplementary Fig. 3). After primary assembly, comparison, correction, polishing, and scaffolding, a final assembly of 529 Mb was obtained. The assembly is slightly larger than the estimated genome size, which may be due to high heterozygosity (~1.78%, estimated with *K*-mer frequency, see Supplementary Note 1 for details). After mapping the Illumina reads to the final assembly, single nucleotide polymorphisms (SNPs) were identified with SAMtools^9^ (with default settings) and obtained a SNP heterozygosity level of ~1.07% and a single base error rate of ~0.0054% was obtained. There was no obvious GC bias in the sequencing data from PacBio single-molecule real-time (SMRT) technology; however, a GC bias was detected for the Illumina sequencing data **(**Supplementary Fig. 4**)**, confirming the advantage of PacBio SMRT technology over Illumina for genome sequencing^10^, in addition to the longer read length provided.

**Table 1:** The statistics for genome sequencing of *Rhododendron*.

	*R. simsii*	*R. delavayi*	*R. williamsianum*
Sequencing			
Raw bases of WGS-PacBio Sequel (Gb)	51.15	*	*
Raw bases of WGS-Illumina (Gb)	91.49	336.83	*
Raw bases of Hi-C (Gb)	55.681	*	*
Raw bases of mRNAseq (Gb)	422.149	*	*
Assembly			
Genome size (Mb)	528.6	695.1	532.1
Number of scaffolds	552	193,091	11,985
N50 of scaffolds (bp)	36,350,743	637,826	218,828
L50 of scaffolds	7	*	*
Chromosome-scale scaffolds (bp)	481,946,564 (91.17%)	0	368,385,547 (69%)
Number of contigs	911	*	*
N50 of contigs (bp)	2,234,511	61,801	*
L50 of contigs	66	*	*
Number of Gap	359	*	*
Complete BUSCOs	93.68%	92.80%	89%
GC content of the genome (%)	38.91%	*	*
Annotation			
Number of predicted genes	34,170	*	*
Number of predicted protein-coding genes	32,999	32,938	23,559
Average gene length (bp)	5,089.22	4,434.22	4,628
Average CDS length (bp)	1,288.73	1,153.21	*
Average exon per transcript	5	4.62	5.68
Number of tRNAs	482	*	*
Number of rRNAs	64	*	*
Repeat sequences (bp)	250,988,768 (47.48%)	359,874,503 (51.77%)	*
Annotated to Swiss-Prot	19,079 (57.80%)	22,693 (68.90%)	*
Annotated to PFAM	24,301 (73.60%)	*	*
Annotated to GO	25,038 (75.90%)	16,471 (50.00%)	18,538 (79%)
Annotated to KO	11,506 (34.90%)	*	*

The assembly for *R. simsii* was compared with two previously reported genome assemblies for *Rhododendron* species, *R. williamsianum*, and *R. delavayi*. Asterisk (*) indicates data were not shown in the original articles.

The final assembly consisted of 911 contigs and 552 scaffolds (13 chromosome-level scaffolds, one chloroplast genome, one mitochondrial genome, and 537 super contigs) with contig N50 of 2.2 Mb and scaffold N50 of 36 Mb (Table 1, Supplementary Tables 2, 3 and Supplementary Fig. 5). The number of chromosome-scale super scaffolds is consistent with the species’ determined chromosome number of 13^11^. The high fidelity of the assembly was supported by the high 10-fold minimum genome coverage of 99.3% (Illumina) and 98.6% (PacBio), and the high mapping rates of 93.3% (Illumina) and 90.9% (PacBio). The high completeness of this assembly was also evidenced by a 93.7% (1349 genes) Benchmarking Universal Single Copy Orthologs (BUSCO) recovery score^12^ (Table 1 and Supplementary Table 4), which is better compared to two recently released *Rhododendron* genomes^7,8^ obtained by Illumina short read sequencing (Supplementary Fig. 6). Judged by the high long terminal repeat (LTR) Assembly Index (LAI)^13^ score of 18.10, the *R. simsii* genome attained reference level quality.

We predicted 34,170 genes, and the average lengths for total gene regions, transcript, coding sequence (CDS), exon sequence, and intron sequence are 5089.2, 1416.3, 1288.7, 259.7, and 403.1 bp, respectively (Table 1 and Supplementary Table 5). A total of 32,999 protein-coding genes were predicted, which is considerably more than those annotated for the *R. williamsianum* genome (23,559 genes)^7^, but similar to those for the *R. delavayi* genome (32,938)^8^ (Table 1). In addition, we predicted 482 tRNAs, 64 rRNAs including eight 28S, six 18S and 50 5S rRNAs, and 625 other non-coding RNAs (211 miRNAs, 16 tRNAs and 158 snoRNAs) (Table 1 and Supplementary Table 6).

Among the predicted protein-coding genes, 96.44% could be annotated through at least one of the following protein-related databases: the NCBI non-redundant protein database (NR) (85.70%), the Swiss-Prot protein database (57.80%), the Translated European Molecular Biology Laboratory (TrEMBL) database (84.90%), the protein families database (Pfam) (73.60%), and the Gene Ontology (GO) database (57.54%) (Table 1 and Supplementary Table 7).

### *R. simsii* is an ancient polyploid

**Fig. 1:**
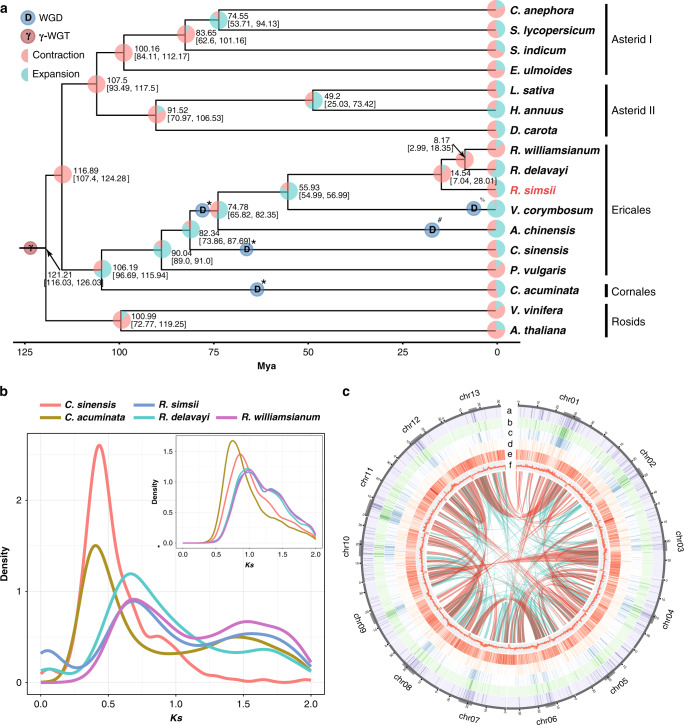
Genome evolutionary history. **a** Chronogram showing divergence times and genome duplications in asterids (asterid II, asterid I, Ericales, and Cornales), with node age and the 95% confidence intervals labeled. Resolved polyploidization events are shown with blue (duplications) and red translucent dots (triplications). Pie charts show the proportions of gene families that underwent expansion or contraction. Predicated WGD only shown for Ericales and Cornales. *WGD identified in this study. ^#^WGD reported in Wang et al.^69^. ^%^WGD reported in Colle et al.^18^. **b** *K*s distribution on the upper right (insert) is showing *K*s distribution from orthologs between *Vitis vinifera* and each of the five species (*Rhododendron delavayi*, *R. simsii*, *R. williamsianum*, *Camellia sinensis*, and *Camptotheca acuminata*). *K*s distribution in the lower left showing *K*s distribution from paralogs within *Rhododendron delavayi*, *R. simsii*, *R. williamsianum*, *Camellia sinensis*, and *Camptotheca acuminata*. **c** Synteny and distribution of genomic features. a, the density of Ogre, a family of *Gypsy* LTR-RT. b, the density of Tekay, a family of *Gypsy* LTR-RT. **c**, The density of all *Gypsy* LTR-RT. **d**, the density of all *Copia* LTR-RT. **e**, gene density. **f**, histogram of GC content. A map connecting homologous regions of the genome is shown. The red lines represent syntenic regions for the WGD event (78 Mya) and blue lines represent γ-WGD event. The line segments of bold gray in outer circles indicate predicated centromeres and pericentromeric regions on the 13 chromosomes.

A phylogenetic tree was constructed for the 15 species of asterids and two outgroup species, using 806 orthogroups (see “Methods” section). The phylogenetic relationship between and within the main clades (Asterid I, Asterid II, and Ericales) agree with previous studies^14–17^. Molecular dating suggests that *R. simsii* diverged from the most recent common ancestor of *R. delavayi* and *R. williamsianum* around 14.54 Mya, following the divergence of *Rhododendron* and *Vaccinium corymbosum*^18^ around 55.93 Mya (Fig. 1a).

Synonymous substitutions per site (*K*s) age distributions and collinearity/synteny analyses unveiled evidence for an ancient whole-genome duplication (WGD) event in the lineage leading to *Rhododendron*, around 78 Mya (Fig. 1a) (see “Methods” section for details), with three *Rhododendron* species (*R. simsii*, *R. delavayi*, and *R. williamsianum*) showing signature *K*s peaks at about 0.65 (Fig. 1b lower left). Moreover, intra-genomic analysis showed that 18.83% of the genome lies within duplicated segments (6,213/32,999 collinear genes distributed along 289 duplicated blocks; Supplementary Table 8). We also built a gene homology dot plot from one randomly selected set of chromosomes from the tetraploid *V. corymbosum* and the 13 chromosome-level scaffolds of *R. simsii* (Supplementary Fig. 7). Homologous regions showed a 1:1 correspondence, suggesting that azalea did not share the recent WGD event reported for blueberry^18^. At the same time, we detected a 2:1 syntenic relationship between *R. simsii* and *Vitis vinifera*^14^, a 4:2 syntenic relationship between *Actinidia chinensis*^19^ and *R. simsii*, and a 4:1 syntenic relationship between *V. corymbosum* and *V. vinifera*, which provide additional evidence for a WGD event in the common ancestor of *Rhohodendron*, *Vaccinium*, and *Actinidia* (Fig. 1a and Supplementary Fig. 7). Apart from the WGDs described above, *Ks* distributions also provide evidence for WGDs in *Camellia sinensis*^20^ (*K*s peak at 0.45 and duplication time around 68 Mya) and *Camptotheca acuminata*^21^ (0.4 and 62 Mya, respectively) (Fig. 1b lower left).

We also calculated *K*s values of one-versus-one orthologs between *V. vinifera* and five asterids species (*R. simsii*, *R. delavayi*, *R. williamsianum, C. sinensis*, and *C. acuminata*) and calculated the number of substitutions per synonymous site per year (*r*) with the formula, following *r* = *K*s/(2 × (divergence time))^16^. From this relative rate test, we found that species from Ericales and Cornales have a similar substitution rate (Fig. 1b upper right).

### *Gypsy* dominated pericentromeric regions in *R. simsii*

We predicted 954,329 repeat elements, totaling 250,988,768 bp (47.48%) sequence of the assembled genome, containing predominantly known transposable elements (TEs) (25.56%), uncharacterized TEs (19.24%), and a smaller proportion of simple repeats (1.41%). Repeat annotations are provided in Supplementary Fig. 8 and Supplementary Table 9. The uncharacterized (unknown) TEs may contain highly degenerated TE copies or lack distinct protein-coding sequences required for further classification. More work is needed to elucidate the structural and/or sequence diversity of the uncharacterized TEs.

Long terminal repeat retrotransposons (LTR-RTs) represented the highest portion (17.01%) in the genome, with superfamilies of *Gypsy* (11.90% of the genome sequence) and *Copia* (4.00%) being dominant. However, we found that *R. simsii* features low LTR-RT accumulation (*S* + *T* + *I* = 14,577) but high removal rates (proportion of LTR clusters with *S*:*I* > 3), where *S* is solo-LTR, *T* is truncated LTR-RT and *I* is intact LTR-RT (Supplementary Fig. 9 and Supplementary Table 10), which may be one reason for the overall lower proportion of TE in *R. simsii* than in other species analyzed here.

A total of 825 intact *Gypsy* (8.68 Mb) and 1303 intact *Copia* (8.20 Mb) LTR-TRs were identified across the whole *R. simsii* genome, with recent bursts, as well as a single ancient amplification peak predicted at ~2 Mya for most clades of both *Gypsy* and *Copia* (Supplementary Fig. 9 and Supplementary Table 11). Overall, we found 10.5-84.1% among the different subgroups of *Gypsy* and *Copia* preferentially residing in gene regions, particularly the chloroplast RNA splicing and ribosome maturation (CRM) family of *Gypsy* (57.63%) and the Ale family of *Copia* (52.73%), Supplementary Figs. 10a, b and Supplementary Table 11. However, most members of *Gypsy* and *Copia* were found 3-5 Kb distant from genic regions (Supplementary Fig. 10c). Insertion dynamics were similar between LTR-TRs proximal to and far away from genes, and among subgroups of both *Gypsy* and *Copia* (Supplementary Figs. 10d, e and f).

Gene density decreased and GC content increased from the chromosome ends towards the centromeres (Fig. 1c and Supplementary Fig. 11). We found that the accumulated *Gypsy* elements tend to be clustered in the pericentromeric regions, particularly Tekay and Ogre, yet there was a relatively even distribution for *Copia* elements along the chromosomes. Moreover, it was found that more *Copia* elements experienced positive selection (*K*a/*K*s > 1) in their reverse transcriptase (RT) domains, while RT domains of *Gypsy* elements showed lower *K*a/*K*s ratios and relatively smaller *K*a values (Supplementary Fig. 12). The distinct behaviors of *Gypsy* and *Copia* remain to be elucidated.

### TD/PD contributed to gene family expansion in *R. simsii*

**Fig. 2:**
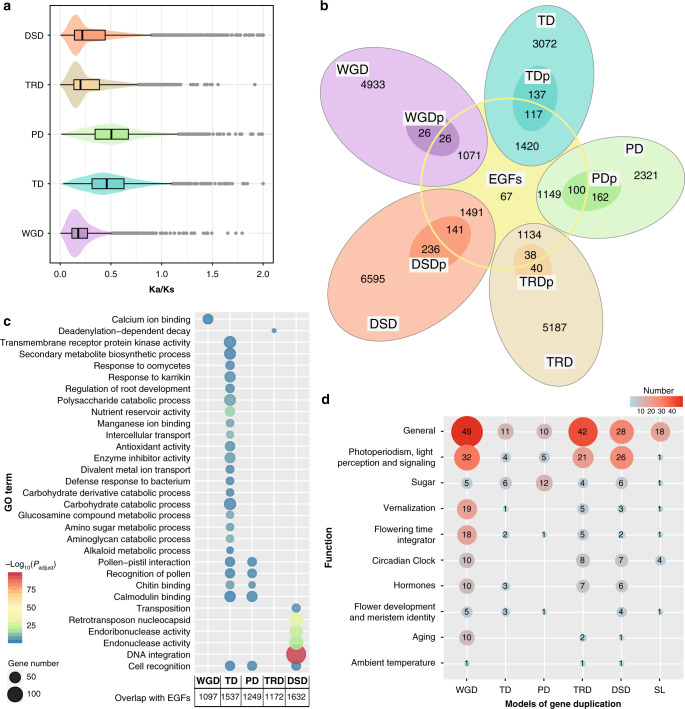
Gene duplication and evolution. **a** The *K*a/*K*s ratio distributions of gene pairs derived from different modes of duplication. WGD whole-genome duplication, TD tandem duplication, PD proximal duplication, TRD transposed duplication, DSD dispersed duplication. In the boxplot representations, points are outliers; the center line is the median; the lower and upper hinges correspond to the first and third quartiles (25th and 75th percentiles); whiskers extend to the minimum (left whiskers) and maximum (right whiskers) estimates located within the 1.5× interquartile range (IQR) of the first and third quartiles, respectively. Gaussian kernel estimates of *K*a/*K*s of different duplicated groups are shown as violins; 6056 WGD, 4746 TD, 3732 PD, 6399 TRD, and 8463 DSD duplicates were incorporated in the statistics. **b** Venn diagram shows the possible logical relations between members of expanded gene family and modes of duplication (WGD, TD, PD, TRD, and DSD). TDp: tandem duplication experienced positive selection, PDp: proximal duplication experienced positive selection, DSDp: dispersed duplication experienced positive selection, EGFs: expanded gene families. **c** Functional enrichment analysis of genes overlapping between expanded gene families (EGFs) and five modes of duplication (WGD, TD, PD, TRD, and DSD). The enriched GO terms with corrected *P* value <0.001 are presented. The color of circles represents the statistical significance of enriched GO terms. The size of the circles represents the number of genes in a GO term. For all annotated genes their GO terms are provided as background information. ‘*P* adjust’ is the Benjamini–Hochberg false discovery rate (FDR) adjusted *P* value. **d** The numbers of genes from different families and gene duplications are provided. SL singletons.

We identified 29,396 duplicated genes that were classified into five different categories: 6056 whole-genome duplicates (WGD duplicates, 18.4%), 4746 tandem duplicates (TD, 14.4%), 3732 proximal duplicates (PD, 11.3%), 6399 transposed duplicates (TRD, 19.4%), and 8463 dispersed duplicates (DSD, 25.6%) (see “Methods” section, Supplementary Fig. 13). We compared the *K*s and *K*a/*K*s distribution among different modes of gene duplication. Higher *K*a/*K*s ratios and smaller *K*s values were found for tandem and proximal duplicate gene pairs (Fig. 2a and Supplementary Fig. 14), suggesting an ongoing and continuous process for tandem and proximal duplications and more rapid sequence divergence and stronger positive selection than genes originated through other duplication modes.

All predicted gene models for the 17 species were clustered using OrthoFinder (version 2.3.1)^22^, resulting in 22,455 orthogroups (Supplementary Tables 12 and 13). Using CAFÉ (version 4.0)^23^, 1515 gene families (6,754 genes) were found to be expanded, while 1657 gene families were found to be contracted in *R. simsii*, with 57.6% of the expanded gene families (EGFs) due to TD and PD duplications (Fig. 2b). Hypergeometric tests were performed for overlapping genes between expanded gene families and WGD-TD-PD-TRD-DSD (Fig. 2c). TD-EGFs and PD-EGFs genes were found exhibiting divergent enriched GO terms. For example, TD-EGFs enriched categories are implicated in plant self-defense, development and adaptation, while PD-EGFs genes are enriched in GO terms involved in ‘binding’ and ‘recognition’. In brief, newly generated tandem and proximal duplications have been important sources of gene family expansion in *R. simsii* (Supplementary Fig. 14).

To verify whether the identified tandem gene clusters are real and not artificial due to errors in the genome assembly, we mapped our PacBio long-reads back to the assembly, and examined whether the pair of adjacent tandem duplicated genes or the intergenic region could be fully or partially recovered by the mapped long-reads. We indeed found that most (79–87%) of the duplicated genes could be recovered, fully or partially, both for transcription factor (TF) or anthocyanin/flavonol biosynthetic genes (Supplementary Fig. 15). These results provide very good evidence for the true existence of tandem gene clusters.

### Flowering-time genes in *R. simsii*

We detected 424 genes related to flowering-time control in *R. simsii* by querying the Flowering Interactive Database, FLOR-ID^24^, in which 295 flowering-time genes are functionally characterized for *Arabidopsis*. With regards to gene function, the categories ‘General’ and ‘Photoperiodism, light perception and signaling’ were represented in large proportions of 37.26% (158) and 20.99% (89) among all flowering-time control genes (Fig. 2d). There was a clear time-gradient of expression of the flowering-time related genes (Supplementary Fig. 16) across the five examined flower coloring stages. In addition, we identified a gene family (OG0000614) encoding 13 members of high-affinity sucrose transporters that may play key roles in flowering transition delay^25^. This gene family was identified by CAFÉ as being expanded in azalea, and its members showed continuous upregulation in the corolla during the flowering time-series (Supplementary Fig. 16).

### Flower pigmentation genes in *R. simsii*

**Fig. 3:**
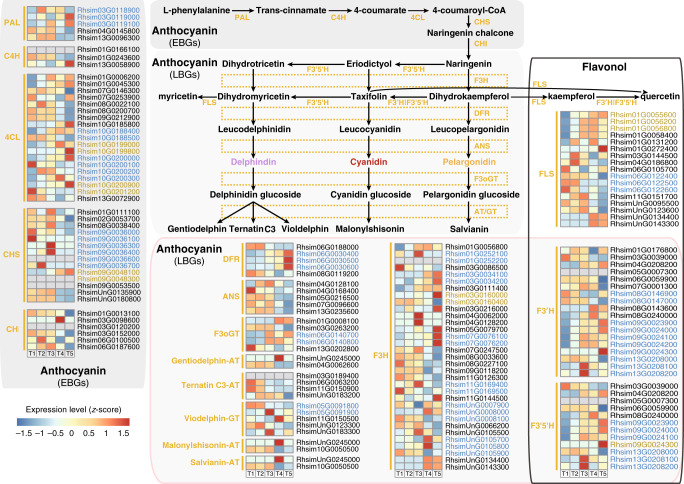
The biosynthesis pathways of two kinds of flavonoids, anthocyanin and flavonol. Genes identified in tandem clusters are displayed as blue while proximal cluster genes are displayed as brown. We divided the flavonoid enzymes encoding genes into two groups designated as ‘early’ and ‘late’, respectively^70^. The ‘early’ biosynthesis genes (EBGs) encode enzymes functioning more upstream towards the entrance into the pathway, whereas the ‘late’ biosynthesis genes (LBGs) exhibit highly distinct expression patterns. PAL phenylalanine ammonia-lyase (EC 4.3.1.24), C4H: cinnamate-4-hydroxylase (EC 1.14.14.91), 4CL 4-coumarate CoA ligase 4 (EC 6.2.1.12), CHS chalcone synthase (EC 2.3.1.74), CHI chalcone isomerase (EC 5.5.1.6), F3H flavanone 3-hydroxylase (EC 1.14.11.9), F3′H flavonoid 3′-hydroxylase (EC 1.14.13.21), F3′5′H flavonoid 3′,5′-hydroxylase (EC 1.14.13.88), DFR dihydroflavonol 4-reductase (EC 1.1.1.219), ANS anthocyanidin synthase (EC 1.14.11.19), F3oGT flavonol-3-O-glucosyl transferase (EC 2.4.1.115), AT acyltransferase, GT glucosyltransferase, FLS flavonol synthase (EC 1.14.11.23). Gene expression profile (in normalized TPMs) in different time points of flowering (here T1-T5, from left to right in each heatmap panel) are presented in the heatmap alongside the gene names. The bar represents the expression level of each gene (*z*-score). Low to high expression is indicated by a change in color from blue to red.

Our high-quality genome assembly allowed reconstruction of the metabolic pathway for flower coloration, specifically capturing the implicated enzymatic genes in this process. We unveiled 58 genes encoding enzymes functioning in the carotenoid biosynthesis pathway (Supplementary Fig. 17) and 125 enzymatic genes with predicted functions in anthocyanin and flavonol biosynthesis (Fig. 3 and Supplementary Fig. 18). Flavonoids are synthesized by a branched pathway that yields both colored anthocyanin pigments and colorless flavonols. Furthermore, we predicted five genes encoding three enzymes of blue-anthocyanins modification, including one anthocyanin 5-(6”‘-hydroxycinnamoyltransferase) (5AT|3AT, one of gentiodelphin-acyltransferase, EC 2.3.1.153), two ternatin C3-acyltransferases (ternatin C3-AT, EC 3.2.2.24), and two viodelphin-glucosyltransferases (viodelphin-GT, EC 2.3.1.-) (Fig. 3 and Supplementary Fig. 18).

### Gene duplication events linked to flowering time and color

TD genes were found to be enriched for GO terms related to secondary metabolic biosynthetic and modification processes (Fig. 2c). Furthermore, the proportion of TD/PD genes linked to flower coloration was significantly higher in *R. simsii* than that in related species (Supplementary Table 14). Counting the number of enzymatic genes in tandem or proximal gene clusters unveiled that TD/PD duplications substantially contributed to the observed proportions of enzymatic genes for the anthocyanin/flavonol versus the carotenoid biosynthesis pathway. TD/PD duplications affected 17% of genes for carotenoid biosynthesis in the *R. simsii* genome (Supplementary Fig. 17 and Supplementary Table 15) and 42% of the genes for anthocyanin/flavonol biosynthesis (Fig. 3 and Supplementary Table 15), respectively. In addition, important TD/PD events were also found for 52.63% (10/19) of the genes coding for 4CL (4-coumarate: CoA ligase 4; EC 6.2.1.12), 57.14% (8/14) encoding CHS (chalcone synthase; EC 2.3.1.74), 60% (3/5) encoding DFR (dihydroflavonol 4-reductase; EC 1.1.1.219), 48.48% (16/33) encoding F3H (flavanone 3-hydroxylase; EC 1.14.11.9), 35.29% (6/17) encoding FLS (Flavonol synthase; EC 1.14.11.23), 55.56% (10/18) encoding F3′H (flavonoid 3′-hydroxylase; EC 1.14.13.21), 58.33% (7/12) encoding F3′5′H (flavonoid 3′,5′-hydroxylase; EC 1.14.13.88) (Fig. 3). The diversity of the expression patterns indicates that further studies are necessary to verify and characterize the functions of the TD/PD duplicates.

In contrast, TD and PD duplications seem to have accounted for much lower proportions of expansions of flowering-time genes (6.82% (30/424) and 6.84% 424), respectively)/424), respectively), while WGD appears to have contributed the largest proportion (159/424, 37.5%) of flowering-time control gene copies and more than other modes of gene duplication (Fig. 2d). Interestingly, genes involved in flowering-time control are mostly present as low-copy gene families, 101 genes from single-copy families, 128 genes from dual-copy families and 60 genes from triple-copy families (Supplementary Fig. 19).

### The dynamics of flower pigmentation regulation in *R. simsii*

**Fig. 4:**
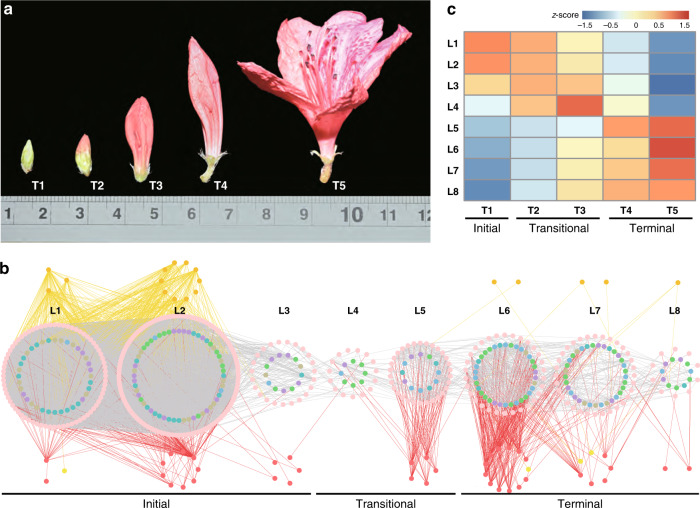
Time-ordered gene co-expression network related to flower coloring. **a** Five flower developmental stages (T1-T5). **b** Predicted regulatory network and the connection among TFs and structural genes involved in carotenoid and anthocyanin/flavonol biosynthesis pathways. Inside the pink circles, purple nodes represent *MYB* genes, olive nodes represent *bHLH*, turquoise nodes represent *WD40* genes, wathet blue nodes represent *WRKY* genes, and green nodes represent *ERF* genes. Pink nodes represent remaining TFs. Outside pink circles, orange points represent the enzymatic genes of the carotenoid biosynthesis pathway, locating on the top, crimson points and green points on the bottom represent enzymatic genes involved in anthocyanin and flavonol biosynthesis. L1 to L8 indicate the levels identified in the f-ordered gene co-expression network. **c**, the heatmaps of average normalized TPMs (*z*-score) at each time point of flowering at each level identified in the time-ordered gene regulatory network. Three stages of flower coloring were identified as the initial (T1), transitional (T2–T3) and terminal (T4–T5) stages, based on the expression profile. The bar represents the expression level of each gene (*z*-score). Low to high expression is indicated by a change in color from blue to red.

We performed time-ordered comparative transcriptome analyses (Fig. 4a and Supplementary Fig. 20) and found 8067 genes (618 TFs and 7,449 structural genes) expressed with an average Transcripts Per Kilobase Million (TPM) greater than 0.5 and exhibiting significant differentiation between any two samples among the five flowering time stages (T1–T5). As the initial node, a basic helix-loop-helix (bHLH) transcription factor (*Rhsim13G0024200*), highly expressed at the very first time point but not later, was selected to generate a time-ordered gene co-expression network (TO-GCN)^26^. Eight time-series expression levels (L1-L8, nodes >20) centering on TFs were finally reconstructed using the suggested^26^ positive/negative cutoff values (0.81; −0.57) (Fig. 4b). TO-GCN revealed a co-expression network involving the 618 differentially expressed TFs and 62 genes from the carotenoid/anthocyanin/flavonol biosynthesis pathways (Fig. 4b). Time-course transcriptomes were further distinguished for the initial (T1, flower appears white and semi-transparent), the transitional (T2 and T3 for light red flower), and the terminal (T4 and T5 for the determined red flower color) stages of corolla pigmentation (Fig. 4c).

The general pattern elucidated that most network members (358, including 328 TFs) appeared at the initial stage (T1), 177 genes (including 150 TFs) at the terminal stage (T4–T5) and 70 genes (65 TFs) at the transitional stage (T2–T3). Likewise, T1 (corresponding to time-series levels L1–L3) showed the highest TPM, followed by genes assigned to the transitional stage (T2–T3, corresponding to L4–L5), and finally the genes that were expressed at the terminal red color determining stage (T4–T5, corresponding to L6–L8). Functional enrichment analysis revealed that distinct gene functions are turned on and off, respectively, at these different stages (Supplementary Fig. 21). Yet, some genes encoding key biosynthetic enzymes in color determination tend to be continuously upregulated, such as those in the carotenoid biosynthesis pathway encoding phytoene synthase (PSY; EC 2.5.1.32), carotene isomerase (CRTISO; EC 5.2.1.13) (Supplementary Fig. 17), and anthocyanidin synthase (ANS; EC 1.14.11.19) in the anthocyanin biosynthesis pathway (Fig. 3).

**Fig. 5:**
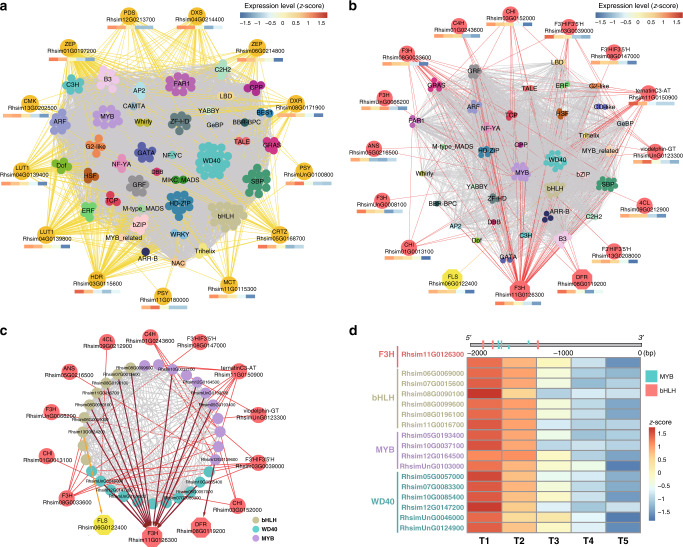
Sub-network for different pigment biosynthesis pathways in the initial stage. **a** Sub-network for carotenoid biosynthesis. **b** Sub-network for anthocyanin and flavonol biosynthesis. **c** Sub-network for anthocyanin and flavonol biosynthesis with *MYB*, *bHLH*, and *WD40* gene families. **d** Heatmaps of average normalized TPMs (*z*-score) at each time point of flowering. *F3H*, a gene encoding a key enzyme in the synthesis of dihydrokaempferol for flavonols/anthocyanins metabolic pathways, and 16 TFs (six *bHLH*, four *MYB*, and six *WD40*) identified as its direct regulators. The bar represents the expression level of each gene (*z*-score). Low to high expression is indicated by a change in color from blue to red. Edges between structural genes were not shown.

More specifically, at the initial stage (T1), we found that 14 enzymatic genes of the carotenoid biosynthesis expressed at high levels (Fig. 5a and Supplementary Table 16). Among the 194 TFs that appeared as potential direct regulators of these enzymatic genes, *WD40* family members were most numerous (21). For anthocyanin/flavonol biosynthesis, we detected 16 enzymatic genes expressed at high levels at T1 (Fig. 5b and Supplementary Table 16), and 120 TFs as their potential direct regulators. For genes implicated in the sub-network (Fig. 5c) for anthocyanin/flavonol biosynthesis and their associated *bHLH*, *WD40*, and *MYB* TFs (Supplementary Table 17), a clear gene expression decline was evident (Supplementary Fig. 22), exemplified for some of these TFs and their direct target, a dihydrokaempferol biosynthetic gene *F3H* (*Rhsim11G0126300*) (Fig. 5b–d).

**Fig. 6:**
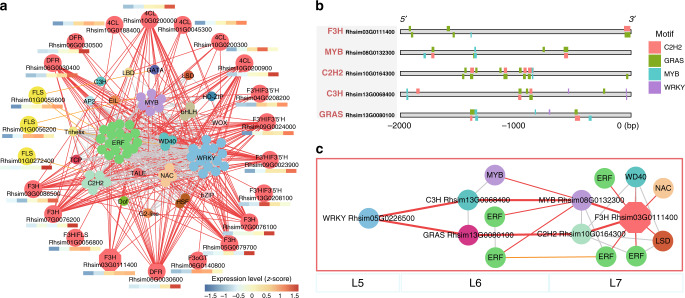
Sub-network for different pigment biosynthesis pathways in the terminal stage. **a** Sub-network for anthocyanin and flavonol biosynthesis. **b** DNA binding sites detected in the 2 Kb upstream sequences of five core genes and the potential regulatory elements (here TFs). **c** Resolved hierarchical regulation for *F3H* gene. The bar represents the expression level of each gene (*z*-score). Low to high expression is indicated by a change in color from blue to red. Edges between structural genes were not shown.

The terminal coloration stage (T4–T5) showed maximal expression for substantial numbers of TFs and pigment biosynthetic genes. For anthocyanin/flavonol biosynthesis, we predicted that seven enzymatic genes (Supplementary Table 16 and Fig. 6a) expressed at high levels were directly regulated by 74 potential regulators, mostly ethylene-responsive element binding factors (*ERF*s) (19) and *WRKY* (16) family members. The phylogenies, conserved motifs and gene structures of these TFs associated with flower coloration were further examined (Supplementary Figs. 23 and 24).

F3H represents an important rate-limited enzyme in anthocyanins/flavonols synthesis, but its upstream regulators have remained elusive. By examining the co-expression network inferred from TO-GCN (Fig. 6b, c), we identified the potential first-order to third-order upstream regulators. Here, we identified one *F3H* gene (*Rhsim03G0111400*) as the hub within the potential regulatory network relationships involving 14 TFs (Fig. 6c). We could infer that this *F3H* gene may be regulated in a hierarchical order by *WRKY Rhsim05G0226500* as the third regulator, either *GRAS Rhsim13G0080100* or *C3H Rhsim13G0068400* as the intermediate second regulator, and *MYB Rhsim08G0132300* or *C2H2 Rhsim10G0164300* as the direct regulator. When we incorporated the DNA binding site predictions, we found *C2H2* may be the sole intermediary regulator in the hierarchy, since only a C2H2 binding site was identified 5’ upstream of the *F3H* gene. By comprehensively examining the putative hierarchical gene regulation for all key enzymatic genes in anthocyanin/flavonol biosynthesis pathways (Fig. 6c and Supplementary Fig. 25), *WRKY Rhsim05G0226500* was identified as an important upstream regulator in these pathways.

## Discussion

We here describe a chromosome-scale genome assembly for a key parental species of the cultivated azalea, *Rhododendron simsii* (Ericales). In the asterids clade of the eudicot plants, representing ~25% of all flowering plant species^27^, we dated a whole-genome duplication in the Ericales lineage around 78 Mya. We believe this WGD event to be shared between Ericaceae and Actinidiaceae, as also suggested previously by Wei et al.^20^, while Soza et al.^7^ even suggested this WGD to have occurred in the common ancestor of Ericaceae, Actinidiaceae, and Theaceae. TD/PD, known to act as important drivers to increase gene product dosage^28^ and to accelerate the metabolic flux for rate limiting steps in certain biosynthetic pathways^29^, were found to be highly enriched in certain gene families^30^. We found that TD/PD duplications in azalea have substantially contributed to the proportions of enzymatic genes for the anthocyanin biosynthesis pathway, highly suggestive of the important role of TD/PD events in shaping flower color diversity.

Genetic modification in pigment biosynthetic pathways would provide a powerful method for obtaining novel flower colors beyond genetic constraints that are difficult to overcome through conventional breeding. Previous studies have characterized individual genes encoding relevant enzymes and gene transcription factors underlying pigment formation for floral crops^4,31,32^. Nevertheless, despite the studies on pigment composition for *Rhododendron* dating back to the 1950s^3,33^, the molecular basis of azalea flower coloration has remained elusive^6^, since, so far, only preliminary expression analysis was performed and for a limited number of structural genes.

Based on the chromosome-scale genome sequence of *R. simsii* presented here, in combination with the functional mapping and time-ordered transcriptome analysis, we first reconstructed the entire metabolic pathways for anthocyanin/flavonol/carotenoids biosynthesis in *Rhododendron*. The resolved pathways unravel that a MYB-bHLH-WD repeat (MBW) complex^34^ composed of MYB, bHLH, and WD40 TFs collectively regulates flavonoids biosynthesis in flower tissues. Importantly, the time-ordered gene co-expression networks for the terminal stage of flower development show WRKY and ERF TFs at the center of the co-expression network, with all involved *WRKYs* belonging to Group II. We speculate that members of these two TF families, known to be responsible for biotic/abiotic stress responses^35^, may also be involved in flower pigment biosynthesis, and likely play vital roles in flower color intensity and patterning^36^.

Flower colors of potted azalea are restricted to red, pink, white, reddish-purple, and purple, and providing yellow floral color has been the dream of many azalea breeders. Here, we reconstructed the carotenoid biosynthesis pathway known to be responsible for yellow, orange, or red pigments, as well as several genes encoding key enzymes of blue-anthocyanin biosynthesis responsible for blue pigment, none of which has been reported in *Rhododendron*. Moreover, gene expression for the generation of myricetin, kaempferol, and quercetin was detected as representatives of flavonols, which may also be involved in the flower color formation of *Rhododendron*^33^. These findings imply that potted azalea has the genetic potential to produce yellow and even extremely rare flower colors, such as violdelphin, and the genetic basis for flower coloring in *Rhododendron* far exceeds our cognition and it has great potential as a model plant for flower color research.

This study uncovered that MBW complexes may regulate *F3H* to control the biosynthesis of dihydroflavonols and subsequently also control anthocyanin/flavonol metabolism, suggesting that *F3H* should be a key node gene in regulating flower color formation in azalea. Moreover, the gene co-expression networks for metabolic pathways revealed that a specific WRKY transcription factor may play a core role in regulating flower coloration in azalea. We present a co-expression network for flower coloration, and inferred the potential contribution of individual members of transcription factor gene families, as well as structural genes involved in this regulatory process, a result that will allow for further functional investigation.

In conclusion, the reference genome sequence presented in this study provides a key resource for the further development of hypotheses and techniques in plant biotechnology, such as molecular marker-assisted breeding and genome editing, which are necessary to aid *Rhododendron* horticulture research and increase the efficiency for *Rhododendron* breeding.

## Methods

### *Rhododendron simsii* plant material

We sequenced the entire genome of a 20-year-old *Rhododendron simsii* individual originating from Jingshan, Hubei Province, China, and performed de novo genome assembly (Supplementary Fig. 1). This shrub had been transplanted to the Botanical Garden, Institute of Botany, Chinese Academy of Science, Beijing, China, and from which different plant tissues were sampled: fully expanded leaves were used for genome sequencing library preparation, while flowers, young leaves, and young stems were sampled for RNA sequencing (RNAseq) in support of genome annotation. Tissues were immediately flash frozen and stored at −80 °C for subsequent nucleic acid extractions.

In addition, corolla samples of five developmental stages from five individuals of *R. simsii* of the wild population selected for genome sequencing were collected to unravel the gene regulatory network underpinning flower coloring. Fresh tissues were first stored in RNAlater (Ambion, Life Technologies, Austin, TX, USA), then conserved at −80 °C.

### Library construction and sequencing of *R. simsii*

Total DNA was isolated and extracted from the leaves using the DNeasy Plant Mini Kit (QIAGEN, Inc.) and then purified using the Mobio PowerClean Pro DNA Clean-Up Kit (MO BIO Laboratories, Inc.). For PacBio SMRT sequencing, sheared and concentrated DNA was used to construct sequencing libraries with 20-kb DNA inserts and subsequently run on a PacBio RSII platform using P6-C4 chemistry (6 SMRT cells). After data filtering and preprocessing, a total of 6.5 million PacBio long reads were generated, yielding ~50 Giga bases (Gb) (100× coverage) with an average read length of 7705 base pairs (bp).

For Illumina sequencing, 150-bp paired-end (PE) libraries were prepared for sequencing on an Illumina HiSeq X Ten platform. Short reads were processed with fastp (version 0.19.3)^37^. Finally, we obtained ~91.49 Gb of raw sequencing data (corresponding to roughly 170× the assembled genome).

One Hi-C library prepared with MboI restriction enzyme was sequenced on Illumina HiSeq X Ten to generate ~55.68 Gb of valid data from 150 PE reads. Different tissues (stem, leaf, and flower) were used for constructing mRNA sequencing libraries; 150 bp PE sequencing was performed on an Illumina HiSeq X Ten machine.

More details on sample preparation and sequencing are available in the Supplementary Note 2 and 3.

### De novo genome assembly of *R. simsii* and quality control

De novo genome assembly employed the three following steps: primary assembly, Hi-C scaffolding and polishing. First, eight versions of the primary assemblies were generated by SMARTdenovo (see “URLs” section), WTDBG (version 2.1)^38^, Canu (version 1.7)^39^ and FALCON-Phase (version 0.1.0-beta) (see “URLs” section) from PacBio long reads. Subsequently, we merged the two optimal assemblies, v0.3 (criteria: reasonably sized assembly, fewest contigs) and v0.4 (criterion: highest contig N50) by quickmerge (version 0.2)^40^, further polished with one round of pilon (see “URLs” section) using clean Illumina reads to generate assembly v1.0. Based on Hi-C data and assembly v1.0, primary scaffolds were produced by 3D-DNA (version 180922) (see “URLs” section). These scaffolds were roughly spilt by Juicebox (version 1.8) (see “URLs” section) and another round of scaffolding by 3D-DNA (version 180922). Afterwards, we elaborately optimized the new scaffolds with gap closing by LR_Gapcloser (version 1.1) (see “URLs” section) and five rounds of pilon polishing.

BUSCO and LAI were used to assess genome completeness and continuity. Furthermore, we mapped 51.15 Gb PacBio sequencing reads, 91.49 Gb Illumina reads, 403.67 Gb clean RNA-seq sequences, and 55.68 raw Hi-C data to the genome assemble using BWA-MEM (see “URLs” section), minimap2 (version 2.11-r797)^41^, HiSat2 (version 2.1.0) (see “URLs” section) and Juicer (see “URLs” section), respectively.

Additional details regarding genome assembly and quality control are provided in the Supplementary Note 4.

### Annotation of repeat elements and genes for *R. simsii*

Repeat elements were identified by a combination of homology-based and de novo approaches using RepeatModeler (version 1.0.10) (see “URLs” section) and RepeatMasker (version 4.0.7, rmblast-2.2.28) (see “URLs” section). Furthermore, we examined classification, age distribution, birth and death of LTR-RTs (17.01% of the annotated genome). In addition, amino acid sequences of intact RT domains of the *Copia* and *Gypsy* superfamily were retrieved to perform selection pressure analyses with codeml^42^.

De novo and reference genome-guided transcriptome assemblies were constructed and combined as evidence for coding gene prediction. Coding gene structures were predicted by MAKER2 pipeline^43^ applied to the repeat-masked genome with three main approaches (ab initio predictions, homolog proteins and transcriptome data). Non-coding RNAs (ncRNAs) were annotated using several databases and software packages: tRNAscan-SE (version 1.3.1)^44^, RNAmmer (version 1.2)^45^, Rfam database (version 9.1) (see “URLs” section), and BLASTn (version 2.2.28+). Gene functions were annotated according to both homology and similarity searches by blat (version 36)^46^ with 30% identity and 1e−05 *E*-value cutoff and domain similarity prediction strategies using InterProScan (version 5.27-66.0) (see “URLs” section).

Locations of all centromeres within the genome were inferred with Centurion^47^ based on corrected Hi-C data and the tendency of formed clusters in three-dimensional space.

Additional details are available in the Supplementary Note 5.

### Inference and analysis of orthogroups for asterids

To retrieve the evolutionary history of the asterids clade, a total of 22,455 orthogroups of azalea and 16 representative plant species (Supplementary Tables 12 and 13) were identified by using OrthoFinder (version 2.3.1)^22^. Then, OrthoFinder provides a formalized procedure for determining 806 low-copy orthologs with minimum of 76.5% of species having single-copy genes in any orthogroup. The concatenated amino acid sequences alignment was created by MUSCLE (version 3.8.31)^48^ and trimmed with trimAI (version 1.2) (trimal -gt 0.8 -st 0.001 -cons 60)^49^. And then a maximum likelihood (ML) phylogenetic tree was constructed using IQ-TREE (version 1.6.7)^50^ with the selected optimal sequence evolution model (-m JTT + F + R5) and with ultrafast bootstrapping (-bb 1000)^51^ using *Vitis vinifera* and *Arabidopsis thaliana* as outgroups. The ML tree was then used as an input tree to estimate the divergence time using the MCMCTree program in the PAML package (version 4.9 h)^42^ with two fossils and a soft bound at three nodes: (1) the stem node of *Rhododendron* (56 Mya)^52^, (2) the crown node of Ericales (89.8 Mya)^53^, (3) asterids-rosids (116–126 Mya)^54^ as constraints for calibrating the age of our tree. The expansion and contraction of gene families were inferred with CAFÉ (version 4.1)^23^ based on the chronogram of these 17 species.

### Genome duplication and intergenomic comparison for asterids

To investigate genome evolution across asterids, we searched for genome-wide duplications in the order Cornales and Ericales. First, we identified different modes of gene duplication as whole-genome duplicates (WGD), tandem duplicates (TD), proximal duplicates (less than 10 gene distance on the same chromosome: PD), transposed duplicates (transposed gene duplications: TRD), or dispersed duplicates (other duplicates than WGD, TD, PD and TRD: DSD) using DupGen_finder^55^ with default parameters. Second, the *K*a (number of substitutions per nonsynonymous site), *K*s (number of substitutions per synonymous site), and *K*a/*K*s values were estimated for gene pairs generated by different modes of duplication based on the YN model in KaKs_Calculator (version 2.0)^56^, after conversion of amino acid alignments into the corresponding codon alignments with PAL2NAL (version 14)^57^.

For intergenomic comparison, we compared *Vitis vinifera* genome with five asterids species (*Camellia sinensis*, *Camptotheca acuminata*, *Rhododendron delavayi*, *R. williamsianum*, *R. simsii*), and also compared *R. simsii* genome with that of *Actinidia chinensis* and a haplotype of *Vaccinium corymbosum*. The orthologs between these species were identified using MCScanX_h^58^. Subsequently, *Ks* substitution rates of gene pairs across syntenic blocks were calculated. Finally, we illustrated *Ks* distribution and the dotplots of orthologous blocks using MCScanX^58^.

WGD time was estimated with asterids-rosids divergence time (mean: 121 Mya)^54^ as an age constraint. *K*s peaks of *V. vinifera* vs. five species (*Camellia sinensis*, *Camptotheca acuminata*, *R. delavayi*, *R. williamsianum*, and *R. simsii*) syntenic orthologs allows calculating *Ks* per year (*r*) following *r* = *K*s/(2 × (divergence time))^16^. We applied the same *r* value to calculate the time of WGD events for each species.

### Flowering time and flower color genes

We searched for homologs of flowering time control genes in *A. thaliana* by querying FLOR-ID^24^, a recently developed database which categorizes 295 protein-coding and 11 miRNA genes and describes their interactions within Arabidopsis’ genome. Flower color is mainly determined by anthocyanins and carotenoids^4,5,32^. Given the importance of anthocyanins/carotenoids accumulation in *R. simsii*, we annotated genes associated with the anthocyanins/carotenoids biosynthesis pathways by querying the Plant Metabolic Network^59^ and validated by Semi-Automated Validation Infrastructure (version 3.02)^59^, after enzymatic protein annotations for coding genes through the Ensemble Enzyme Prediction Pipeline (E2P2) package (version 3.1) (see “URLs” section).

### *R. simsii* transcription factors

We used PlantRegMap^60^ to identify TFs in the *R. simsii* genome. Among all identified transcription factors, we further analyzed the phylogeny, gene conserved motifs, and protein structures of MYB, bHLH, WD40, WRKY and ERF. Additional details are available in the Supplementary Note 6 and 7.

### Metabolic pathways construction based on TO-GCNs

In order to identify core genes of flower timing and color development, we performed a gene expression study using full transcriptome data. For the gene expression study, 25 frozen corolla tissues from five developmental stages and five-times replicates were ground with a mortar and pestle and RNA was isolated using the NEBNext Poly (A) mRNA Magnetic Isolation Module. RNA quality was determined on an Agilent 2100 BioAnalyzer. All of the 25 sequencing libraries were prepared using the NEBNext Ultra RNA Library Prep Kit for Illumina. Next, 150 bp PE mRNA sequencing was performed on an Illumina HiSeq X Ten. These Illumina reads were processed using Trimmomatic (version 0.36)^61^ and Cutadapt (version 1.13)^62^, and then mapped to the final assembly using HiSat2 (version 2.1.0). Only uniquely mapped paired-end reads were retained for read counting of the annotated genes by featureCounts^63^ to generate the count and Transcripts per Kilobase Million (TPM) tables. Differential gene expression (DEG) analyses between the five stages were performed with DEseq2^64^, with 0.05 as the FDR cut-off and a log2 fold change (FC) cut-off of 1 (Supplementary Fig. 20).

We used a recently developed method for reconstructing time-ordered gene co-expression networks (TO-GCNs^26^). Significantly differentially expressed genes with average TPM less than 0.5 were excluded. The Pearson correlations between 618 TFs and 7,449 genes less than −0.57 or above 0.81 in C1 + C2 + GCN were visualized in graphs using Cytoscape^65^.

Finally, 2 Kb sequences in the upstream of six core genes (*Rhsim03G0111400, Rhsim08G0132300, Rhsim10G0164300, Rhsim11G0126300, Rhsim13G0068400*, and *Rhsim13G0080100*) were extracted and queried against PlantRegMap and PlantCARE^66^. TF binding sites uncovered by PlantRegMap were illustrated with R package drawProteins^67^ (Figs. 5d and 6b). Additional details regarding TFs are provided in the Supplementary Note 8.

### Gene functional enrichment

Hypergeometric tests were performed to determine whether specific functional categories from GO were significantly overrepresented in *R. simsii* gene sets within the genome. Functional enrichment was tested using the R package clusterProfiler (version 3.6.0)^68^ with the background set of all annotated *R. simsii* genes in this study.

### URLs

SMARTdenovo [https://github.com/ruanjue/smartdenovo]; FALCON-Phase (version 0.1.0-beta) [https://github.com/WGLab/EnhancedFALCON]; Pilon [http://github.com/broadinstitute/pilon]; 3D-DNA (version 180922) [https://github.com/theaidenlab/3d-dna]; Juicebox (version 1.8) [https://github.com/aidenlab/Juicebox]; LR_Gapcloser (version 1.1) [https://github.com/CAFS-bioinformatics/LR_Gapcloser]; BWA-MEM [https://github.com/lh3/bwa]; HiSat2 (version 2.1.0) [https://github.com/infphilo/hisat2]; Juicer [https://github.com/aidenlab/juicer]; RepeatMasker [http://www.repeatmasker.org]; RepeatModeler [http://www.repeatmasker.org]; Rfam database (version 9.1) [http://eggnogdb.embl.de]; InterProScan (version 5.27-66.0) [http://www.ebi.ac.uk/InterProScan]; PMN Ensemble Enzyme Prediction Pipeline (E2P2, version 3.1) [https://gitlab.com/rhee-lab/E2P2]; Global Biodiversity Information Facility database [https://www.gbif.org/]; database of retrotransposon protein domains (REXdb) [http://repeatexplorer.org/]; Gypsy database [http://gydb.org]; Actinidia chinensis [ftp://ftp.ncbi.nlm.nih.gov/genomes/all/GCA/003/024/255/GCA_003024255.1_Red5_PS1_1.69.0/]; *Arabidopsis thaliana* [https://phytozome-next.jgi.doe.gov/info/Athaliana_TAIR10]; *Camellia sinensis* [http://tpia.teaplant.org/download.html]; Camptotheca acuminata [https://datadryad.org/stash/dataset/doi:10.5061/dryad.nc8qr]; *Coffea canephora* [http://coffee-genome.org/]; Daucus carota Daucus carota [https://phytozome-next.jgi.doe.gov/info/Dcarota_v2_0]; Eucommia ulmoides [ftp://download.big.ac.cn/gwh/Plants/Eucommia_ulmoides_hardy_rubberv0_GWHAAAL00000000/]; Helianthus annuus [ftp://ftp.ncbi.nlm.nih.gov/genomes/all/GCF/002/127/325/GCF_002127325.1_HanXRQr1.0]; Lactuca sativa [ftp://ftp.ncbi.nlm.nih.gov/genomes/all/GCF/002/870/075/GCF_002870075.1_Lsat_Salinas_v7]; Primula vulgaris [https://opendata.earlham.ac.uk/opendata/data/primula/sci_reports_Cocker_et_al_2018/]; Rhododendron delavayi [http://gigadb.org/dataset/100331]; Rhododendron williamsianum [https://genomevolution.org/coge/GenomeInfo.pl?gid=51210]; Sesamum indicum [http://ocri-genomics.org/Sinbase/]; *Solanum lycopersicum* [https://phytozome-next.jgi.doe.gov/info/Slycopersicum_ITAG2_4]; Vaccinium corymbosum [http://gigadb.org/dataset/100537]; Vitis vinifera [https://www.ncbi.nlm.nih.gov/assembly/GCF_000003745.3].

### Reporting summary

Further information on research design is available in the Nature Research Reporting Summary linked to this article.

## Supplementary information

Supplementary Information

Peer Review

Reporting Summary

## Data Availability

Data supporting the findings of this work are available within the paper and its Supplementary Information files. A reporting summary for this Article is available as a Supplementary Information file. The datasets generated and analyzed during this study are available from the corresponding author upon request. The raw sequence data of *R. simsii* genome sequencing and RNA sequencing have been deposited in NCBI under the accession number SRP229032 (Bio-Project PRJNA588298). The final assembly is available at DDBJ/ENA/GenBank under the accession number WJXA00000000. Genome assembly, repeat and gene annotation, gene expression profiles could be downloaded and explored online under URL http://rhododendron.plantgenie.org/. The source data underlying Figs. 1a, 2a, 2b, 2d, 3, 4b, 5d, 6b, and Table 1, as well as Supplementary Figs. 5, 13, 14, 16–18, 21, 23 and 24 are provided as a Source Data file. Source data are provided with this paper.

## Source data

Source Data
