# Supplementary material for: Chromosome-level genome assembly of a parent species of widely cultivated azaleas
Source: Nat Commun. 2020 Oct 19;11:5269. doi: 10.1038/s41467-020-18771-4 (PMC7572368; doi:10.1038/s41467-020-18771-4)
Supplement: Supplementary file 1 — Supplementary Information [file 41467_2020_18771_MOESM1_ESM.pdf]

Chromosome-level genome assembly of a parent species of widely cultivated azaleas

Yang *et al.*

Supplementary Figures

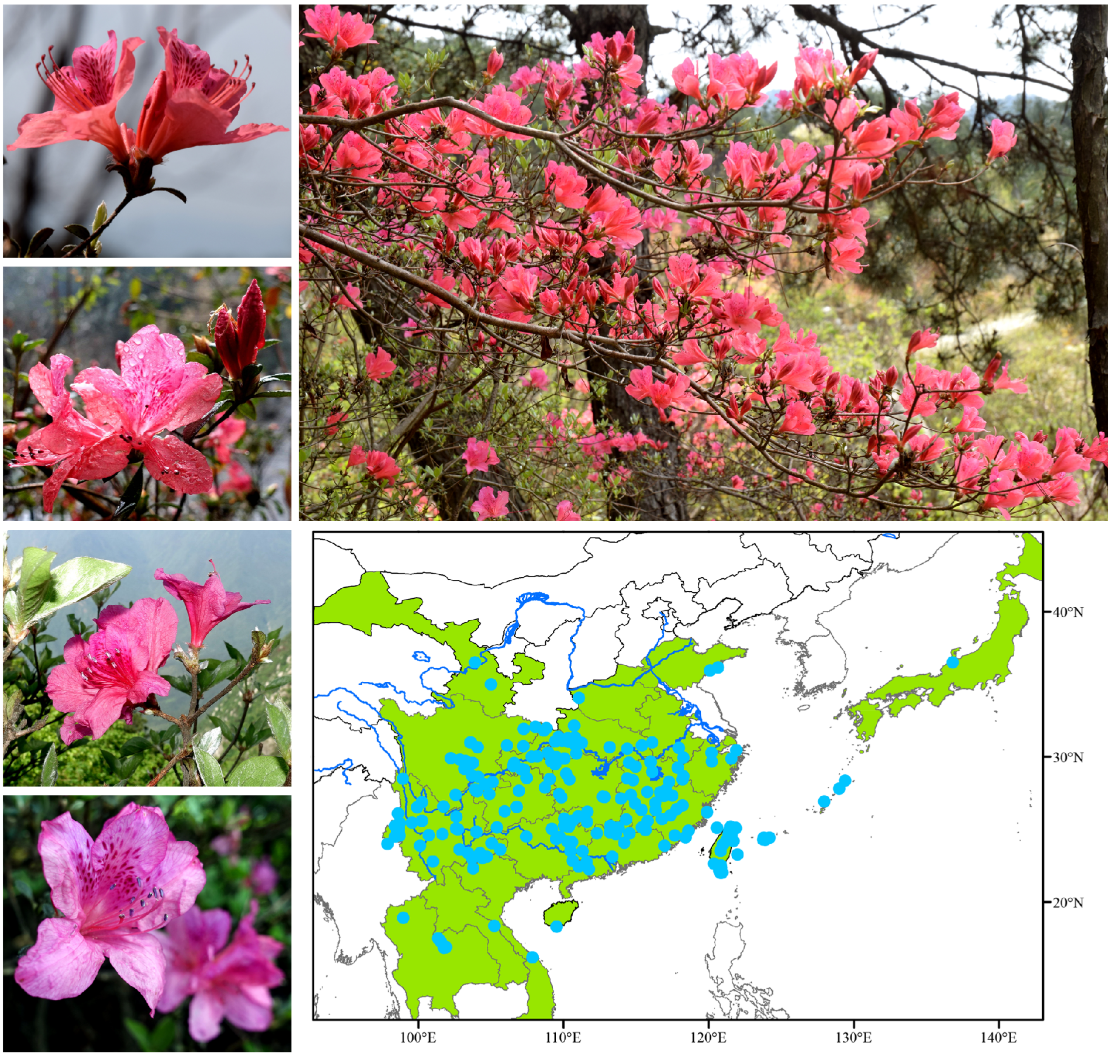

Supplementary Fig. 1. Flower and recorded natural distribution of *Rhododendron simsii*. Flowers were photographed (by Fu-Sheng Yang) in wild population from which an azalea individual was selected for whole genome sequencing. Distribution was determined by querying the Global Biodiversity Information Facility database (GBIF, https://www.gbif.org/), green color range indicates the countries and provinces where distribution record was found, while cyan points are the individual records.

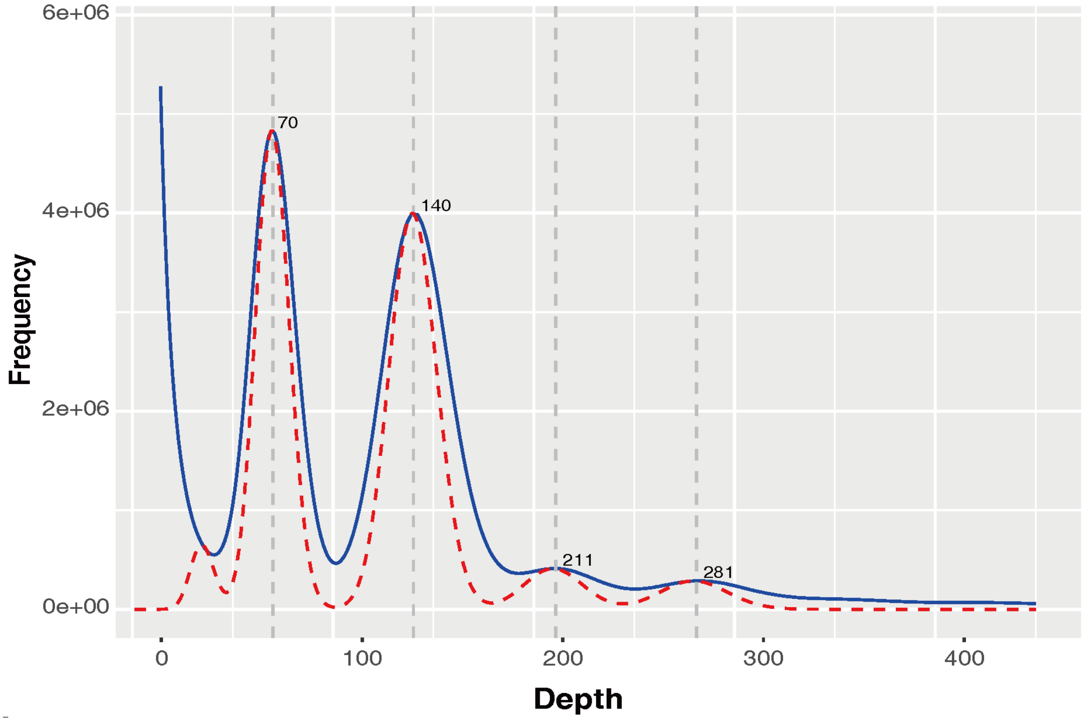

Supplementary Fig. 2. *K*-mer frequency distribution estimated from PacBio sequences after filtering and correction at *K*-mer size of 17. A *K*-mer refers to an artificial sequence division of *K* nucleotides. Genomic characteristics (genome size, repeat structure, and heterozygous rate) could be estimated based on *K*-mer frequencies. Blue solid line for observed *K*-mer frequency distribution, red dash line for fitted model of *K*-mer frequency distribution.

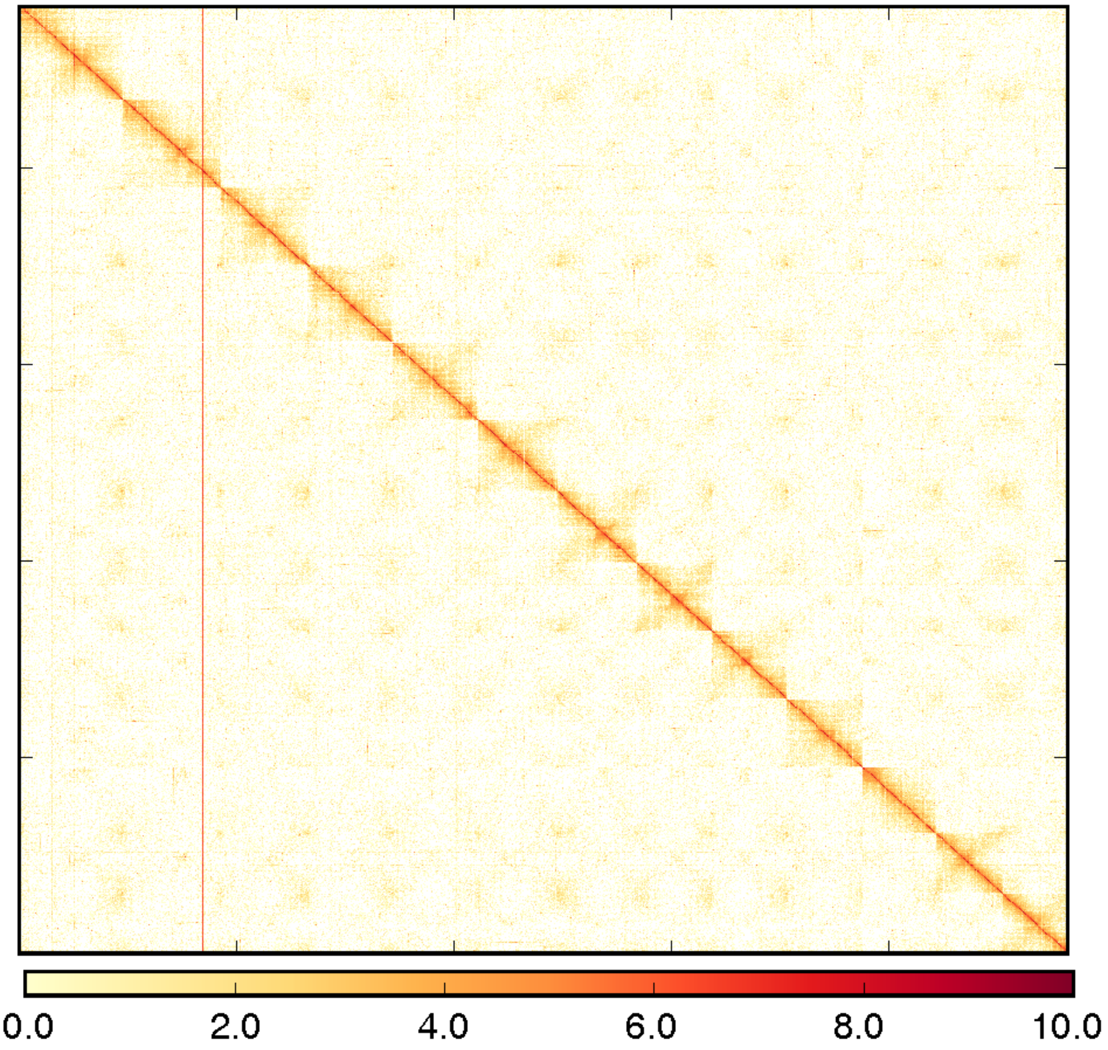

Supplementary Fig. 3. Genome-wide analysis of chromatin interactions in the genome based on Hi-C data.

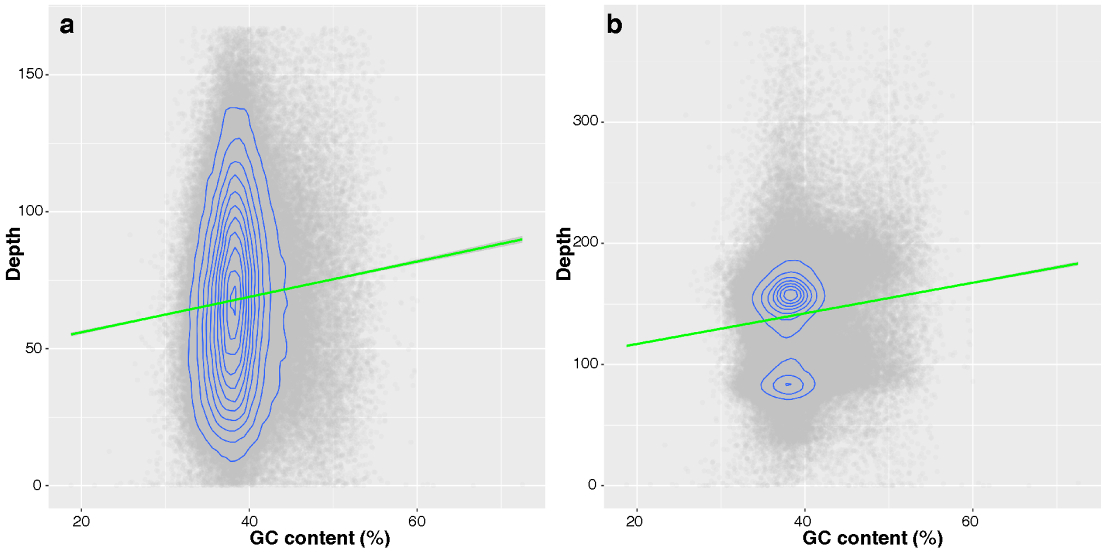

Supplementary Fig. 4. Sequence depth and GC content for PacBio SMRT sequencing and Illumina short-read sequencing. **a**: PacBio SMRT sequencing; **b**: Illumina short-read sequencing.

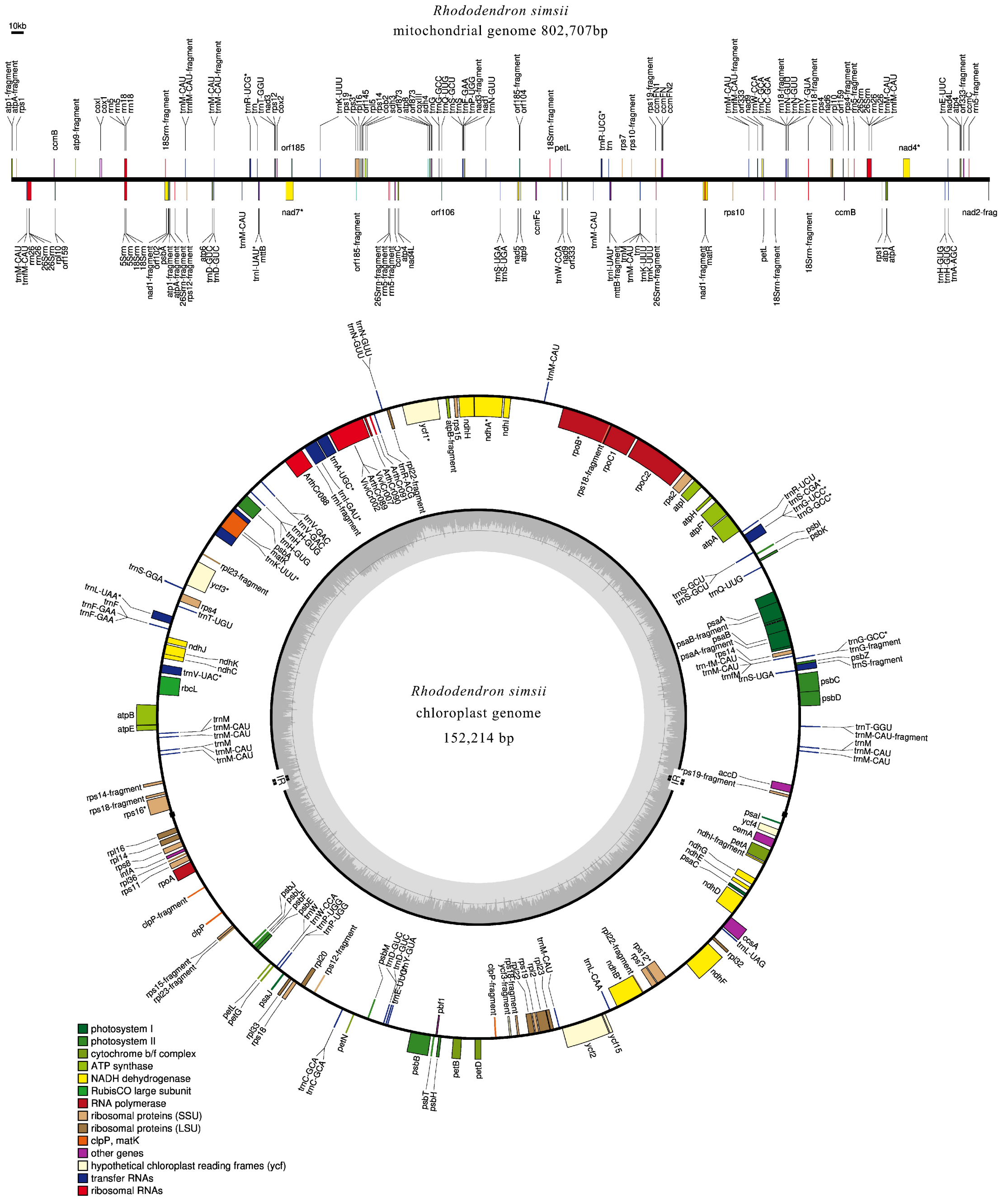

Supplementary Fig. 5. Annotation of chloroplast and mitochondrial assemblies. Source data are provided as a Source Data file.

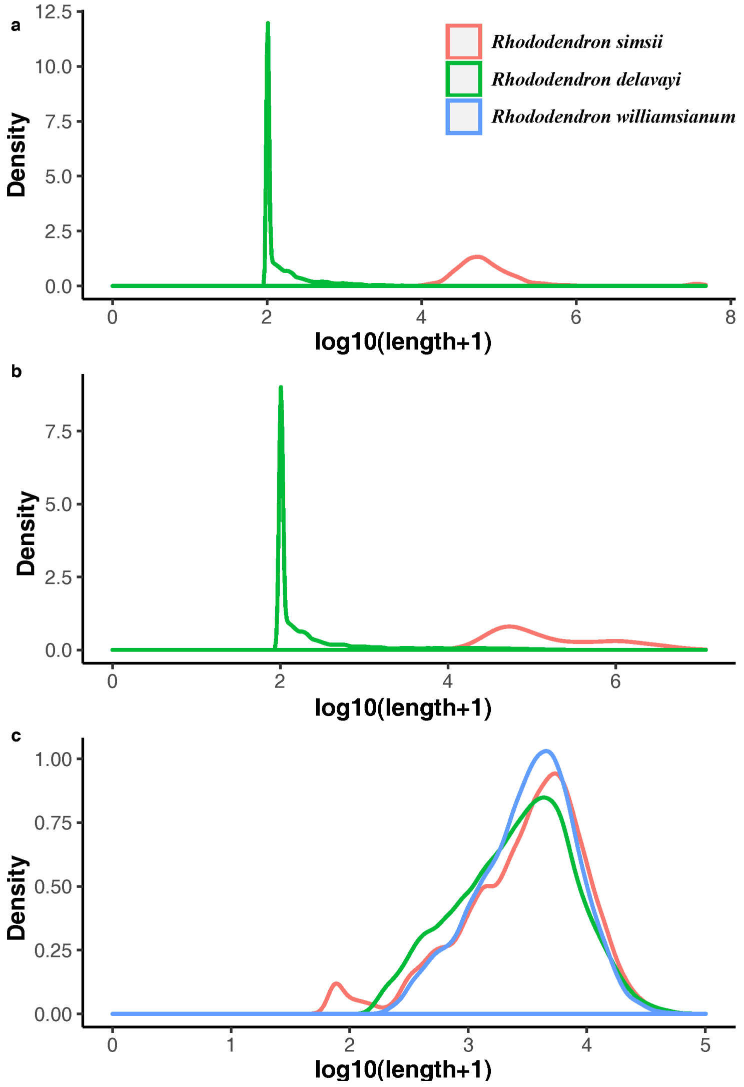

Supplementary Fig. 6. Length distribution of genes, contigs, and scaffolds between our assembly for *Rhododendron simsii* and the published assemblies for *R. delavayi* and *R. williamsianum* genomes. For the *R. williamsianum* genome, only the chromosome-level scaffolds were obtained. **a**: scaffold; **b**: contig; **c**: gene.

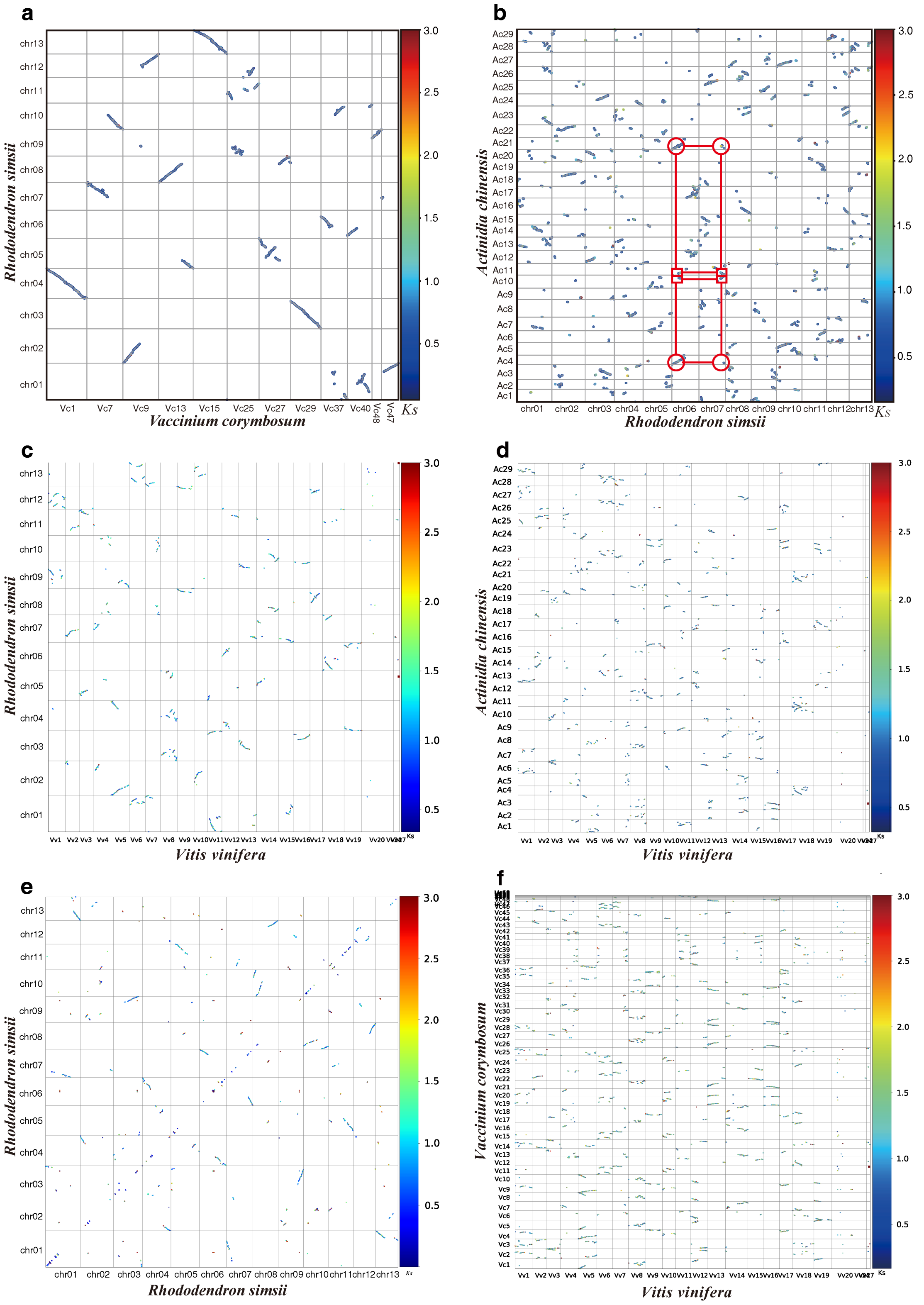

Supplementary Fig. 7. The dot plots of paralogous blocks between **a**: *Rhododendron simsii* and a randomly selected haplotype of *Vaccinium corymbosum*, **b**: *R. simsii* and *Actinidia chinensis,* (4:2 chromosomal relationships in red circles), **c**, *Vitis vinifera* and *R. simsii*, **d**: *V. vinifera* and *A. chinensis*, **e**: *R. simsii* and *R. simsii*, **f**: *V. vinifera* and *V. corymbosum*.

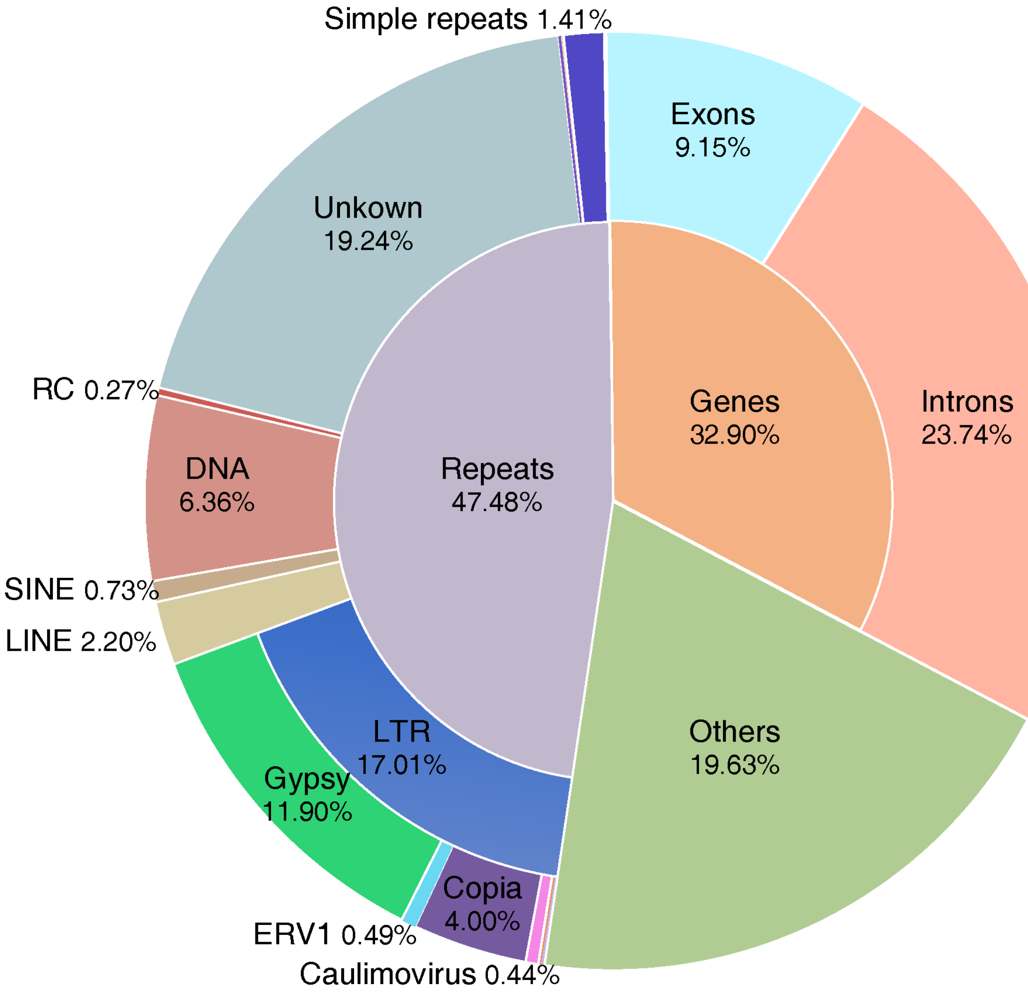

Supplementary Fig. 8. Genome proportions of various types of repeat sequences. Some types of repeats with low proportions (< 0.2%) are not shown.

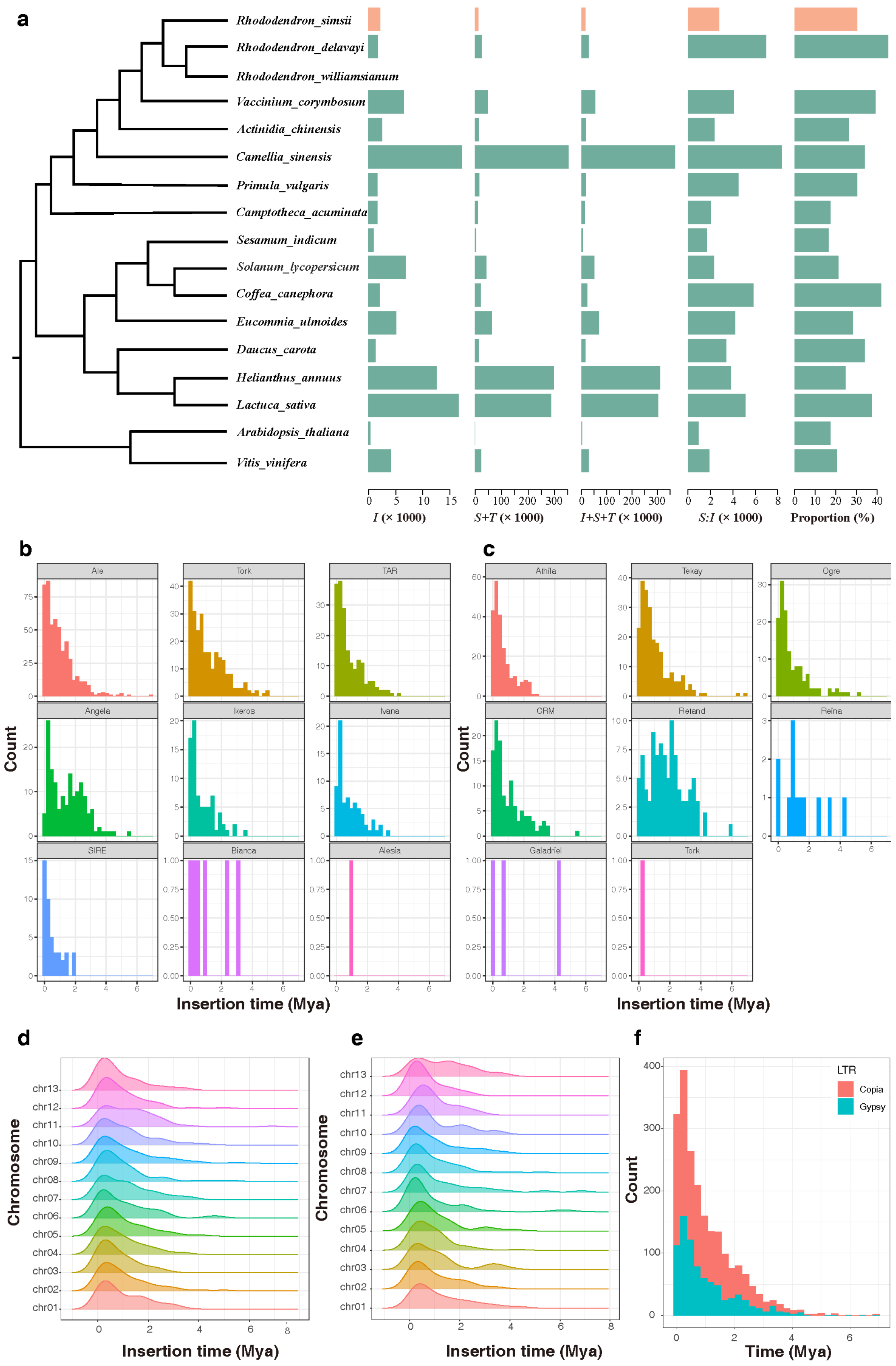

Supplementary Fig. 9. Proliferation history of different superfamilies of the *Gypsy* and *Copia* classes of LTR-RTs (long terminal repeat-retrotransposons). a: Birth and death of LTR-RTs. I: intact LTR-RT, S: solo-LTR, T: truncated LTR-RT, LTR-RT accumulation (S+T+I) and proportions of LTR-RTs found in the clusters with high removal rates (filtered S:I $\geq$ 3) are illustrated on the rightmost column; **b**: Insertion time of *Copia* family on subfamily levels; **c**: Insertion time of *Gypsy* family on subfamily levels; **d**: Insertion time of the *Gypsy* family in the centromeric regions on each chromosome; **e**: Insertion time of the *Copia* family in the centromeric regions on each chromosome; **f**: Insertion time of *Gypsy* and *Copia* families on family levels.

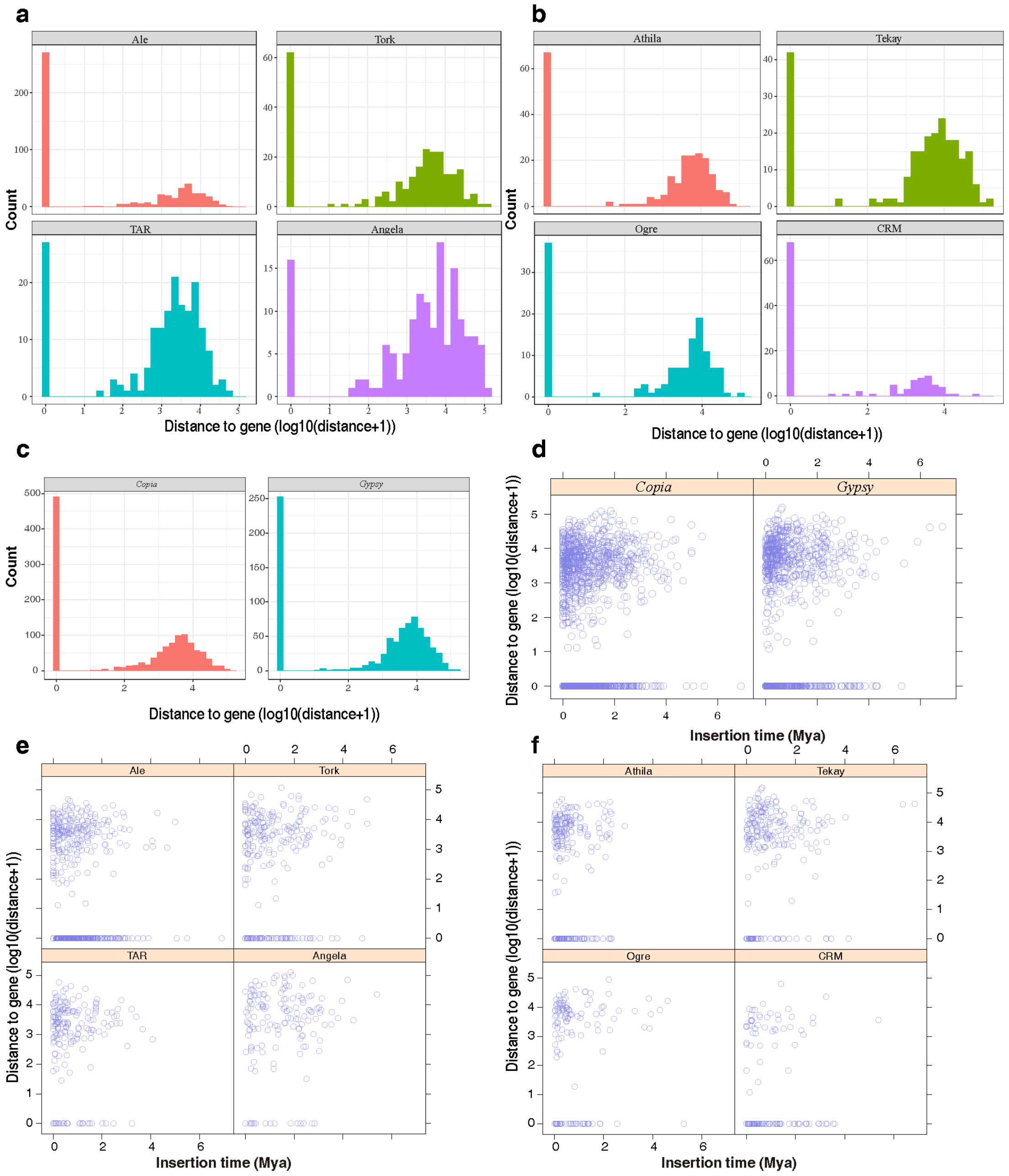

Supplementary Fig. 10. Gene proximity and insertion time for different superfamilies of the *Gypsy* and *Copia* classes of LTR-RTs. **a**: Gene proximity of *Copia* family on subfamily levels; **b**: Gene proximity of *Gypsy* family on subfamily levels; **c**: Gene proximity of *Gypsy* and *Copia* families on family levels; **d**: Gene proximity and insertion time for the *Copia* and *Gypsy* families; **e**: Gene proximity and insertion time for major superfamilies of the *Copia* family; **f**: Gene proximity and insertion time for major superfamilies of the *Gypsy* family; Distance to gene (log10(distance+1)): The natural logarithm of the base distance between an LTR-RT and an adjacent gene (plus one).

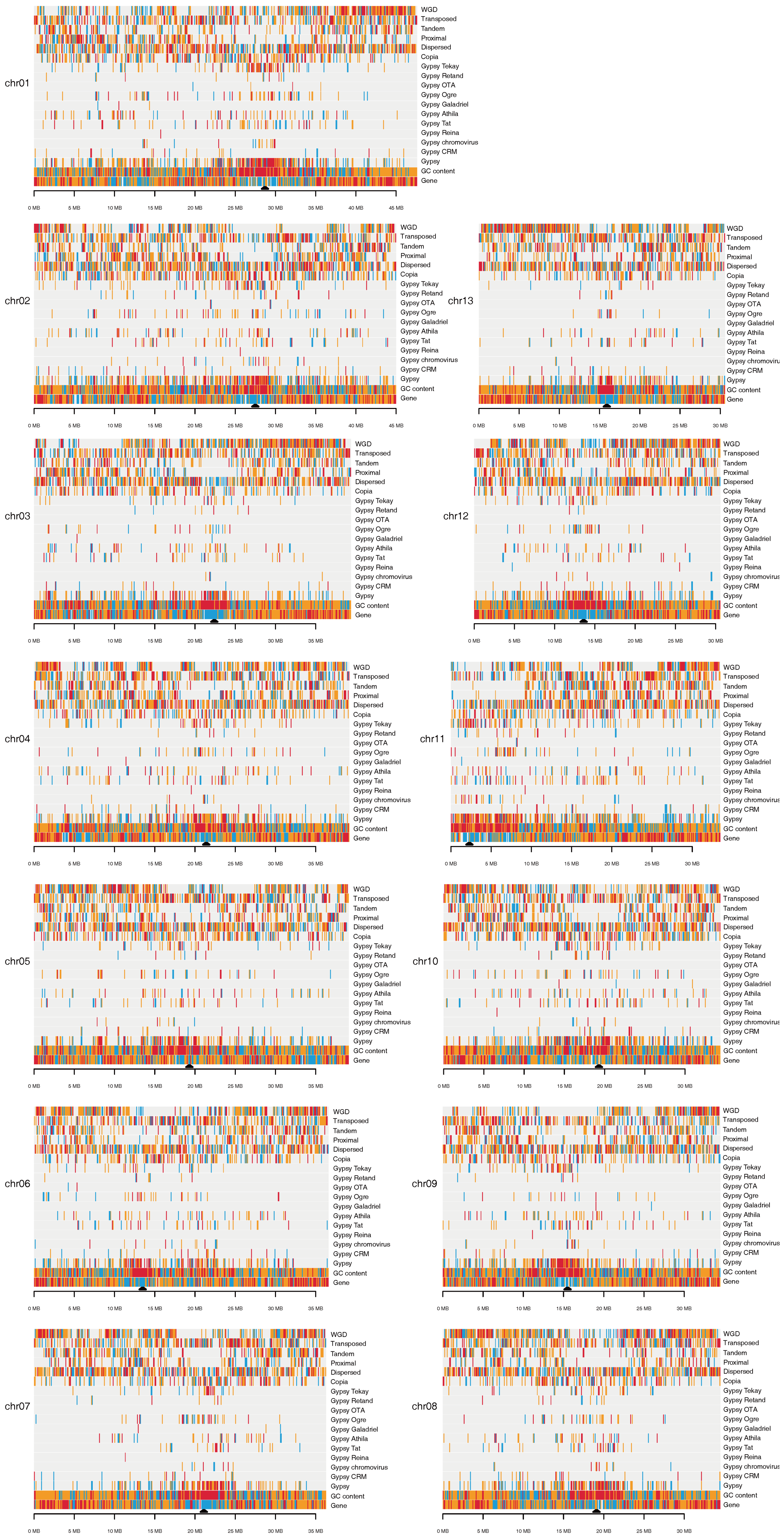

Supplementary Fig. 11. Distribution of different genomic features along the chromosomes. Five modes of gene duplications (WGD, TSD, TD, PD and DSD), *Copia* family, *Gypsy* family and subfamilies, GC content and genes along 13 chromosomes. Recognized as a bin per 0.1 Mb; colors determined by quartile within bin, 0 is light gray, light blue for less than 25%, red for greater than 75%, orange for 25%-75%; black triangles represent the centromeres.

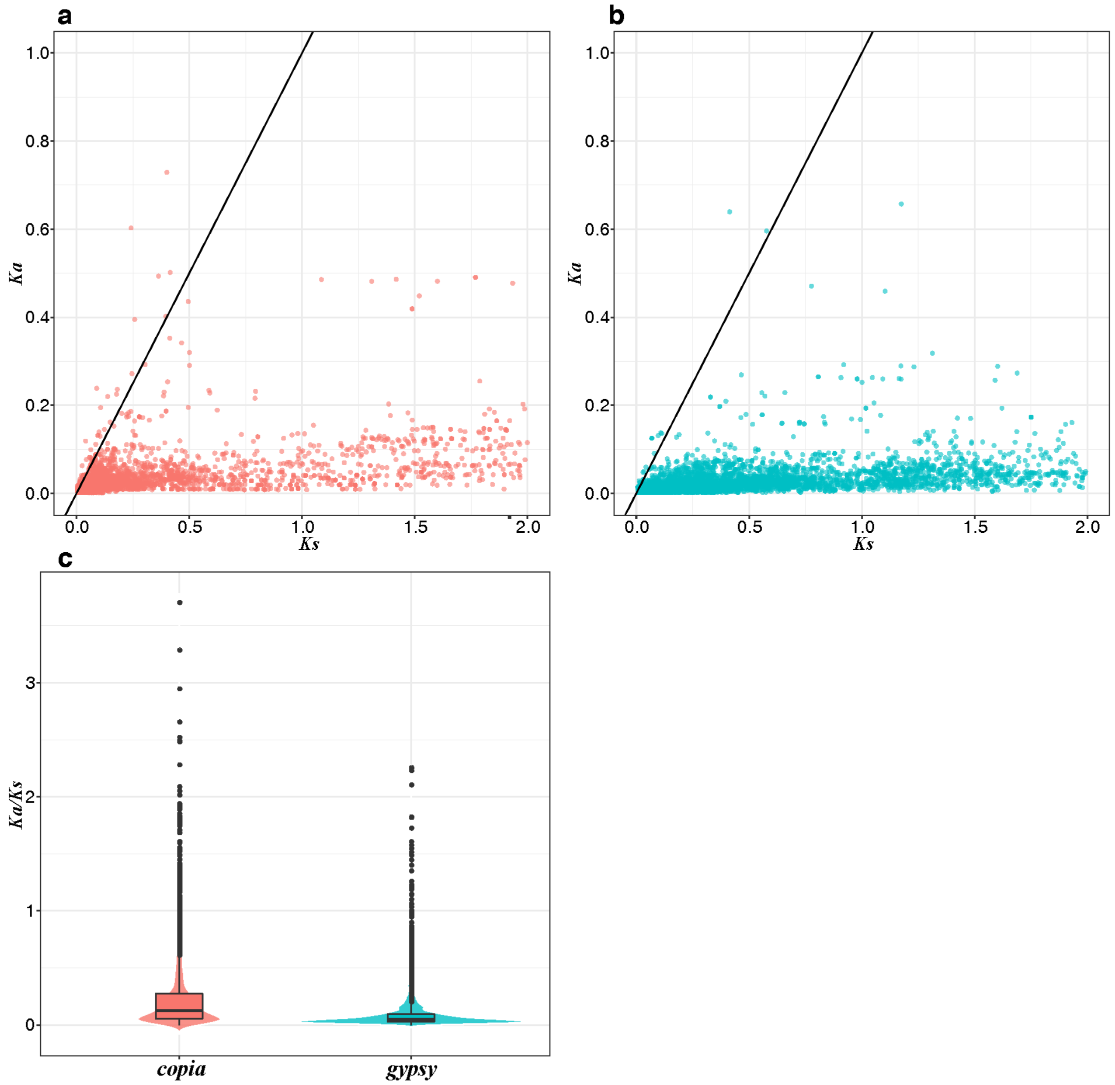

Supplementary Fig. 12. Distribution of *K*a/*K*s, *K*a and *K*s of amino acid sequences of intact RT domains of the *Copia* and *Gypsy* classes of LTR-RTs (long terminal repeat-retrotransposons). a: *K*a and *K*s distribution of *Copia*; **b**: *K*a and *K*s distribution of *Gypsy*; **c**: *K*a/*K*s distribution of *Copia* and *Gypsy*. In the boxplot, points are outliers; center line represents median; The lower and upper hinges correspond to the first and third quartiles (the 25th and 75th percentiles); whiskers extend to the minimum (left whiskers) and maximum (right whiskers) estimates located within 1.5× interquartile range (IQR) from the first and third quartiles, respectively. Gaussian kernel estimates of *K*a/*K*s were shown as violins, 1,303 *Copia* and 825 *Gypsy* were incorporated in the statistics. Black lines in a and b represent *K*a/*K*s = 1 (*K*a = *K*s).

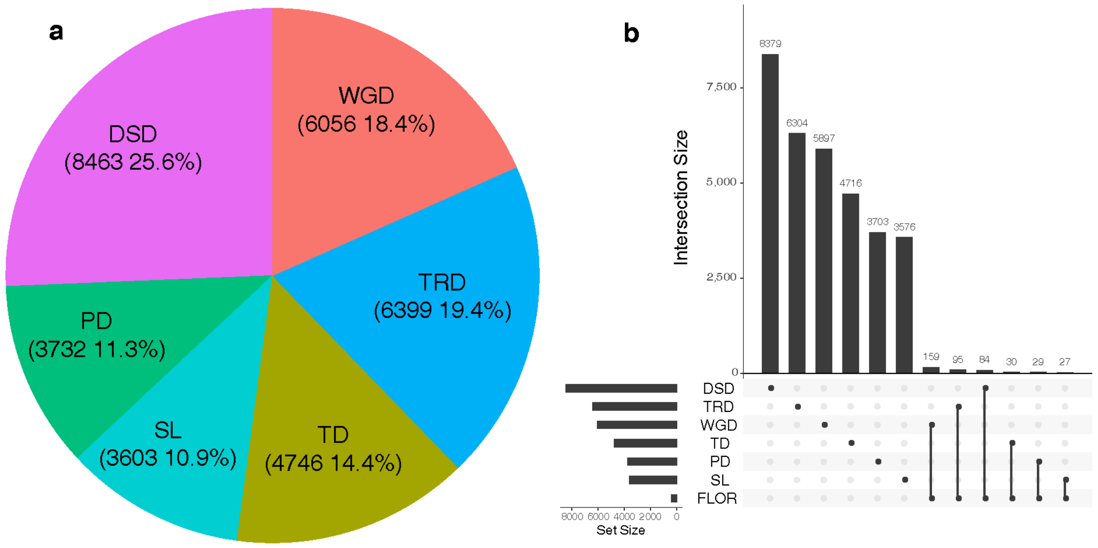

Supplementary Fig. 13. The proportions and UpSet plot of singletons, duplicates, and flowering time genes. a: The proportions of singletons and five modes of gene duplications; **b**: gene UpSet plot of singletons, duplicates, and flowering time genes. WGD: whole-genome duplication, TD: tandem duplication, PD: proximal duplication, TRD: transposed duplication, DSD: dispersed duplication, SL: singletons; FLOR: flowering time genes predicted using FLOR_ID database. Source data are provided as a Source Data file.

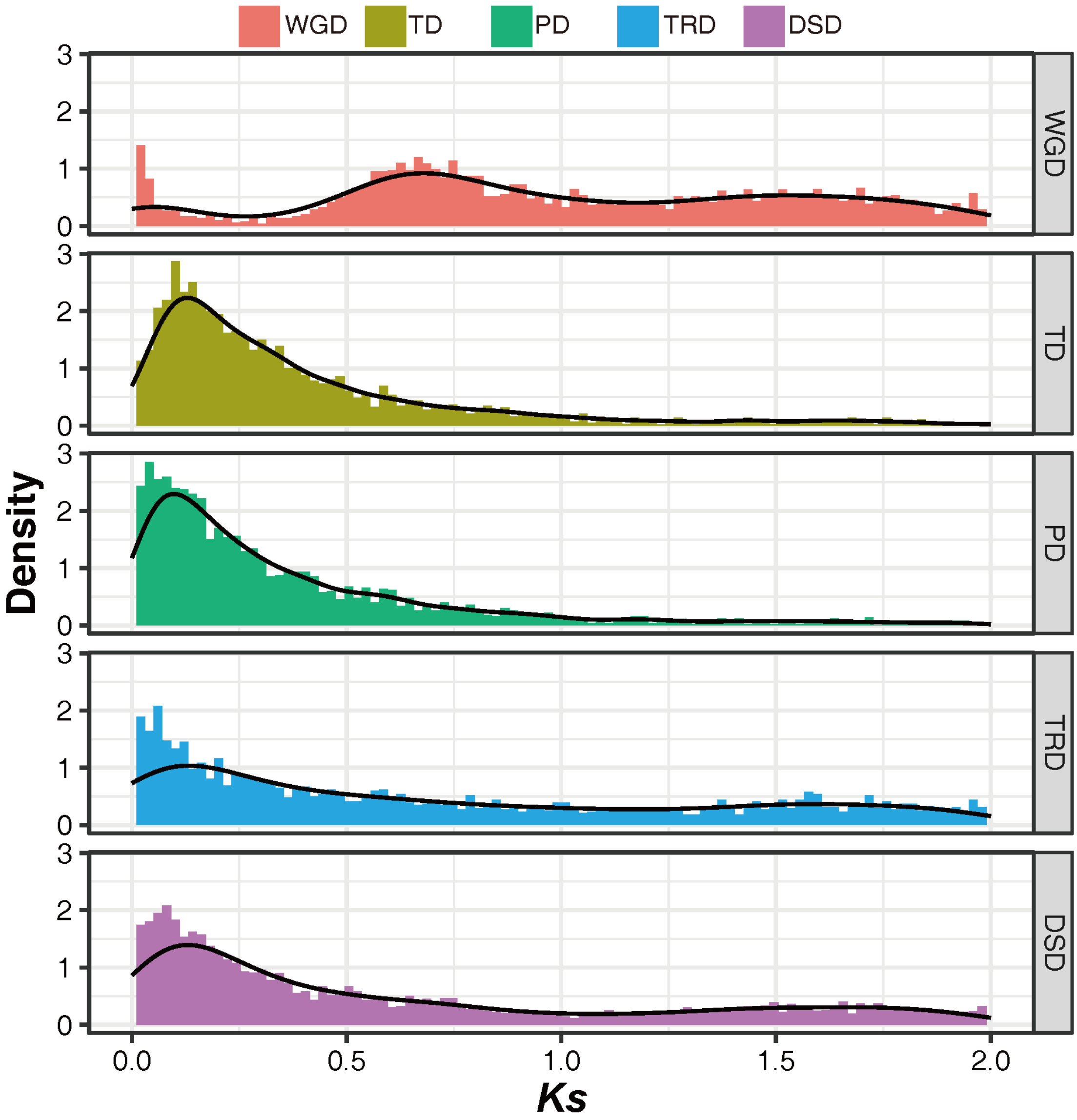

Supplementary Fig. 14. *K*s distribution of the five modes of gene duplications. WGD: whole-genome duplication, TD: tandem duplication, PD: proximal duplication, TRD: transposed duplication, DSD: dispersed duplication. Source data are provided as a Source Data file.

a

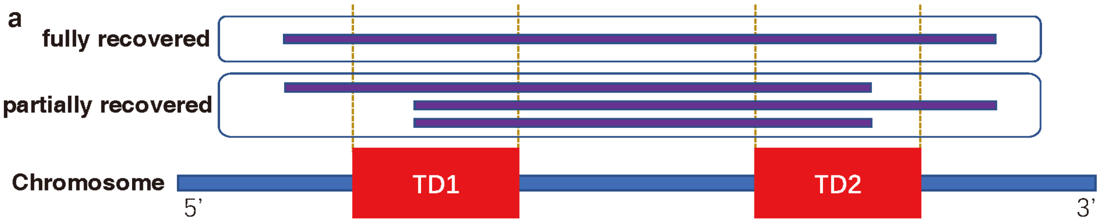

b

Whole genome	fully recovered	1830 (70.82%)
Whole genome	partially recovered	434 (16.80%)
TF	fully recovered	93 (75.61%)
TF	partially recovered	14 (11.38%)
Anthocyanin/flavonol	fully recovered	15 (53.57%)
Anthocyanin/flavonol	partially recovered	7 (25.00%)

Supplementary Fig. 15. Verification of tandem gene duplicates with long-reads mapping. a: a visualization of different types of verification of tandem gene duplicates. blue line is representing a linear genomic region conveying a pair of gene duplicates. red squares labeled with "TD1" and "TD2" are the genic regions of a pair of two tandem gene duplicates. pink lines represent the long-reads mapped to the duplicated region; **b**: a summary table showing all pairs of tandem gene duplicates.

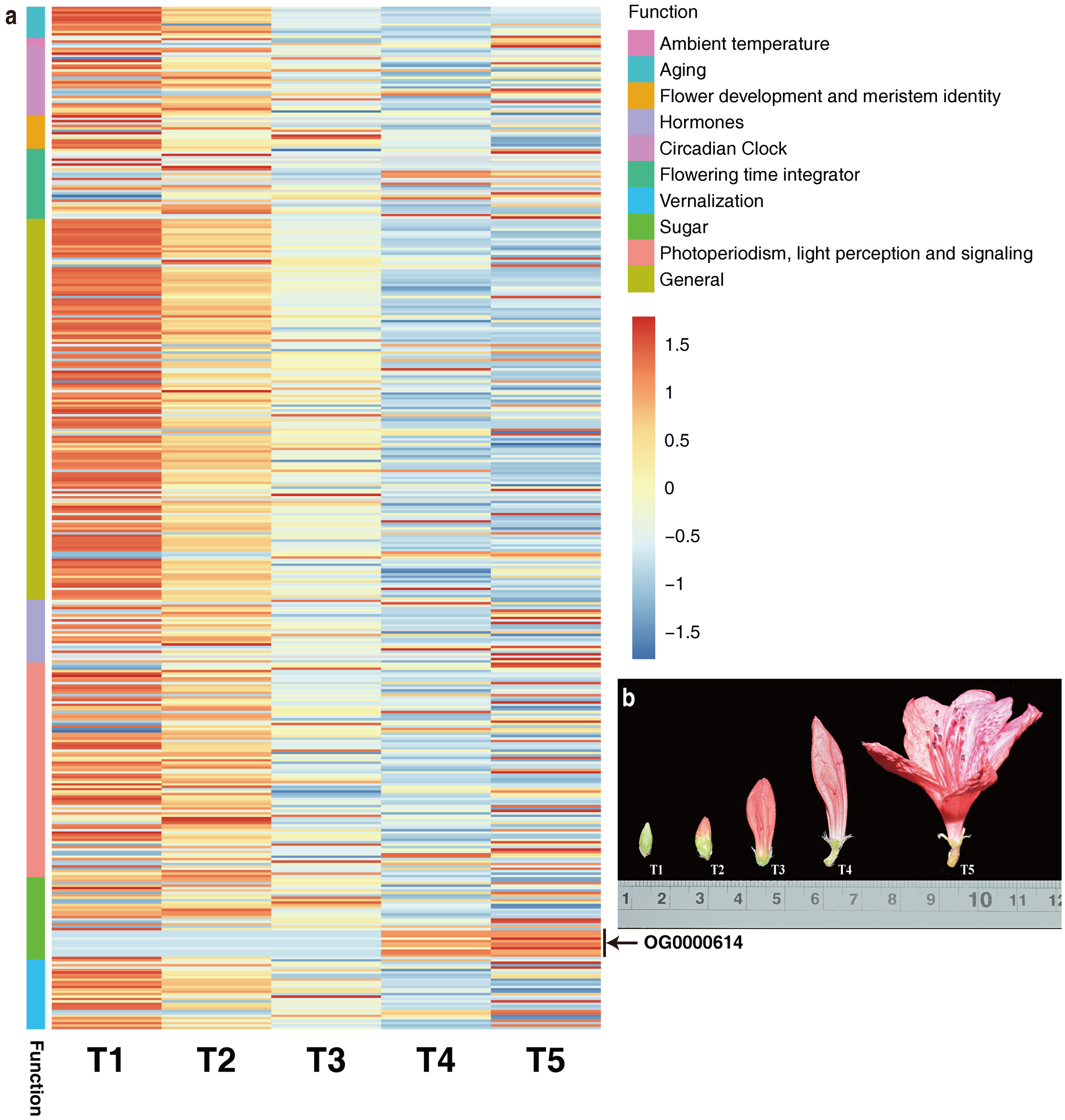

Supplementary Fig. 16. Time-ordered expression of the flowering time genes. a: heatmap; **b**: five stages of flower development in *Rhododendron simsii*. Source data are provided as a Source Data file.

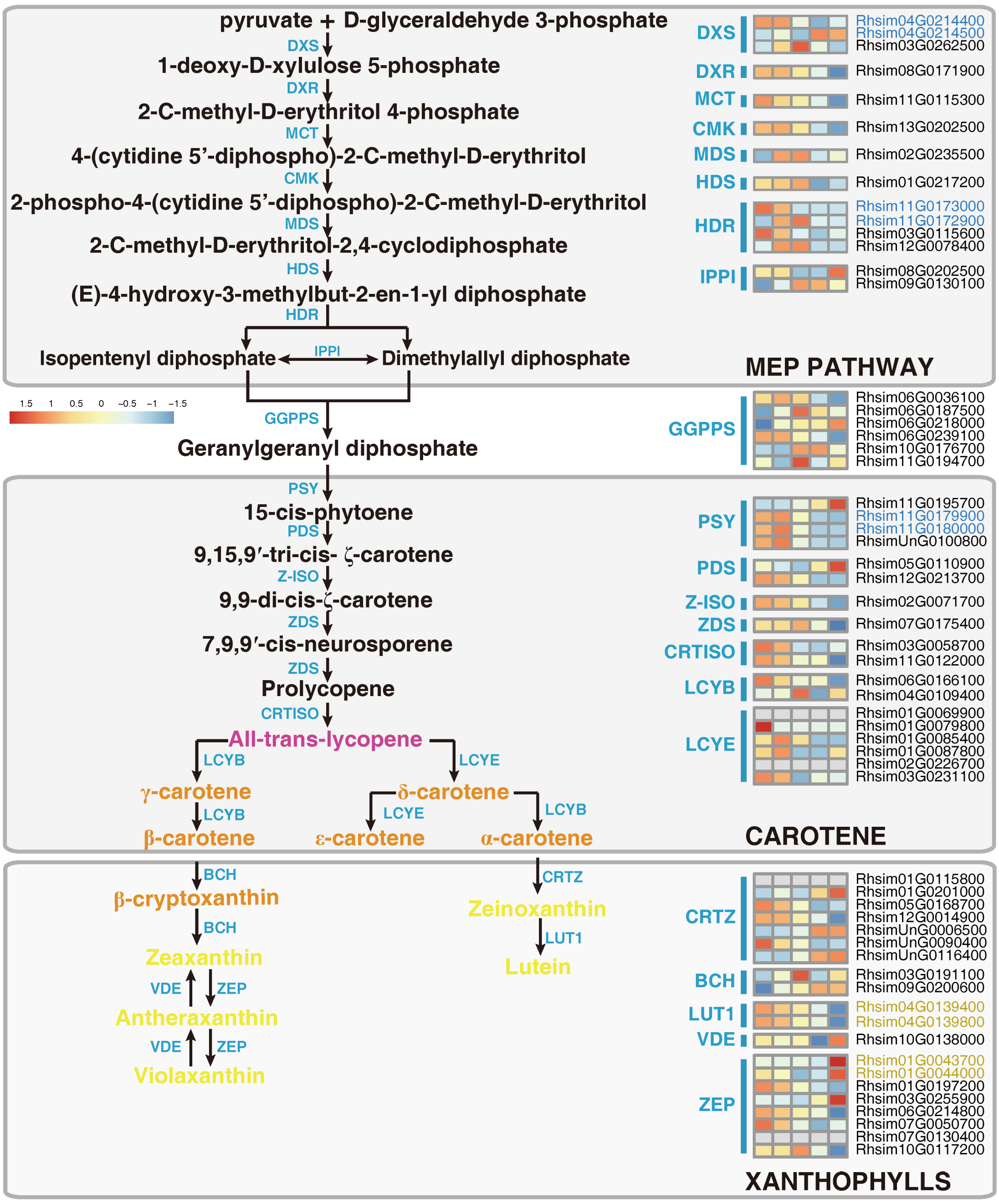

Supplementary Fig. 17. The metabolic pathway and time-ordered gene regulation of carotenoids. Gene expression profile (in normalized TPMs) at different time points of flowering (here T1-T5, from left to right in each heatmap panel) are presented in the heatmap alongside gene names. MEP: 2-C-methyl-D-erythritol 4-phosphate; DXS: 1-deoxy-D-xylulose-5-phosphate synthase (EC 2.2.1.7); DXR: 1-Deoxy-D-xylulose-5-phosphate reductoisomerase (EC 1.1.1.267); MCT: 2-C-methyl-D-erythritol 4-phosphate cytidylyltransferase (EC 2.7.7.60); CMK: 4-(Cytidine 5'-diphospho)-2-C-methyl-D-erythritol kinase (EC 2.7.1.148); MDS: 2-C-methyl-D-erythritol 2,4-cyclodiphosphate synthase (EC 4.6.1.12); HDS: 4-Hydroxy-3-methylbut-2-enyl-diphosphate synthase (EC 1.17.7.1); HDR: 4-Hydroxy-3-methylbut-2-enyl diphosphate reductase (EC 1.17.7.4); IPPI: isopentenyl-pyrophosphate isomerase (EC 5.3.3.2); GGPPS: geranylgeranyl pyrophosphate synthase (EC 2.5.1.29); PSY: phytoene synthase (EC 2.5.1.32); PDS: phytoene desaturase (EC 1.3.5.5); Z-ISO: zeta-carotene isomerase (EC 5.2.1.12); ZDS: zeta-carotene desaturase (EC 1.3.5.6); CRTISO: carotene isomerase (EC 5.2.1.13); LCYB: lycopene beta-cyclase (EC 5.5.1.19); LCYE: lycopene δ-cyclase (EC 5.5.1.18); CRTZ: beta-ring hydroxylase (EC 1.14.99.-); BCH: beta-carotene hydroxylase (EC 1.14.13.129); LUT1: carotene epsilon-monooxygenase (EC 1.14.99.45); VDE: violaxanthin de-epoxidase (EC 1.10.99.3); ZEP: zeaxanthin epoxidase (EC 1.14.13.90). Source data are provided as a Source Data file.

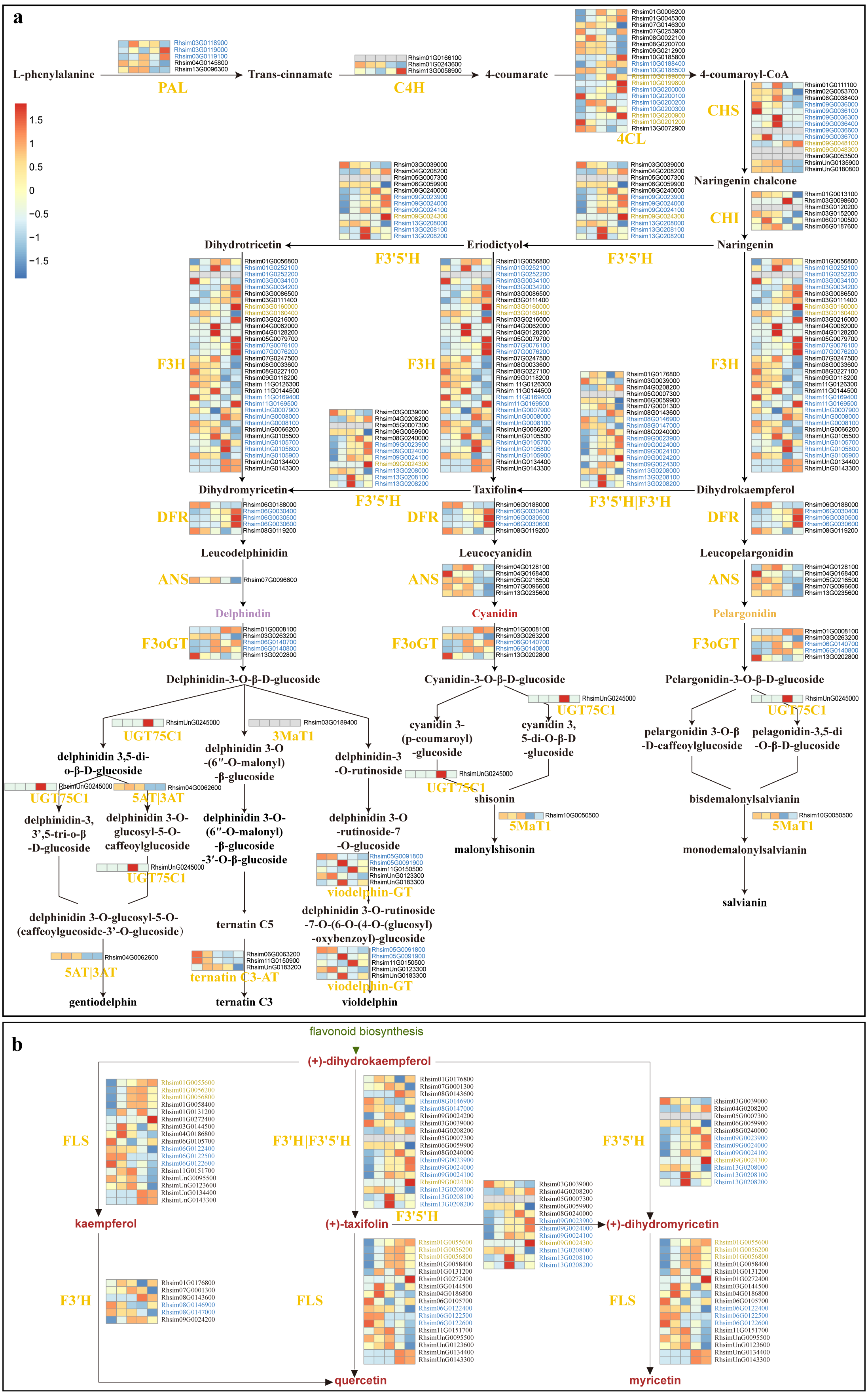

Supplementary Fig. 18. The metabolic pathway and time-ordered gene regulation of two flavonoids: anthocyanin and flavonol. **a**: anthocyanin; **b**: flavonol. Gene expression profile (in normalized TPMs) in different time points of flowering (here T1-T5, from left to right in each heatmap panel) are presented in heatmap alongside gene names. Source data are provided as a Source Data file.

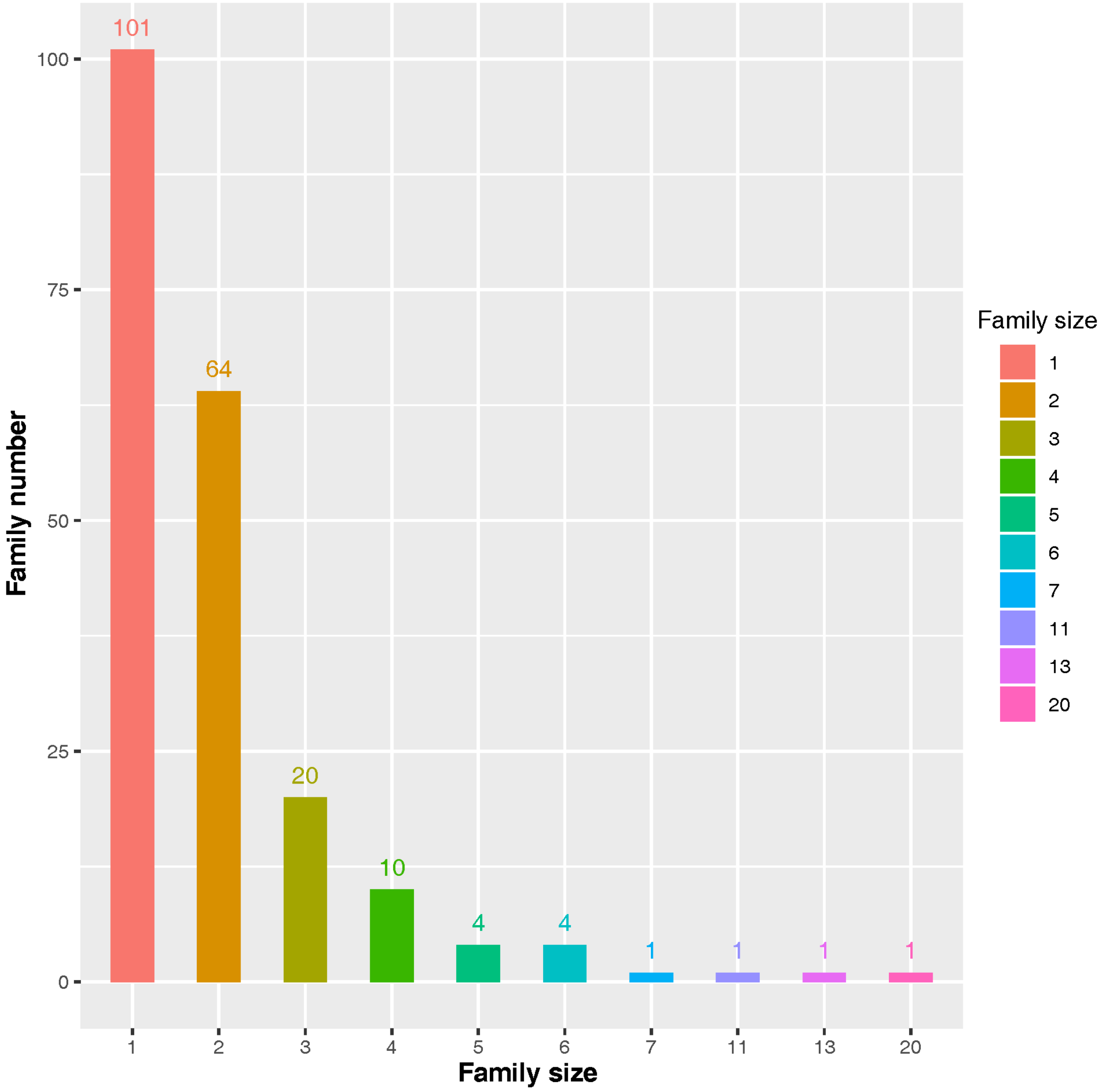

Supplementary Fig. 19. The family numbers and sizes for flowering-time control genes.

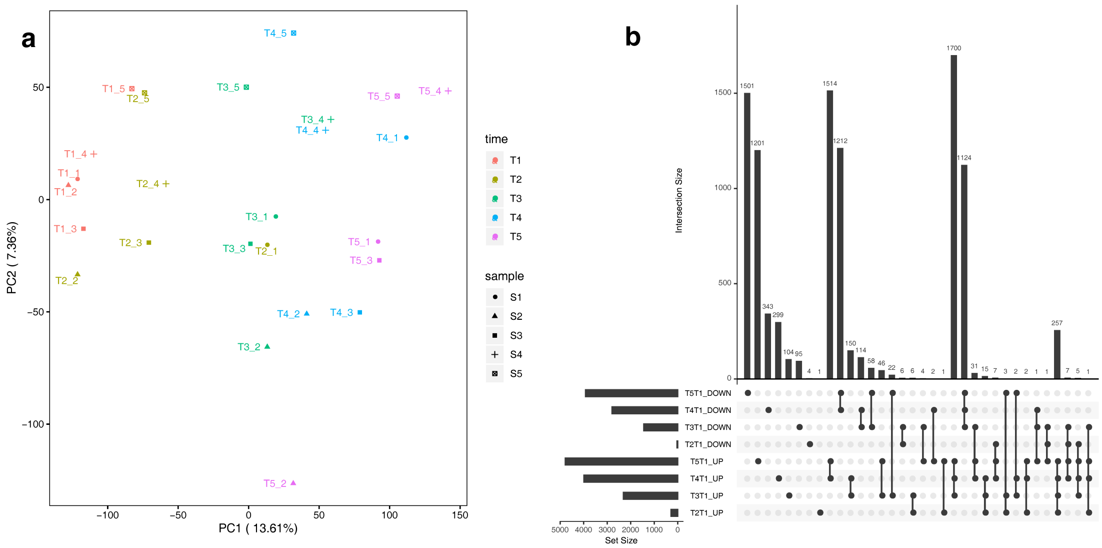

Supplementary Fig. 20. PCA (principal component analysis) and gene differential expression analysis at five developmental stages. a: PCA; **b**: the upset plot of up-regulated and down-regulated genes between T1 and the remaining developmental stages.

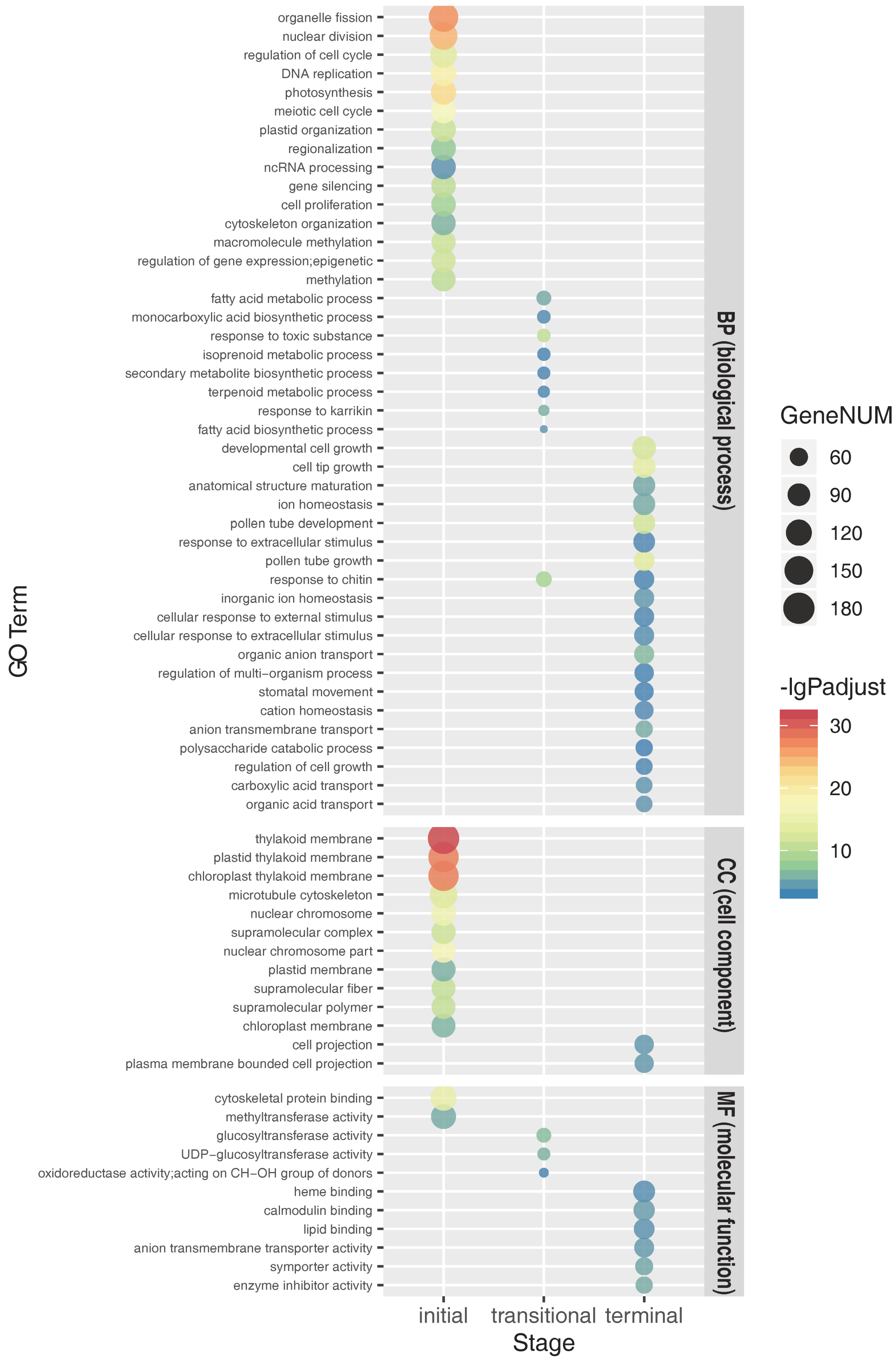

Supplementary Fig. 21. Overrepresented GO functions for co-expressed genes at each level. GO terms (biological process, molecular function, or cellular compartment) were kept with the adjusted *p*-values < 0.001 and the node numbers greater than 100 at the initial stage, 30 at the transitional stage, or 50 at the terminal stage. All genes of the whole genome annotated with GO terms as background. The resulting *p*-values were corrected for multiple comparisons using the method of Benjamini and Hochberg. '*P* adjust' is the Benjamini and Hochberg false discovery rate (FDR) adjusted *p* value. Source data are provided as a Source Data file.

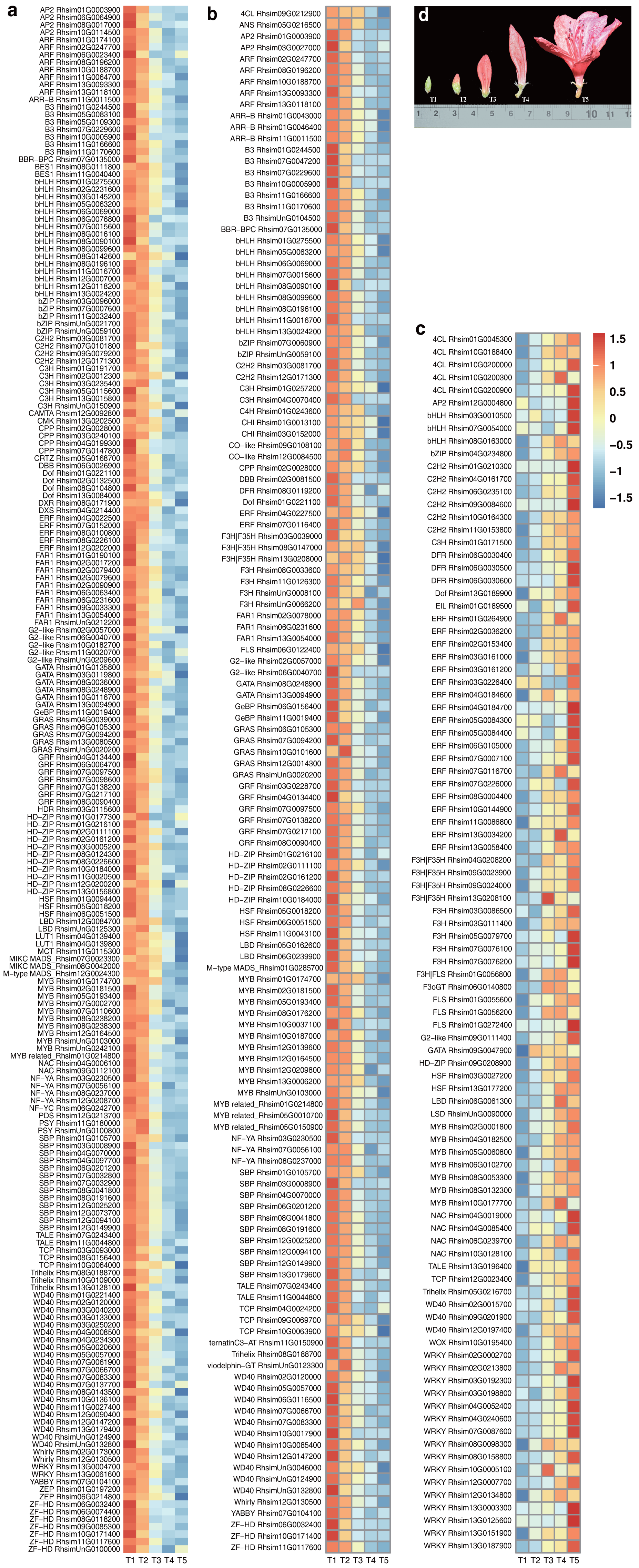

Supplementary Fig. 22. Gene expression of enzymatic genes and related transcription factors related to carotenoid and anthocyanin/flavonol biosynthesis pathways. a: carotenoid biosynthesis at the initial stage; **b**: anthocyanin/flavonol biosynthesis at the initial stage; **c**: anthocyanin/flavonol biosynthesis at the terminal stage; **d**: five stages of flower development in *Rhododendron simsii*. Gene expression profile (in normalized TPMs) at different time points of flowering (here T1-T5, from left to right in each heatmap panel) are presented in heatmap alongside the gene names.

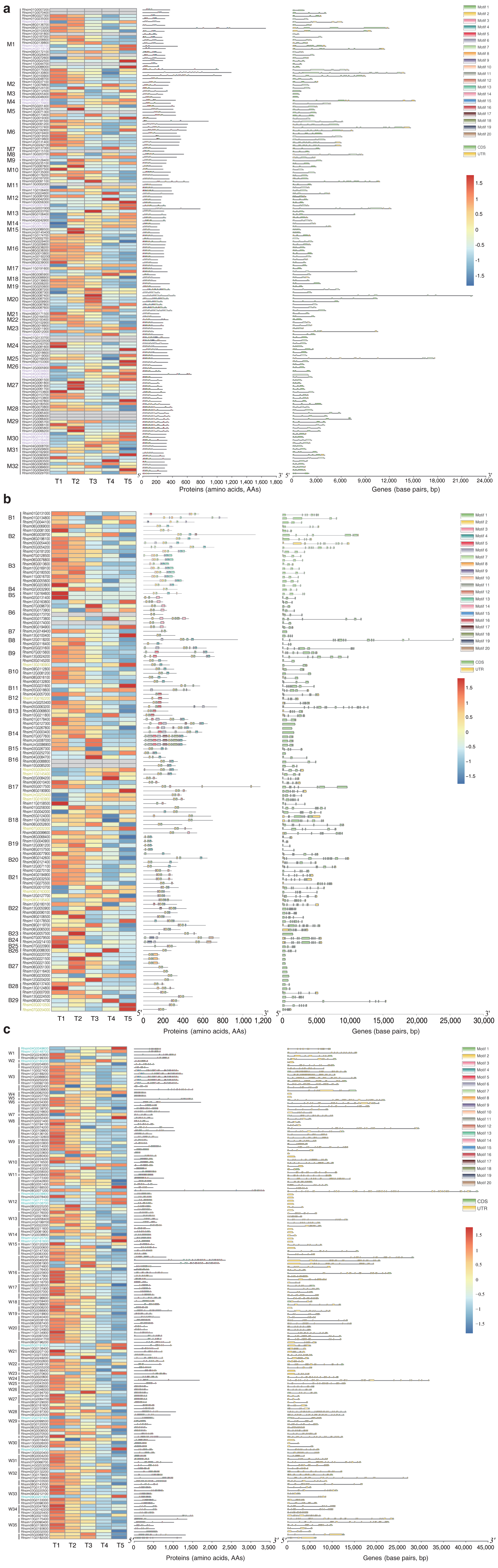
a
b
c
T1
T2
T3
T4
T5
Proteins (amino acids, AAs)
Genes (base pairs, bp)
CDS
UTR

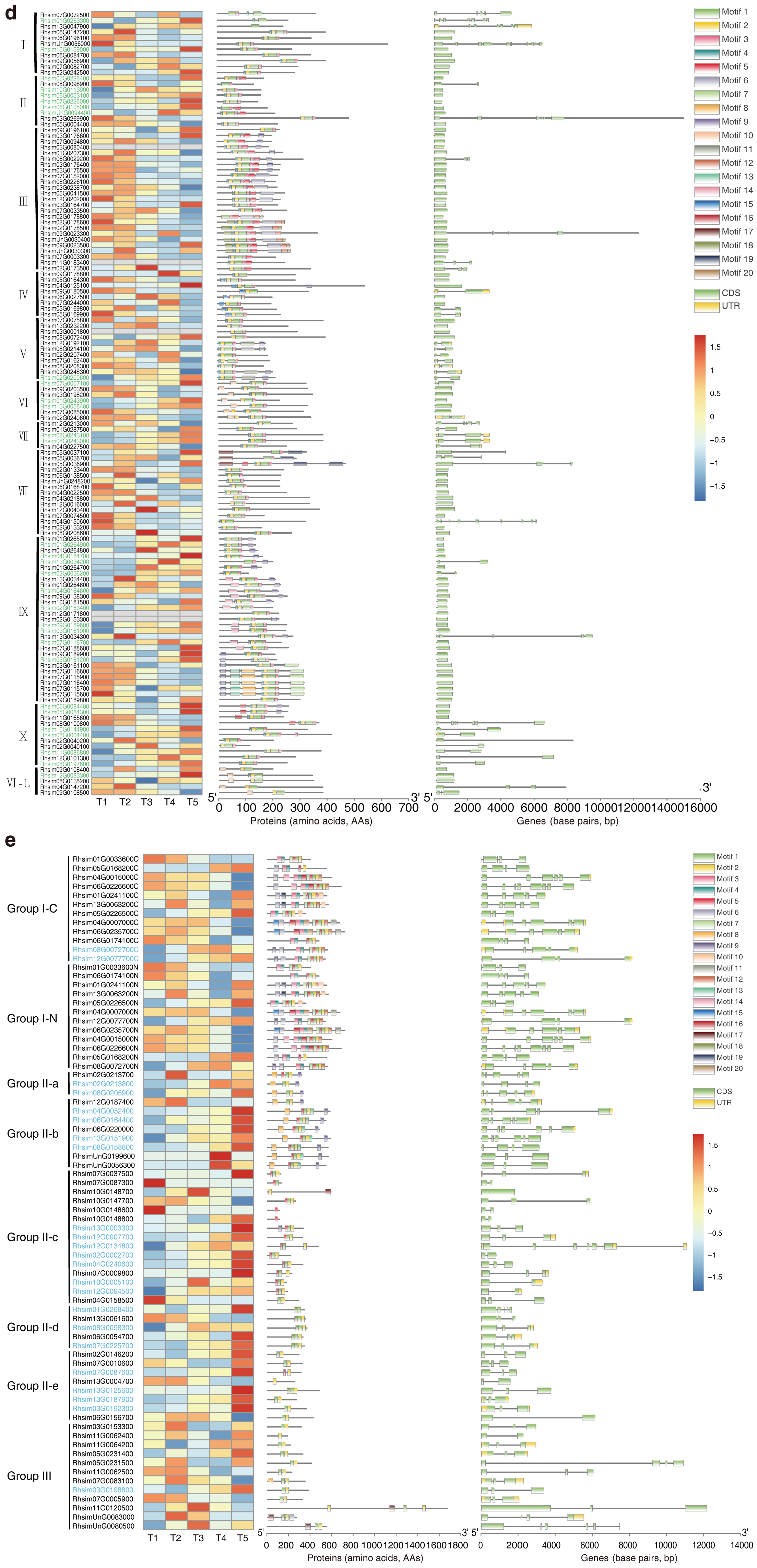

Supplementary Fig. 23. The conserved motifs, gene structure and expression for *MYB*, basic helix-loop-helix (*bHLH*), *WD40*, *ERF*, and *WRKY* transcription factors. The colored gene names (purple, thallite, blue, wathet blue, and green) show genes that are identified as direct regulators of anthocyanins/flavonols biosynthesis genes in L6, L7, and L8, respectively. Groups, gene names, gene expressions, conserved motifs, and gene structures are showed from left to right. **a**: *MYB*; **b**: *bHLH*; **c**: *WD40*; **d**: *ERF*; **e**: *WRKY*. Source data are provided as a Source Data file.

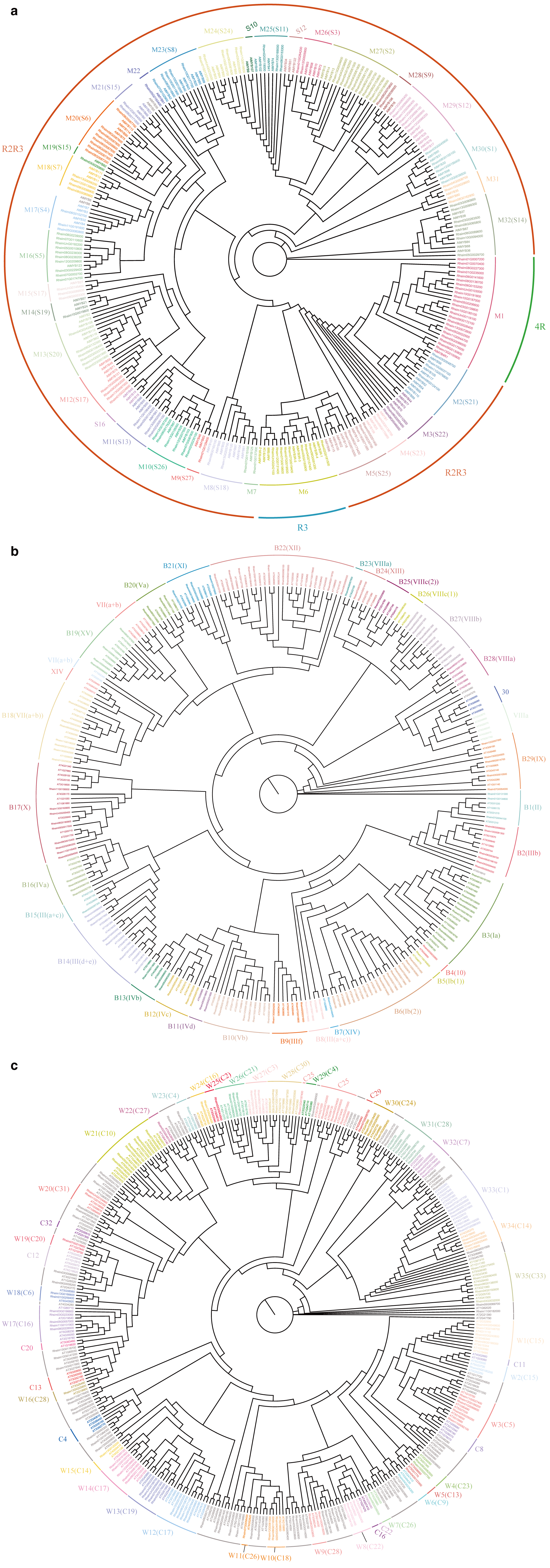
a
R2R3
M24(S24)
S10
M25(S11)
S12
M26(S3)
M27(S2)
M28(S9)
M29(S12)
M30(S1)
M31
M32(S14)
M1
4R
M2(S21)
M3(S22)
M4(S23)
M5(S25)
R2R3
M6
M7
M8(S18)
M9(S27)
M10(S26)
M11(S13)
S16
M12(S17)
M13(S20)
M14(S19)
M15(S17)
M16(S5)
M17(S4)
M18(S7)
M19(S15)
M20(S6)
M21(S15)
M22
M23(S8)
R3
b
B22(XII)
B23(VIIIa)
B24(XIII)
B25(VIIIc(2))
B26(VIIIc(1))
B27(VIIIb)
B28(VIIIa)
30
VIIIa
B29(IX)
B1(II)
B2(IIIb)
B3(Ia)
B4(10)
B5(Ib(1))
B6(Ib(2))
B7(XIV)
B8(III(a+c))
B9(IIIf)
B10(Vb)
B11(IVd)
B12(IVc)
B13(IVb)
B14(III(d+e))
B15(III(a+c))
B16(IVa)
B17(X)
B18(VII(a+b))
XIV
VII(a+b)
B19(XV)
VII(a+b)
B20(Va)
B21(XI)
c
W23(C4)
W24(C16)
W25(C2)
W26(C21)
W27(C3)
W28(C30)
C25
W29(C4)
C25
C29
W30(C24)
W31(C28)
W32(C7)
W33(C1)
W34(C14)
W35(C33)
W1(C15)
C11
W2(C15)
W3(C5)
C8
W4(C23)
W5(C13)
W6(C9)
W7(C26)
C22
C16
W8(C22)
W9(C28)
W10(C18)
W11(C26)
W12(C17)
W13(C19)
W14(C17)
W15(C14)
C4
W16(C28)
C13
C20
W17(C16)
W18(C6)
C12
W19(C20)
C32
W20(C31)
W21(C10)
W22(C27)

d

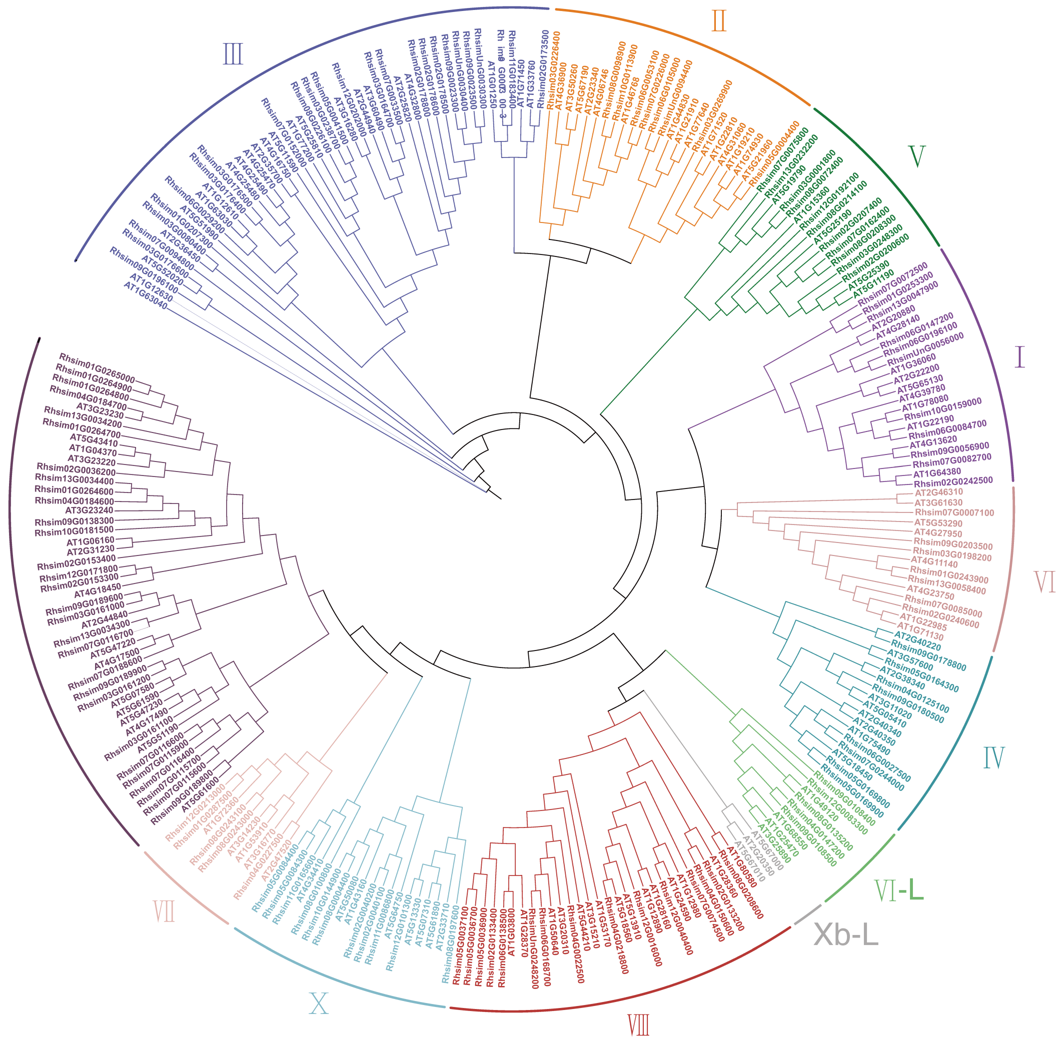

e

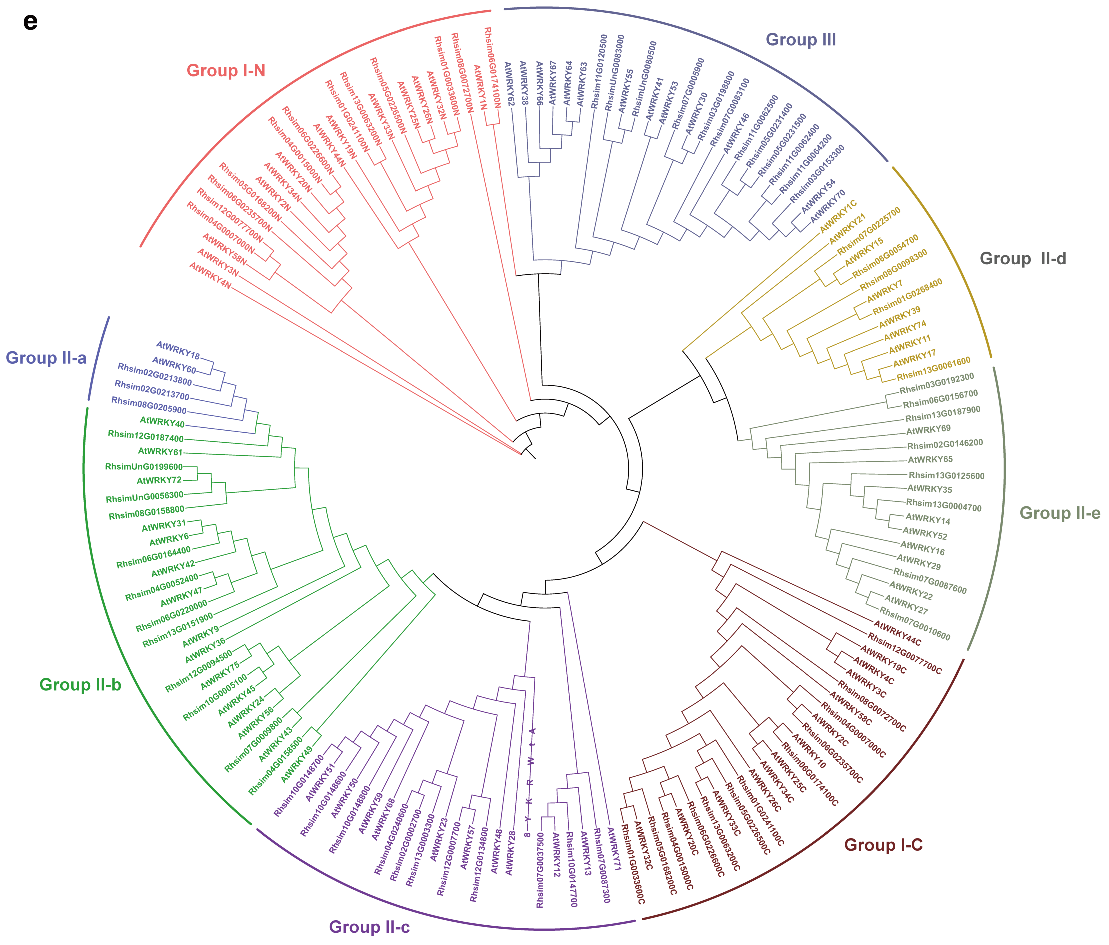

Supplementary Fig. 24. Maximum likelihood (ML) trees of *MYB*, basic helix-loop-helix (*bHLH*), *WD40*, *ERF*, and *WRKY* transcription factors. **a**: *MYB*; **b**: *bHLH*; **c**: *WD40*; **d**: *ERF*; **e**: *WRKY*. *Arabidopsis thaliana* are used as reference for classification of different TF families. Groups in parentheses belong to *Arabidopsis thaliana* and groups outside parentheses belong to *Rhododendron simsii*. Source data are provided as a Source Data file.

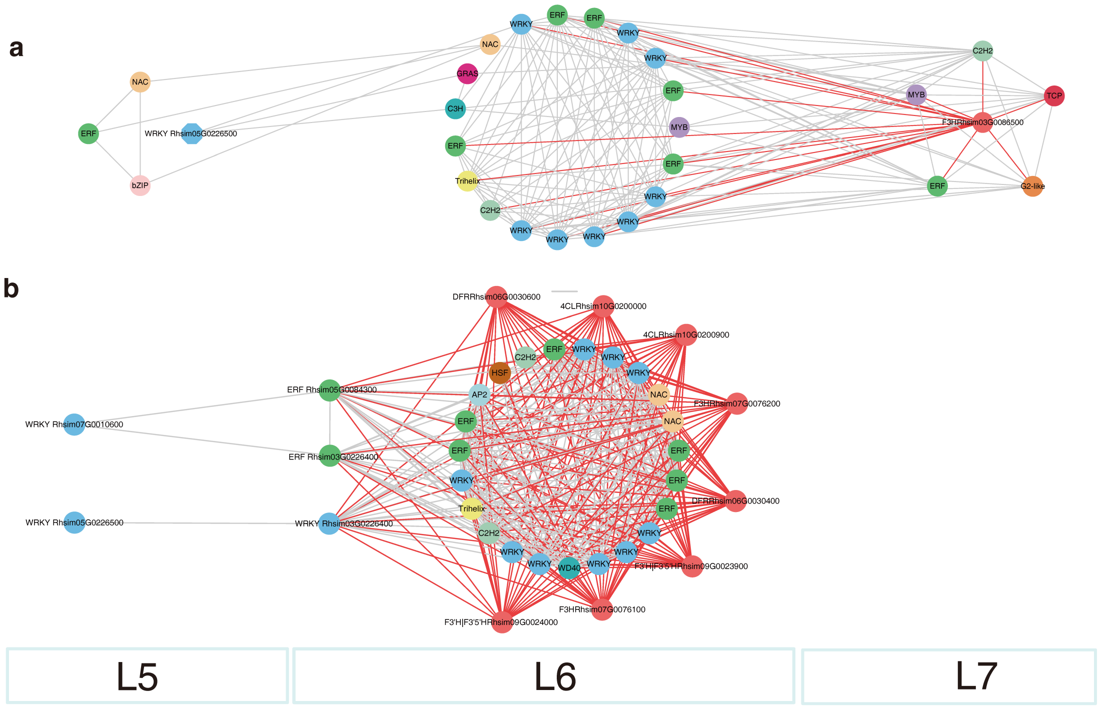

Supplementary Fig. 25. The reconstructed ordered co-expression pathways of nine key enzymatic genes for anthocyanin and carotenoid biosynthesis pathway at the terminal stage. a: *F3H* (*Rhsim03G0086500*); **b**: *DFR* (*Rhsim06G0030400, Rhsim06G0030600*), *F3'H|F3'5'H* (*Rhsim09G0024000, Rhsim09G0023900*), *4CL* (*Rhsim10G0200900, Rhsim10G0200000*), *F3H* (*Rhsim07G0076100, Rhsim07G0076200*).

Supplementary Tables

Supplementary Table 1. *Solanum pimpinellifolium* and *Rhododendron simsii* genome sizes estimated by flow cytometry.

Species	Sample	Genome size (Mb)	Average genome size (Mb)
Solanum pimpinellifolium	fanqie	739	
Rhododendron simsii	Rh_4-1	404.6	414.62
Rhododendron simsii	Rh_4-2	424.63	414.62

Supplementary Table 2. Statistics of the different versions of genome assembly.

Versions of assembly	Strategy	Assemblied genome size (Gb)	Sequence number	N50	L50	Max. length	Gene completeness (%)
**v0.1**	SMARTDENOVO	600	3886	340 Kb	476	2.1 Mb	NA
**v0.2**	WTDBG	543	4805	380 Kb	399	2.5 Mb	NA
**v0.3**	Corrected by CANU + SMARTDENOVO	530	2840	370 Kb	385	2.5 Mb	93.10%
**v0.4**	Corrected by CANU + WTDBG	512	3708	520 Kb	288	3.4 Mb	90.60%
**v0.5**	Corrected by CANU (80x) + SMARTDENOVO	550	3025	390 Kb	397	2.7 Mb	NA
**v0.6**	Corrected by CANU (80x) + WTDBG	509	4119	458 Kb	300	4.4 Mb	NA
**v0.7**	CANU	940	14201	255 Kb	832	3.5 Mb	NA
**v0.8**	FALCON-Phase	700	2207	525 Kb	389	2.9 Mb	NA
**v1.0**	v0.3(q) + v0.4(r) + quickmerge + pilon	538	1763	950 Kb	157	12.1 Mb	92.20%
**v1.1**	v1.0 + Hi-C + gapclose + pilon×5	529	911/552	2.2 Mb/36 Mb*	66/7	11.9 Mb/48 Mb*	93.70%

N50: shortest sequence length at 50% of the genome; L50: smallest number of contigs whose length sum produces N50. NA: data not available; * statistics for contigs/scaffolds. Gene completeness was generated by assessment with 1,440 single copy orthologs from the BUSCO embryophyta_odb9 database.

Supplementary Table 3. Statistics of the genome quality for the last assembly.

Type	Number	Length (bp)	Percent
**chromsome-scale scaffold**	13	481,946,564	91.17%
**mitochondrial**	1	802,707	0.15%
**chloroplast**	1	152,214	0.03%
**contig-scale scaffold**	537	45,735,662	8.65%
**genome size**	NA	528,637,147	NA
**genome size without N**	NA	528,609,592	NA
**GCcontent**	NA	NA	38.91%
**A**	161,476,558	NA	NA
**T**	161,419,382	NA	NA
**G**	102,903,121	NA	NA
**C**	102,810,530	NA	NA
**N**	27,555	NA	NA
**Others**	1	NA	NA
**contig**	911	NA	NA
**contig Max**	NA	11,877,617	NA
**contig Mean**	NA	580,252	NA
**contig N10**	NA	6,595,922	NA
**contig N50**	NA	2,234,511	NA
**contig N90**	NA	326,356	NA
**contig Min**	NA	10,897	NA
**contig Median**	NA	96,860	NA
**contig L10**	7	NA	NA
**contig L50**	66	NA	NA
**contig L90**	283	NA	NA
**scaffold**	552	NA	NA
**scaffold Max**	NA	47,608,546	NA
**scaffold Mean**	NA	957,675	NA
**scaffold N10**	NA	45,065,412	NA
**scaffold N50**	NA	36,350,743	NA
**scaffold N90**	NA	30,661,963	NA
**scaffold Min**	NA	10,897	NA
**scaffold Median**	NA	58,946	NA
**scaffold L10**	2	NA	NA
**scaffold L50**	7	NA	NA
**scaffold L90**	13	NA	NA
**gap**	359	NA	NA
**gap Max**	NA	188	NA
**gap Mean**	NA	76	NA
**gap Min**	NA	2	NA
**gap Median**	NA	82	NA

NA, data not available.

Supplementary Table 4. Summary of BUSCO evaluation for genome assembly and gene prediction.

	Genome assembly	Genome assembly	Protein-coding genes	Protein-coding genes
	**BUSCO groups**	**Percentage**	**BUSCO groups**	**Percentage**
**Complete BUSCOs**	1,349	93.68%	1346	93.47%
**Complete and single-copy BUSCOs**	1,223	84.93%	1195	82.99%
**Complete and duplicated BUSCOs**	126	8.75%	151	10.49%
**Fragmented BUSCOs**	15	1.04%	19	1.32%
**Missing BUSCOs**	76	5.28%	75	5.21%
**Total BUSCO groups searched**	1,440	100.00%	1440	100.00%

Supplementary Table 5. Summary of annotated genes.

Class	Feature
**Gene number**	34,170
**Protein coding gene number**	32,999
**Transcript number**	34,170
**Transcript number (AED<0.5)**	29,773
**Average gene region length (bp)**	5089.2
**Average transcript length (bp)**	1416.3
**Average coding sequence length (bp)**	1288.7
**Average exons per transcript**	5
**Average exon length (bp)**	259.7
**Average intron length (bp)**	403.1

AED: Annotation Edit Distance; gene region (including 5’, 3’ UTRs, exons and introns).

Supplementary Table 6. Summary of annotated RNA genes.

Source	Gene Category	Gene Number
**maker**	mRNA	32,999
**Rfam**	ncRNA	625
**Rfam**	miRNA	221
**Rfam**	tRNA	16
**Rfam**	snoRNA	158
**RNAmmer-1.2**	rRNA	64
**RNAmmer-1.2**	28S rRNA	8
**RNAmmer-1.2**	18S rRNA	6
**RNAmmer-1.2**	5S rRNA	50
**tRNAScan-SE**	tRNA	482

Supplementary Table 7. Summary of functional annotation of predicted genes.

	Databases	Count	Percentage
**Total genes**		32,999	100%
**Blat**	eggNOG	27,098	82.10%
**Blat**	GO	25,038	75.90%
**Blat**	KO	11,506	34.90%
**Blat**	NR	28,273	85.70%
**Blat**	Pfam	24,301	73.60%
**Blat**	Swiss_Prot	19,079	57.80%
**Blat**	TrEMBL	28,016	84.90%
**Blat**	Unannotated	4,681	14.20%
**interProScan**	TIGRFAM	2,677	8.11%
**interProScan**	PANTHER	28,517	86.42%
**interProScan**	CDD	9,057	27.45%
**interProScan**	Coils	5,076	15.38%
**interProScan**	Gene3D	20,617	62.48%
**interProScan**	GO	18,986	57.54%
**interProScan**	Hamap	699	2.12%
**interProScan**	IPR	26,342	79.83%
**interProScan**	KEGG	2,020	6.12%
**interProScan**	MetaCyc	1,448	4.39%
**interProScan**	MobiDBLite	13,872	42.04%
**interProScan**	Pfam	24,232	73.43%
**interProScan**	Phobius	11,513	34.89%
**interProScan**	PIRSF	1,558	4.72%
**interProScan**	PRINTS	3,651	11.06%
**interProScan**	ProDom	369	1.12%
**interProScan**	ProSitePatterns	5,084	15.41%
**interProScan**	ProSiteProfiles	10,722	32.49%
**interProScan**	Reactome	3,102	9.40%
**interProScan**	SFLD	203	0.62%
**interProScan**	SignalP_EUK	3,003	9.10%
**interProScan**	SignalP_GRAM_NEGATIVE	1,095	3.32%
**interProScan**	SignalP_GRAM_POSITIVE	2,289	6.94%
**interProScan**	SMART	8,768	26.57%
**interProScan**	SUPERFAMILY	19,170	58.09%
**interProScan**	TMHMM	7,483	22.68%
**interProScan**	Unannotated	1,175	3.56%

Supplementary Table 8. Summary of intra- and inter- genomic collinearity.

	Intragenome	Intragenome	Intragenome	Intergenome-*V. vinifera*	Intergenome-*V. vinifera*	Intergenome-*V. vinifera*
**Species**	**BlockNumber**	**CollinearGenesNumber**	**GenePairsNumber**	**BlockNumber**	**CollinearGenesNumber**	**GenePairsNumber**
Actinidia chinensis	1,075	22,566	19,905	890	28,528	18,016
Camellia sinensis	31	415	208	526	8,274	4,33
Camptotheca acuminata	608	13,541	10,406	691	26,174	15,100
Rhododendron delavayi	110	1,707	910	738	16,384	8,822
Rhododendron simsii	289	6,213	3,729	603	20,726	12,002
Rhododendron williamsianum	216	5,158	3,218	465	18,959	10,190
Vitis vinifera	147	3,730	2,152			

Supplementary Table 9. Summary of the annotated TEs in the genome assembly.

**Class**	**Family**	**Number**	**Length (bp)**	**Percent (%)**	**Mean_length (bp)**
**LTR**		126,553	89,927,654	17.0112249036	710.59
**LTR**	Cassandra	696	132,879	0.025136145039	190.92
**LTR**	Caulimovirus	2,925	2,311,206	0.437200831065	790.16
**LTR**	Copia	40,330	21,171,170	4.00485855376	524.95
**LTR**	DIRS	109	24,826	0.004696226918	227.76
**LTR**	ERV1	3,733	2,599,653	0.491765101025	696.40
**LTR**	ERVK	139	80,923	0.015307853498	582.18
**LTR**	Gypsy	75,182	62,902,254	11.8989470106	836.67
**LINE**		28,651	11,605,857	2.19542971315	405.08
**LINE**	I-Jockey	2,095	512,417	0.096931705028	244.59
**LINE**	L1	17,201	7,286,672	1.37838818958	423.62
**LINE**	L1-Tx1	1,180	1,173,529	0.221991399329	994.52
**LINE**	L2	2,532	962,890	0.182145731806	380.29
**LINE**	Penelope	162	28,053	0.005306664535	173.17
**LINE**	RTE-BovB	5,301	1,611,923	0.304920493981	304.08
**LINE**	Tad1	180	30,373	0.005745528889	168.74
**SINE**		27,097	3,833,175	0.725105116383	141.46
**SINE**	ID	141	13,221	0.00250095932	93.77
**SINE**	tRNA	24,175	3,360,780	0.635744199792	139.02
**SINE**	tRNA-7SL	319	57,219	0.010823870461	179.37
**SINE**	tRNA-RTE	1,031	112,902	0.021357182453	109.51
**DNA**		139,105	33,638,200	6.36319263429	241.82
**DNA**	CMC-EnSpm	9,848	2,815,863	0.532664610495	285.93
**DNA**	Crypton-H	270	60,404	0.011426363119	223.72
**DNA**	Crypton-V	433	80,609	0.015248455477	186.16
**DNA**	Dada	2,688	668,311	0.126421497958	248.63
**DNA**	Ginger	397	67,959	0.012855509754	171.18
**DNA**	Kolobok-T2	2,632	301,529	0.057038935252	114.56
**DNA**	MULE-MuDR	18,832	4,711,581	0.891269375741	250.19
**DNA**	MuLE-MuDR	4,103	2,657,623	0.502731034904	647.73
**DNA**	PIF-Harbinger	4,628	1,124,814	0.212776193724	243.05
**DNA**	PIF-Spy	3,532	1,720,259	0.325413945986	487.05
**DNA**	PiggyBac	116	76,137	0.014402506602	656.35
**DNA**	Sola-2	562	136,222	0.025768525873	242.39
**DNA**	Sola-3	427	46,147	0.008729428164	108.07
**DNA**	TcMar-Stowaway	7,995	1,260,009	0.238350446455	157.60
**DNA**	Zisupton	924	72,321	0.013680650406	78.27
**DNA**	hAT	838	183,753	0.034759759325	219.28
**DNA**	hAT-Ac	30,696	7,031,280	1.33007679084	229.06

**DNA**	hAT-Tag1	10,158	1,964,277	0.371573774402	193.37
**DNA**	hAT-Tip100	11,678	2,371,823	0.448667486472	203.10
**RC**		2,119	1,405,702	0.265910560387	663.38
**RC**	Helitron	2,119	1,405,702	0.265910560387	663.38
**Unknown**		454,663	101,697,067	19.237593797	223.68
**rRNA**		206	27,957	0.00528850463	135.71
**Satellite**		1,368	333,174	0.063025082874	243.55
**Simple_repeat**		154,764	7,479,217	1.41481109348	48.33
**Low_complexity**		19,155	916,225	0.173318315824	47.83
**snRNA**		648	124,540	0.023558692518	192.19
**Total**		954,329	250,988,768	47.48	263.00

LTR: Long Terminal Repeat retrotransposons; LINE: Long Interspersed Nuclear Element, a category of non-LTR (long terminal repeat) retroelements; SINE: Short Interspersed Nuclear Element, a category of non-autonomous and non-coding retroelements (TEs); RC: Rolling-circle transposons.

Supplementary Table 10. Comparison of the number of original and filtered intact LTR-RT, solo-LTR, and truncated LTR TEs in *R. simsii* and the 16 reference plant species.

Species	Intact LTR-RT (*I*)	Cluster number	Solo-LTR (*S*)	Truncated LTR (*T*)	*S*+*T*	*I*+*S*+*T*	Filtered scaffold length (kb)	Filtered *I*	Filtered *S*	Filtered *T*	Filtered *S*/*I*	Filtered *T*/*I*	Filtered (*S*+*T*)/*I*	LTR-RT (*S*/*I*>=3)
Actinidia chinensis	2,486	1,128	5,895	7,787	13,682	16,168	0	2,486	5,895	7,787	2.37	3.13	5.5	26.204
Arabidopsis thaliana	299	205	275	450	725	1,024	0	299	275	450	0.92	1.51	2.42	17.28
Camellia sinensis	17,200	4,611	155,520	196,734	352,254	369,454	305	16,363	136,540	186,258	8.34	11.38	19.73	33.956
Camptotheca acuminata	1,660	484	4,224	7,086	11,310	12,970	750	1,254	2,549	4,481	2.03	3.57	5.61	17.368
Coffea canephora	2,055	765	11,938	9,291	21,229	23,284	0	2,055	11,938	9,291	5.81	4.52	10.33	41.85
Daucus carota	1,296	566	4,882	8,383	13,265	14,561	1,460	1,017	3,437	5,770	3.38	5.67	9.05	33.928
Eucommia ulmoides	5,063	1,930	30,009	32,787	62,796	67,859	560	4,331	18,166	27,068	4.19	6.25	10.44	28.194
Helianthus annuus	12,532	2,347	48,529	249,023	297,552	310,084	90	12,424	47,278	245,398	3.81	19.75	23.56	24.657
Lactuca sativa	16,549	2,695	87,760	198,906	286,666	303,215	400	14,781	75,775	175,200	5.13	11.85	16.98	37.276
Primula vulgaris	1,658	456	9,088	6,167	15,255	16,913	120	1,446	6,512	5,122	4.5	3.54	8.05	30.281
Rhododendron delavayi	1,703	592	13,181	12,134	25,315	27,018	180	1,535	10,675	10,948	6.95	7.13	14.09	45.301
Rhododendron simsii	2,128	605	5,934	6,515	12,449	14,577	900	1,910	5,254	5,563	2.75	2.91	5.66	30.303
Rhododendron williamsianum	NA	NA	NA	NA	NA	NA	NA	NA	NA	NA	NA	NA	NA	NA
Sesamum indicum	981	577	1,792	2,093	3,885	4,866	1,625	798	1,344	1,446	1.68	1.81	3.5	16.34
Solanum lycopersicum	6,826	1,895	15,686	27,135	42,821	49,647	0	6,826	15,686	27,135	2.3	3.98	6.27	21.156
Vaccinium corymbosum	6,478	1,020	26,605	20,833	47,438	53,916	1,665	6,053	24,607	18,958	4.07	3.13	7.2	39.09
Vitis vinifera	4,116	915	7,734	15,339	23,073	27,189	0	4,116	7,734	15,339	1.88	3.73	5.61	20.411

Supplementary Table 11. Superfamilies within the *Gypsy* and *Copia* LTR-RT classes of TEs.

**Super families**	**Clade ID**	**Count**	**Total length (bp)**	**Average length (bp)**	**Proportion of genome (%)**	**Overlap with gene (2kb)**
Gypsy	Athila	230	2,821,461	12,267	0.5395%	67 (29.13%)
Gypsy	Tekay	230	2,147,507	9,337	0.4106%	42 (18.26%)
Gypsy	Ogre	135	1,795,509	13,300	0.3433%	37 (27.41%)
Gypsy	Retand	97	1,120,198	11,548	0.2142%	26 (26.80%)
Gypsy	CRM	118	698,782	5,922	0.1336%	68 (57.63%)
Gypsy	Reina	12	75,620	6,302	0.0145%	11 (91.67%)
Gypsy	Galadriel	3	21,775	7,258	0.0042%	2 (66.67%)
Gypsy		825	8,680,852	10,522	1.66%	
Copia	Ale	512	2,916,647	5,697	0.5577%	270 (52.73%)
Copia	Tork	250	1,377,556	5,510	0.2634%	62 (24.80%)
Copia	TAR	187	1,256,580	6,720	0.2403%	27 (14.44%)
Copia	Angela	153	1,204,901	7,875	0.2304%	16 (10.46%)
Copia	Ikeros	77	570,632	7,411	0.1091%	40 (51.95%)
Copia	Ivana	73	425,089	5,823	0.0813%	36 (49.32%)
Copia	SIRE	44	395,228	8,982	0.0756%	37 (84.09%)
Copia	Bianca	6	45,037	7,506	0.0086%	3 (50.00%)
Copia	Alesia	1	4,774	4,774	0.0009%	0 (0.00%)
Copia		1303	8,196,444	6,290	1.5672%	

Supplementary Table 12. Summary of gene family analyses.

Species	Number of genes	Number of genes in orthogroups	Number of unassigned genes	Percentage of genes in orthogroups	Percentage of unassigned genes	Number of orthogroups containing species	Percentage of orthogroups containing species	Number of species-specific orthogroups	Number of genes in species-specific orthogroups	Percentage of genes in species-specific orthogroups
Actinidia chinensis	33,044	31,771	1,273	96	4	13,547	60	12	37	0
Arabidopsis thaliana	27,416	22,932	4,484	84	16	12,249	55	70	729	3
Camellia sinensis	33,932	30,478	3,454	90	10	13,740	61	39	105	0
Camptotheca acuminata	31,825	27,275	4,550	86	14	13,935	62	23	70	0
Coffea canephora	25,574	22,223	3,351	87	13	13,265	59	37	200	1
Daucus carota	32,113	26,256	5,857	82	18	12,863	57	100	650	2
Eucommia ulmoides	26,722	22,994	3,728	86	14	13,295	59	31	105	0
Helianthus annuus	58,229	42,829	15,400	74	26	13,899	62	283	1,715	3
Lactuca sativa	38,910	30,352	8,558	78	22	13,868	62	81	488	1
Primula vulgaris	24,599	20,234	4,365	82	18	11,439	51	64	343	1
Rhododendron delavayi	32,938	29,430	3,508	89	11	15,297	68	18	64	0
Rhododendron simsii	32,999	30,889	2,110	94	6	14,768	66	12	40	0
Rhododendron williamsianum	21,419	20,634	785	96	4	13,070	58	3	6	0
Sesamum indicum	27,148	22,748	4,400	84	16	12,392	55	46	466	2
Solanum lycopersicum	34,725	27,205	7,520	78	22	13,568	60	60	394	1
Vaccinium corymbosum	128,559	94,280	34,279	73	27	16,230	72	349	1,655	1
Vitis vinifera	26,346	21,143	5,203	80	20	12,880	57	19	75	0

Unique groups and genes, single-copy and duplicated groups and genes are summarized for *R. simsii* and the 16 reference plant species.

Supplementary Table 13. Genomic data used for phylogenomic and gene family analyses.

Species	Version	Genes	Genome size (Mb)	Scaffold N50 (Mb)	References (DOI)
Actinidia chinensis	Red5_PS1_1.69	33,044	553.8	18.9	10.1186/s12864-018-4656-3
Arabidopsis thaliana	TAIR10	27,416	135	24	10.1093/nar/gkr1090
Camellia sinensis	-	33,932	3100	1.4	10.1073/pnas.1719622115
Camptotheca acuminata	v2.4	40,332	403	1.7	10.1093/gigascience/gix065
Coffea canephora	-	25,574	569	1.26	10.1126/science.1255274
Daucus carota	v2.0	32,113	421	12.7	10.1038/ng.3565
Eucommia ulmoides	-	26,722	1200	1.9	10.1016/j.molp.2017.11.014
Helianthus annuus	HanXRQr1.0	73,728	3000	178	10.1038/nature22380
Lactuca sativa	Lsat_Salinas_v7	43,794	2800	1.7	10.1038/ncomms14953
Primula vulgaris	2018	24,599	411.1	0.294	10.1038/s41598-018-36304-4
Rhododendron delavayi	-	32,938	695.1	0.637	10.1093/gigascience/gix076
Rhododendron williamsianum	R.will10	21,419	368.4	0.219	10.1093/gbe/evz245
Sesamum indicum	v1.0	27,148	274	2.1	10.1186/gb-2014-15-2-r39
Solanum lycopersicum	ITAG2.4	34,725	823	66	10.1038/nature11119
Vaccinium corymbosum	v1.0	128,559	1800	36.9	10.1093/gigascience/giz012
Vitis vinifera	Genoscope.12X	26,346	486	23	10.1038/nature06148

Origins, download links, assembly versions, genome properties, and references of 16 reference genomes are shown, * – data not available.

Supplementary Table 14. Proportion of tandem (TD) or proximal (PD) duplicated genes of flower coloration in different species.

		Rhododendron simsii	***Rhododendron williamsianum***	***Rhododendron delavayi***	***Actinidia chinensis***	***Primula vulgaris***	***Arabidopsis thaliana***
**flower coloration**	ALL	197	113	174	185	144	113
**flower coloration**	TD	51 (25.89%)*	21 (18.58%)	0 (0%)	16 (8.65%)	18 (12.5%)	14 (12.39%)
**flower coloration**	PD	24 (12.18%)*	4 (3.54%)	0 (0%)	2 (1.08%)	12 (8.33%)	7 (6.19%)
**flower coloration**	TD/PD	75 (38.07%)*	25 (22.12%)	0 (0.00%)	18 (9.73%)	30 (20.83%)	21 (18.58%)

All, all identified genes; TD, tandem duplicated genes; PD, proximal duplicated genes; TD/PD, tandem or proximal duplicated genes. * $P < 0.05$; Significance was tested with two-sided t-test under confidence level of 0.95.

Supplementary Table 15. Quantity and proportion of genes in tandem (TD) or proximal (PD) gene clusters related to carotenoid and anthocyanin/flavonol biosynthesis pathways.

		Rhododendron simsii	***Rhododendron williamsianum***	***Rhododendron delavayi***	***Actinidia chinensis***	***Primula vulgaris***	***Arabidopsis thaliana***
**carotenoid**	ALL	58	41	57	69	50	50
**carotenoid**	TD/PD	10 (17.24%)	4 (9.76%)	10 (17.54%)	0 (0%)	2 (4%)	5 (10%)
**carotenoid**	TD	6 (10.34%)	4 (9.76%)	4 (7.02%)	0	0	0
**carotenoid**	PD	4 (6.90%)	0	6 (10.53%)	0	2 (4%)	5 (10%)
**anthocyanin/flavonol**	ALL	139	72	117	116	94	63
**anthocyanin/flavonol**	TD/PD	59 (42.45%)	25 (34.72%)	30 (25.64%)	23 (19.83%)	22 (23.4%)	18 (28.57%)
**anthocyanin/flavonol**	TD	48 (34.53%)	21 (29.17%)	17 (14.53%)	21 (18.1%)	14 (14.89%)	16 (25.4%)
**anthocyanin/flavonol**	PD	11 (7.91%)	4 (5.56%)	13 (11.11%)	2 (1.72%)	8 (8.51%)	2 (3.17%)
**carotenoid/anthocyanin/flavonol**	ALL	197	113	174	185	144	113
**carotenoid/anthocyanin/flavonol**	TD/PD	69 (35.03%)	29 (25.66%)	40 (22.99%)	23 (12.43%)	24 (16.67%)	23 (20.35%)
**carotenoid/anthocyanin/flavonol**	TD	54 (27.41%)	25 (22.12%)	21 (12.07%)	21 (11.35%)	14 (9.72%)	16 (14.16%)
**carotenoid/anthocyanin/flavonol**	PD	15 (7.61%)	4 (3.54%)	19 (10.92%)	2 (1.08%)	10 (6.94%)	7 (6.19%)

All, all identified genes; TD, tandem duplicated genes; PD, proximal duplicated genes; TD/PD, tandem or proximal duplicated genes.

Supplementary Table 16. The core enzymatic genes for carotenoid, anthocyanin/flavonol biosynthesis in the initial and terminal stages.

Stages	Biosynthesis	Enzyme	Gene
**Initial**	anthocyanin/flavonol	viodelphin-GT	RhsimUnG0123300
**Initial**	anthocyanin/flavonol	ternatinC3-AT	Rhsim11G0150900
**Initial**	anthocyanin/flavonol	FLS	Rhsim06G0122400
**Initial**	anthocyanin/flavonol	F3'H|F3'5'H	Rhsim13G0208000; Rhsim08G0147000; Rhsim03G0039000
**Initial**	anthocyanin/flavonol	F3H	RhsimUnG0008100; Rhsim08G0033600; RhsimUnG0066200; Rhsim11G0126300
**Initial**	anthocyanin/flavonol	DFR	Rhsim08G0119200
**Initial**	anthocyanin/flavonol	CHI	Rhsim01G0013100; Rhsim03G0152000
**Initial**	anthocyanin/flavonol	C4H	Rhsim01G0243600
**Initial**	anthocyanin/flavonol	ANS	Rhsim05G0216500
**Initial**	anthocyanin/flavonol	4CL	Rhsim09G0212900
**Initial**	carotenoid	CMK	Rhsim13G0202500
**Initial**	carotenoid	CRTZ	Rhsim05G0168700
**Initial**	carotenoid	DXR	Rhsim08G0171900
**Initial**	carotenoid	DXS	Rhsim04G0214400
**Initial**	carotenoid	HDR	Rhsim03G0115600
**Initial**	carotenoid	LUT1	Rhsim04G0139800; Rhsim04G0139400
**Initial**	carotenoid	MCT	Rhsim11G0115300
**Initial**	carotenoid	PDS	Rhsim12G0213700
**Initial**	carotenoid	PSY	RhsimUnG0100800; Rhsim11G0180000
**Initial**	carotenoid	ZEP	Rhsim01G0197200; Rhsim06G0214800
**Terminal**	anthocyanin/flavonol	F3oGT	Rhsim06G0140800
**Terminal**	anthocyanin/flavonol	F3H|FLS	Rhsim01G0056800
**Terminal**	anthocyanin/flavonol	F3'H|F3'5'H	Rhsim04G0208200; Rhsim09G0023900; Rhsim13G0208100; Rhsim09G0024000
**Terminal**	anthocyanin/flavonol	F3H	Rhsim03G0111400; Rhsim05G0079700; Rhsim03G0086500; Rhsim07G0076200; Rhsim07G0076100
**Terminal**	anthocyanin/flavonol	DFR	Rhsim06G0030400; Rhsim06G0030500; Rhsim06G0030600
**Terminal**	anthocyanin/flavonol	4CL	Rhsim01G0045300; Rhsim10G0188400; Rhsim10G0200000; Rhsim10G0200300; Rhsim10G0200900
**Terminal**	anthocyanin/flavonol	FLS	Rhsim01G0272400; Rhsim01G0056200; Rhsim01G0055600
**Terminal**	carotenoid	BCH	Rhsim09G0200600
**Terminal**	carotenoid	CRTZ	RhsimUnG0116400
**Terminal**	carotenoid	IPPI	Rhsim09G0130100
**Terminal**	carotenoid	ZEP	Rhsim03G0255900; Rhsim01G0044000

Supplementary Table 17. The predicted direct regulators of 14 core enzymatic genes for anthocyanin/flavonol biosynthesis including 31 TFs: nine *bHLH*, 11 *WD40* and 11 *MYB*.

TF	genes
**bHLH**	Rhsim05G0063200
**bHLH**	Rhsim08G0090100
**bHLH**	Rhsim11G0016700
**bHLH**	Rhsim08G0099600
**bHLH**	Rhsim01G0275500
**bHLH**	Rhsim06G0069000
**bHLH**	Rhsim13G0024200
**bHLH**	Rhsim07G0015600
**bHLH**	Rhsim08G0196100
**MYB**	Rhsim08G0176200
**MYB**	Rhsim02G0181500
**MYB**	Rhsim01G0174700
**MYB**	Rhsim10G0037100
**MYB**	Rhsim12G0139600
**MYB**	Rhsim13G0006200
**MYB**	RhsimUnG0103000
**MYB**	Rhsim05G0193400
**MYB**	Rhsim12G0209800
**MYB**	Rhsim12G0164500
**MYB**	Rhsim10G0187000
**WD40**	RhsimUnG0132800
**WD40**	RhsimUnG0046000
**WD40**	RhsimUnG0124900
**WD40**	Rhsim10G0085400
**WD40**	Rhsim07G0083300
**WD40**	Rhsim07G0066700
**WD40**	Rhsim12G0147200
**WD40**	Rhsim02G0120000
**WD40**	Rhsim06G0116500
**WD40**	Rhsim05G0057000
**WD40**	Rhsim10G0017900

Supplementary Note 1. Estimation of genome size, heterozygosity, and repeat content

All PacBio reads were filtered and corrected using Canu (version 1.7)[3]. Next, Jellyfish (version 2)[4] was employed to count *K*-mers. Finally, GenomeScope (version 1.0)[5] and GCE (version 1.0.0)[6] were used to estimate genome size, repeat content, and the level of heterozygosity.

We also collected fresh leaves and estimated the genome size using an Elite flow cytometer (BD FACSCalibur, USA) with 'CyStain PI Absolute P kit' (Sysmex, Germany). We employed the reagent kit for nuclei extraction and DNA staining of nuclear DNA following the protocol. Two replications were performed and *Solanum pimpinellifolium* (739 Mb) was used as the calibration standard. The average genome size for *R. simsii* was estimated to be 414.62 Mb (**Supplementary Table 1**).

Comparing to the result from flow cytometry, we identified 73,532,815,926 *K*-mers with *K*-mer size 17, and the heterozygous peak was at 70. The genome size was estimated to be ~525 Mb (**Supplementary Fig. 2**). The final PacBio cleaned data corresponded to the coverage of approximately 100-fold. Repeat and error frequencies were estimated to be 55.09% and 0.45%, respectively. The estimated heterozygosity was at ~1.78%.

Supplementary Note 2. Plant material

Tissue samples for genome sequencing were obtained from a 20-year-old shrub from Jingshan, Hubei Province, China. This shrub was transplanted in the Botanical Garden of Institute of Botany, Chinese Academy of Science, Beijing, China. Leaf tissue was used for genome library preparation, and samples from three different tissues (flowers, young leaves and young stems) were used for RNA sequencing to enable genome annotation. Fresh tissues were immediately transferred into liquid nitrogen and stored at -80 °C until DNA and RNA extractions commenced.

To unravel the gene regulatory network underpinning flower coloring, samples of corolla at five flower developmental stages were collected from five field individuals (**Fig. 4a**). Fresh tissues were stored in RNAlater (Ambion, Life Technologies, Austin, TX, USA) and then conserved at -80 °C after flash frozen with liquid nitrogen.

Supplementary Note 3. Genome sequencing

PacBio SMRT sequencing and Illumina short-read sequencing

Total DNA was isolated and extracted from the leaves using the DNeasy Plant Mini Kit (QIAGEN, Inc.) and then purified using the Mobio PowerClean Pro DNA Clean-Up Kit (MO BIO Laboratories, Inc.) to obtain high molecular weight and high-quality genomic DNA. Subsequently, we assessed the quality of DNA before PacBio and Illumina library preparation.

For PacBio SMRT (single-molecule real-time) sequencing, sheared and concentrated genomic DNA was applied to size selection by the Blue Pippin system (Sage Sciences). Then, sequencing libraries with 20-kb DNA inserts were constructed according to PacBios amplicon library protocol and sequenced on a PacBio RSII platform using P6-C4 chemistry (6 SMRT cells). A total of 6.5 million PacBio long reads were generated, yielding 51.15 Gb (roughly 100× coverage of the assembled genome) of 6,489,286 single-molecule sequencing reads with an average read length of 7,705 bp.

For Illumina sequencing, 150 bp paired-end (PE) PCR-free libraries were prepared using the NEBNext Ultra II DNA Library Prep Kit for sequencing with an Illumina

HiSeq X Ten platform. Short reads were processed with fastp (version0.19.3)[1] to remove adapter sequences, leading and trailing bases with a quality score below 20, and reads with an average per-base-quality of 20 over a 4-bp sliding window. Reads <70 nucleotides in length after trimming were removed from further analysis. Finally, we obtained 605.896 million reads. This produced ~91.49 Gb (roughly 170× the assembled genome) of raw sequencing data.

Hi-C library construction and sequencing

Briefly, after the leaves were fixed with formaldehyde and lysed, the cross-linked DNA was digested with the MboI restriction enzyme and then biotinylated at the 5' overhangs. The blunt-end fragments were ligated to form chimeric junctions that were purified, physically sheared, and enriched for biotin-containing fragments. Subsequently, we performed DNA fragment end repair, adaptor ligation, and polymerase chain reaction, and then constructed paired-end sequencing libraries. The Hi-C libraries were constructed following a previously published study[2]. These libraries were then sequenced using the Illumina HiSeq X Ten platform with 150 bp PE reads.

As a result, we obtained 55.68 Gb of raw data (roughly 100× coverage of the assembled genome).

RNA sequencing

Frozen tissues obtained from the three types of tissue (that is stem, leave and flower) and corollas at five developmental stages, with five biological replicates per stage, were ground with a mortar and a pestle. Messenger RNA was isolated using the NEBNext Poly(A) mRNA Magnetic Isolation Module, and the quality was determined by the Agilent 2100 BioAnalyzer. In total, 28 sequencing libraries were constructed using the NEBNext Ultra RNA Library Prep Kit for Illumina.

Finally, 150 bp PE sequencing was performed on an Illumina HiSeq X Ten machine and we obtained a total of 130.679 million raw reads (~ 20 Gb, from the three tissue types) for gene annotation and 402.55 G raw reads (from a total of 25 corolla samples corresponding to the five flower developmental stages) for the gene expression study.

Supplementary Note 4. Genome assembly

Assembly of chloroplast and mitochondrial genomes

Preceding the filtered and corrected PacBio reads, genomic reads were mapped on both organelle genomes of closely related species by minimap2 (version 2.11-r797)[7]; *Vaccinium macrocarpon* (PRJNA236297), *Rhazya stricta* (PRJNA252472), *Hesperelaea palmeri* (PRJNA345035), *Corchorus capsularis* (PRJNA348006), and *Vitis vinifera* (PRJNA33471) for mitochondrial assembly; and *Cymbidium ensifolium* (PRJNA304815), *Diospyros kaki* (PRJNA339092), *Pouteria campechiana* (PRJNA368858), *Diospyros blancoi* (PRJNA368859), and *Vaccinium macrocarpon* (PRJNA182664) for chloroplast assembly. All mapped reads were extracted for the following assemblies. Firstly, we used Canu (version 1.7) and SMARTdenovo (version 1.0.0) (https://github.com/ruanjue/smartdenovo) to generate two primary assemblies. For the chloroplast genome, the assembly from SMARTdenovo was selected for high quality. Similarly, contigs from Canu (-correct -p assembly useGrid=true corOutCoverage=80 minReadLength=5000) were used to assemble the mitochondrial genome using SeqMan (version 11)[8]. Finally, we annotated and illustrated the two organelle genomes using the GeSeq web service (https://chlorobox.mpimp-golm.mpg.de/geseq.html)[9].

Finally, the assembled mitochondrial genome gave a linear scaffold of 802,707 bp, with 45.87% average GC content. A total of 82 protein-coding genes were annotated in the genome, in addition to 60 annotated tRNA and 25 rRNA genes.

Compared to this mitochondrial genome, the 152,214 bp long chloroplast genome is much smaller, with a much lower GC content of 35.74%. Moreover, this assembled chloroplast genome has the quadripartite structure found in most land plant chloroplast genomes, containing 256 genes (including 90 tRNA and 166 protein-coding genes).

See **Supplementary Fig. 5** for detailed annotation of all 423 genes in both organelle genomes.

De novo nuclear genome assembly

The *de novo* genome assembly employed the following three steps: primary assembly, Hi-C scaffolding, and polishing with Overlap-Layout-Consensus approach. Firstly, the primary assembly v0.1 was generated by SMARTdenovo (version 1.0.0) and assembly v0.2 by WTDBG (version 2.1)[10] from raw PacBio long reads. Then, we used the corrected reads from Canu (version 1.7) to prepare another two assemblies: assembly v0.3 by SMARTdenovo and assembly v0.4 by WTDBG. Next, higher quality reads, corrected by Canu (-correct -p assembly useGrid=true corOutCoverage=80 minReadLength=5000), and representing above 80 × coverage, were used to generate assembly v0.5 by SMARTdenovo and assembly v0.6 by WTDBG. In addition, assembly v0.7 was prepared by Canu and assembly v0.8 by FALCON-Phase v0.1.0-beta (https://github.com/WGLab/EnhancedFALCON). After comparison among different primary assemblies on continuity and completeness, assembly v0.3 (reasonably sized assembly, fewest contigs) and assembly v0.4 (highest contig N50) were chosen as optimal for further analysis. After merging assembly v0.3 with assembly v0.4 by quickmerge (version 0.2)[11], the merged assembly was further polished with high quality Illumina reads with one round of pilon (version 1.22) (http://github.com/broadinstitute/pilon) to produce assembly v1.0.

Subsequently, valid Hi-C data were processed together with assembly v1.0 by 3D-DNA pipeline (version 180922) (https://github.com/theaidenlab/3d-dna) to produce primary scaffolds. These scaffolds were roughly spilt by Juicebox (version 1.8) (https://github.com/aidenlab/Juicebox), and each scaffold was processed by 3D-DNA (version 180922). Afterwards, we elaborately optimized the new scaffolds by removing error insert, bound, order, and mis-join. After scaffold adjustment, we merged chromosome-level scaffolds, scattered contigs, and organelle genomes for further gap closing and polishing. Gaps were closed by LR_Gapcloser (version 1.1) (https://github.com/CAFS-bioinformatics/LR_Gapcloser) with raw PacBio long reads and five rounds of pilon polishing with filtered Illumina short reads.

For the polished assembly, we recognized and selectively removed debased contigs, such as redundant contigs (i.e. same region in homologous chromosomes) with Redundans (version 0.13c)[16], or contigs which are less than 5 bp in length, and contigs representing either low coverage below 10-fold or high non-coverage above 60%. Thereafter, the filtered assembly was aligned to the NT database with blastn (version 2.2.28+)[17] (coverage of 90%), and we confirmed that the final assembly v1.1 was not polluted by cross-contamination.

Evaluation of assembly quality

In the final assembly, a chromosome-level genome size of 529 Mb was obtained, consisting of 911 contigs, 522 scaffolds (with contig N50 of 2.2 Mb, scaffold N50 of 36 Mb, longest contig of 11.9 Mb, and longest scaffold of 48 Mb), indicating a good contiguity for our assembly.

The completeness and continuity of our assemblies were assessed in several ways: (1) the reference assembly was judged by a high LTR Assembly Index (LAI) score of 18.10[18]; (2) 93.7% completed genes were found by mapping 1,440 conserved plant orthologous genes to the assembled genome in BUSCO assessment[19] (**Supplementary**

Table 2 and 3); (3) when all clean Illumina reads were mapped to the final assembly, a high sequence coverage of 99.5% and reads mapping rate of 93.3% were obtained with BWA-MEM (https://github.com/lh3/bwa); (4) an even higher sequence coverage of 99.8% was observed for mapping PacBio long reads to the final assembly by minimap2 (version 2.11-r797), and 90.9% of the reads could be mapped. (5) Using RNA-seq data from different tissues (flowers, young leaves and young stems), a total 87.0 % of the sequences could be mapped onto the genome assembly by HiSat2 (version 2.1.0) (https://github.com/infphilo/hisat2).

For assessing the correctness of our assembly, after Illumina reads from genome sequencing mapped to the final assembly, we gained a heterozygosity of ~1.07% and a single base error rate of ~0.0054% based on SNPs identified with SAMtools[22]. Additionally, there was no obvious GC bias in the sequencing data from PacBio SMRT technology, whereas there was in the Illumina sequencing data. The chromatin interactions were shown by Hi-C reads mapped onto the final assembly by Juicer (https://github.com/aidenlab/juicer) (**Supplementary Fig. 4**).

Supplementary Note 5. Genome annotation

Transposable element and other repeat annotation

A *de novo* repeat identification approach was pursued with RepeatModeler (version 1.0.8) (http://www.repeatmasker.org) to identify repeat element boundaries and family relationships in the assembled genome. Subsequently, the outputs from RepeatModeler were used for further characterization of transposable elements (TEs) and other repeats by homology-based methods, including identification with RepeatMasker v4.0.7 (rmblast-2.2.28) (http://www.repeatmasker.org). In sum, 250,988,768 bp (47.5%) was predicted to be TEs and/or repeats in the assembled genome, predominantly known TEs (25.56%) as well as uncharacterized TEs (19.24%), with a smaller number (1.41%) of simple repeats (**Supplementary Fig. 8**). Repeat annotations are provided in **Supplementary Table 9**.

We further examined the classification, age distribution, birth and death of LTR-RTs (17.01% of the annotated genome). LTRharvest[24] and LTRdigest[25] were used for *de novo* prediction of long terminal repeat-retrotransposons (LTR-RTs). In this analysis, we separated a candidate LTR-RT by 1-15 kb from other candidates and flanked a pair of putative LTRs, which could range from 100 to 3,000 bp, and with a similarity >80%. Further, the identified LTR-RTs were classified according to the internal organization of the coding domains using REXdb v3.0[26] with LAST v983 (http://last.cbrc.jp) alignment tool[27]. If a LTR-RT candidate possessed a complete Gag-Pol protein sequence, it was retained as an intact LTR-RT (*I*). We saved LTR paralogous sequences from a blastn analysis and extracted 3 kb sequences both upstream and downstream of each detected LTR paralog to compare with the Gag-Pol protein sequences in the *Gypsy* database 2.0 [28] using tblastn. The LTR paralogs that lacked any Gag-Pol homologs in both the upstream and downstream sequences were considered to be solo-LTRs (*S*), and LTRs with Gag-Pol sequences on one side of flanking sequences were retained as truncated LTR-RTs (*T*). Superfamily classifications within the *Gypsy* and *Copia* classes are provided in **Supplementary Table 11**.

We estimated the timing of LTR-RT insertion based on the divergence between the 5' LTR and 3'-LTR of the same transposon. Each LTR pair was aligned using mafft (version 7.221)[29] with default settings. We employed the Kimura two-parameter method[30] to calculate the insertion time. The insertion time (T) was calculated following equation $T = K/2r$, where mutation rate $r = 1.5 \times 10^{-8}$ per site per year[31], and K represented the divergence of the LTRs from the intact LTR retrotransposons

(**Supplementary Fig. 9** and **10**). We also calculated the distances of intact LTR-RTs to an adjacent gene, and examined the relationships of the proximity to a gene and the insertion time of LTR-RTs.

To obtain further LTR-RT relationship insights, 5' LTR sequences of all LTR-RTs were compared against each other with blastn using Silix (version 1.2.9)[32]. Two LTRs were assigned to the same cluster if they mutually covered at least 70% of their lengths with an identity of at least 60% between them. Solo-LTRs (S) and truncated LTR-RTs (T) were also mapped to the same cluster containing 5' LTRs from the most similar intact LTR-RTs (*I*). Furthermore, ratios of solo-LTR-RTs and truncated LTR-RTs, respectively, to intact LTR-RTs (*S:I*, *T:I*) as well as their sums were assessed to study the removal rates of LTR-RTs over the past several million years. Then, we evaluated LTR-RT deletions using proportions of clusters with *S:I* values greater than 3. For an interspecific comparison, we also conducted LTR-RTs analysis including the other 14 asterids studied in the phylogeny analysis. We note here that we did not obtain any intact LTR-RTs for *Rhododendron williamsianum*[33].

LTR-RTs represented the highest proportion (17.01%) of the genome, while DNA TEs (6.36%), long interspersed nuclear element (2.20%), short interspersed nuclear element (0.73%), and rolling-circle transposon (0.27%) TEs together made up a minor fraction (9.56%) of the genome. *Gypsy* (11.90% of the genome sequence) and *Copia* (4.00%) LTR-RTs were unequally abundant. Repeat annotations are provided in **Supplementary Fig. 8** and **Supplementary Table 10**.

Nonsynonymous and synonymous (*K*a and *K*s) substitution rates were used to estimate the selective pressure of these LTR elements. Amino acid sequences of intact reverse transcriptase (RT) domains of full-length *Copia* and *Gypsy* superfamily were retrieved and assigned to the same cluster as LTRs. Conversion of amino acid alignments into the corresponding codon alignments was conducted with PAL2NAL (version 14)[34]. The YN model, which is implemented in KaKs_Calculator (version 2.0)[35], was utilized to perform selective pressure analyses.

Transcriptome assembly and gene expression analysis

A total of 19.6 G raw reads from RNA sequencing were obtained from leaf, flower, and stem tissues. These Illumina reads were processed using Trimmomatic (version 0.36)[36] and Cutadapt (version 1.13)[37] and aligned to the genome assembly with HiSat2 (version 2.1.0). Quality of raw and clean reads was assessed with FastQC (version 0.11.6) (https://www.bioinformatics.babraham.ac.uk/projects/fastqc/).

De novo transcriptome assembly was constructed by Trinity (version 2.0.6)[38], and reference genome-guided assemblies were generated with StringTie (version 1.3.5)[39] and Trinity (version 2.0.6). Subsequently, we combined all assemblies and then refined them using CD-HIT (version 4.6)[40]. Finally, 89,120 unique transcripts were predicted. In addition, we used these transcripts as expressed sequence tag (EST) evidence for gene prediction (see below).

For the flower coloration experiment, a total of 396.72 Gb high-quality clean data of 25 samples were mapped to the final assembly using HiSat2 after reads were filtered by Trimmomatic and Cutadapt. Only uniquely mapped paired-end reads were retained for counting and then for annotating gene models by featureCounts (version 1.5.3)[41]. Differential gene expression (DEG) analyses among the five stages of flower development were performed with DEseq2[42] and with FDR cut-off of 0.05 and log2 fold change (FC) cut-off of 1 (**Supplementary Fig. 20**).

Gene structural and functional annotation

Coding gene models were predicted by MAKER2 pipeline (version 2.31.9)[43]. We firstly masked repeat elements within the genome, then the repeat-masked genome was used

in both, evidence-based and *ab initio* gene prediction, strategies. For evidence-based gene prediction, we clustered and cleaned the protein sequences from the genome of *Arabidopsis thaliana*, *Actinidia chinensis*, and *Rhododendron delavayi*, and then generated protein homology evidence for gene prediction by CD-HIT (95% identity and 95% coverage). Subsequently, during MAKER2 v2.31.9 application, we used BLAST algorithms to align EST and protein data to the repeat-masked genome. The alignments were polished to predict gene models by Exonerate (version 2.4.0)[44]. For *ab initio* gene prediction with MAKER2, we used AUGUSTUS (version 3.3)[45,46] and then compared the outputs to evidence-based gene models to revise the gene predictions. To assess the quality of gene predictions, the annotation edit distance (AED) method was used to quantify the normalized distance between a gene model and its supporting evidence. Finally, we removed genes which had abnormity open reading frames (ORFs) or were too short (<= 50 aa) to produce a high-confidence annotated gene set.

We annotated non-coding RNAs (ncRNAs) using several databases and software packages. Firstly, tRNAs and their secondary structures were annotated using tRNAscan-SE (version 1.3.1)[47] with default parameters. We then annotated ribosomal RNAs (rRNAs) by RNAMMER (version 1.2)[48], and searched the Rfam database (version 9.1) (http://eggnogdb.embl.de/) using blastn to annotate other ncRNAs. A total of 32,999 protein-coding genes could be predicted, with average lengths of gene regions, transcript lengths, protein coding sequences, exons, and introns of 5,089.2 bp, 1416.3 bp, 1,288.7 bp, 259.7 bp, 403.1 bp, respectively (**Supplementary Table 5**). Other than coding genes, we also predicted 482 tRNAs, 64 rRNAs including eight 28S, six 18S and 50 5S rRNAs, and an additional 625 ncRNAs mainly containing 211 miRNAs, 16 tRNAs and 158 snoRNAs (**Supplementary Table 6**).

After the abovementioned structural annotations, we also annotated the functions of the predicted protein-coding genes based on sequence similarity searches by blat (version 36)[50], employing 30% identity and $1e^{-05}$ E-value cutoffs, against eight protein databases: (1) NR (https://www.ncbi.nlm.nih.gov/), (2) Swiss-Prot protein database[51], (3) Translated EMBL-Bank (as part of the International Nucleotide Sequence Database Collaboration TrEMBL[51] , (4) Pfam[52], (5) Cluster of Orthologous Groups for eukaryotic complete genomes (KOG) database, (6) KEGG (the Kyoto Encyclopedia of Genes and Genomes, Orthology) database[53], (7) GO[54], and (8) UniProt database[51]. For domain similarity predictions, the predicted protein sequences were annotated using InterProScan (version 5.27-66.0) (http://www.ebi.ac.uk/InterProScan) with default parameters.

Following above procedures, we concatenated the annotations derived from the eight databases searches to obtain the final gene functional annotations. By combining all strategies for gene function annotations, 96.44% of all predicted genes could be annotated with the following outcomes for at least one of the protein-related databases: NR (85.70%), Swiss-Prot (57.8%), TrEMBL (84.90%), Pfam (73.60%), and GO (75.9%) (**Supplementary Table 7**).

Supplementary Note 6. Gene families

In order to ascertain the evolutionary history of asterids, gene families or orthogroups of 17 species representing outgroups and the main clades of asterids were identified using OrthoFinder (version 2.3.1)[56]. Along with *Rhododendron simsii*, we selected five species of Ericales (, *Camellia sinensis*[57], *Actinidia chinensis*[58], *Rhododendron delavayi*[59], *R. williamsianum*, and *Vaccinium corymbosum*[60]), four asterid II (*Lactuca sativa*[61], *Helianthus annuus*[62], *Daucus carota*[63], and *Eucommia ulmoides*[64]), three asterid I (*Coffea canephora*[65], *Sesamum indicum*[66], *Solanum lycopersicum*[67]), one

Cornales (*Camptotheca acuminata*[68]), two rosid species (*Vitis vinifera* and *Arabidopsis thaliana*) (**Supplementary Table 12** and **13**). Among the identified 22,455 gene families, 6,269 families were shared among all these genomes. A total of 12 gene families (40 genes) were found to be specific to the assembled *R. simsii* genome when compared to the other 16 genomes.

Then, among the 17 analyzed genomes, the number of orthogroups were determined where a minimum of 76.5% of the species have single-copy genes in any given orthogroup; these 806 orthogroups were used in constructing a phylogenetic tree with *Vitis vinifera* and *Arabidopsis thaliana* as outgroups. DNA sequence matrices were created by MUSCLE (version 3.8.31)[69] using default settings, and concatenated amino acid sequences were trimmed using trimAI (version 1.2) (trimal -gt 0.8 -st 0.001 -cons 60)[70]. The trimmed alignments was used to construct a maximum likelihood (ML) tree using IQ-TREE (version 1.6.7)[71], with the optimal sequence evolution model (-m JTT+F+R5), Shimodaira-Hasegawa-like approximate likelihood-ratio test (SH-aLRT, -alrt 1000)[72], and ultrafast bootstrapping (-bb 1000)[73,74]. This ML tree and trimmed amino acid alignments of 10 single-copy orthogroups were then used as the inputs to estimate the divergence time using MCMCTREE implemented in PAML v4.9h[75] with the following parameters: 'burnin 100000, sampfreq 200, nsample 10000'. The phylogeny was calibrated using two fossils and a soft bound at three split nodes: (1) the stem node of *Rhododendron* (56 Mya)[76], (2) the crown node of ericales (89.8 Mya)[77], (3) asterids-rosids (116-126 Mya)[78].

Thereafter, 14,727 families were retained which were separated "Standard Deviation" of gene families with <100 from >=100 and which were shared among two species at least. Using those families and the previously generated time tree among the 17 species, we inferred gene family expansion and loss by CAFÉ (version 4.1)[79] with 0.05 *p*-value cutoff. Ultimately, 1,515 gene families were detected that have expanded, while 1,657 gene families were found to have contracted in the *R. simsii* lineage.

Supplementary Note 7. Transcription factors

We used PlantRegMap[80-82] to identify transcription factors (TFs) with homology to *Arabidopsis thaliana*. In total, we identified 1,684 TF genes for the *R. simsii* genome. Among all TFs, MYBs[83,84], basic helix-loop-helix (bHLH) proteins[85,86], WD40s[87], ERFs[88] and WRKYs[89] and their associated transcriptional complexes have been shown to regulate multiple enzymatic steps crucial in the production of flavonoids, especially anthocyanins, important secondary metabolites in a range of plant species. We further analyzed their phylogeny, gene conserved motifs and protein structures.

To identify the potential members of these five TF gene families in *R. simsii*, Hidden Markov Model (HMM) profiles of MYB (PF000249), bHLH (PF00010), WD40 (PF00400), ERF (PF00847) and WRKY (PF03106), respectively, were downloaded from Pfam and used as the query to search against protein sequences databases employing the HMMER software (http://hmmer.org/), with E-value thresholds set to 1e^{-10}. In addition, we used the outputs of PlantRegMap as supporting evidence of prediction. Furthermore, all obtained protein sequences were manually inspected with SMART (http://smart.embl-heidelberg.de/) to verify the presence of conserved domains, and protein sequences that did not contain conserved domains were not further considered. The NCBI Batch Web CD-Search Tool (https://www.ncbi.nlm.nih.gov/Structure/bwrpsb/bwrpsb.cgi) was used to confirm domains presence, employing default parameters. Members of these families were assigned to subgroups according to previous studies for *MYB* [83,84], *bHLH*[85,86], *WD40*[87], *ERF*[88], and *WRKY*[89]. A total of 155 *MYB* (further divided into 123 *R2R3*-, nine *3R*- and

23 *4R-MYB* genes), 119 *bHLH*, 156 *WD40*, 136 *ERF* genes and 74 WRKY protein domains in *R. simsii* were identified in our analysis. *MYB*, *bHLH*, *WD40*, *ERF*, and *WRKY* domains were classified into 32, 29, 35, 10 and eight groups, respectively (**Supplementary Fig. 23**).

In total of 738 *Arabidopsis* protein sequences for each of these five TF families (MYB: 132; bHLH: 169; WD40: 230; ERF: 122; WRKY: 85) were retrieved from The Arabidopsis Information Resource (TAIR) Arabidopsis Genome Annotation version 10[90]. Protein sequences (domain sequences in the case of the WRKY gene family) for *R. simsii* and *Arabidopsis* were aligned using mafft v7.221 and then trimmed by trimAl v1.2 with default parameters. IQ-TREE was used to reconstruct ML trees using the aligned sequences and a bootstrap test with 1,000 iterations. Then, the trees were rooted and plotted using FigTree (version 1.4.4)[91]. The conserved motifs and structure found for each TF family were predicted by TBtools (version 0.6644449)[92] (**Supplementary Fig. 24**).

Supplementary Note 8. Time-ordered gene regulatory network in flower color development

We selected 8,067 genes (618 TFs and 7,449 structural genes) for further analysis. These genes exhibited high levels of expression with an average TPM (Transcripts Per Kilobase Million) greater 0.5 and significant expression differences between any two pairs of samples among the five different flower developmental stages (**Fig. 4a**). Then, a *bHLH* transcription factor (*Rhsim13G0024200*) was selected as the initial node as it was highly expressed only at the first time-point but only weakly expressed at any of the following time-points, a prerequisite to generate time-ordered gene co-expression network (TO-GCN)[93]. Eight hierarchical gene regulatory modules (L1-L8, with nodes greater 20) centered on TFs were reconstructed using the suggested positive and negative cutoff values: 0.81 and -0.57, respectively, in C1+C2+ GCN by TO-GCN[93].

F3H[94] (flavanone 3-hydroxylase; EC:1.14.11.9) is the rate-limited enzyme for anthocyanin/flavonol biosynthesis. We aimed at identifying the upstream regulatory networks that modulate their respective expression. Firstly, we used the TO-GCN to predict candidates for direct regulators (TFs) of these two genes, which should be co-expressed with the specific structural gene at the same level or at earlier levels. We called these TFs appearing at the same level as the structural gene the first-order candidate regulators. Similarly, we inferred the second- and third-order candidate regulators at earlier levels, respectively.

Furthermore, we selected six core genes (*F3H* (*Rhsim11G0126300*, *Rhsim03G0111400*); *MYB* (*Rhsim08G0132300*); *C2H2* (*Rhsim10G0164300*); *C3H* (*Rhsim13G0068400*); *GRAS* (*Rhsim13G0080100*) (**Fig 5d** and **6b**) from identified upstream regulatory pathways and extracted for each of these genes their upstream 2kb enclosing their suspected gene expression regulatory sequences. Then, the putative TF binding sites for the suspected promoter sequences were predicted by querying PlantCARE[95] and PlantRegMap with *p*-value <= $1e^{-4}$ and *q*-value <= 0.05. Finally, we predicted 818 and 752 binding sites, respectively, via PlantRegMap and PlantCARE, for those identified key upstream pathway genes and illustrated them by using the R package drawProteins[96] (**Fig 5d** and **6b**).

Supplementary References

1. Chen, S., Zhou, Y., Chen, Y. & Gu, J. fastp: an ultra-fast all-in-one FASTQ preprocessor. *Bioinformatics* **34**, i884-i890 (2018).
2. Duan, Z. *et al.* A three-dimensional model of the yeast genome. *Nature* **465**,

363-367 (2010).
3. Koren, S. *et al.* Canu: scalable and accurate long-read assembly via adaptive *k*-mer weighting and repeat separation. *Genome Res.* **27**, 722-736 (2017).
4. Marcais, G. & Kingsford, C. A fast, lock-free approach for efficient parallel counting of occurrences of *K*-mers. *Bioinformatics* **27**, 764-770 (2011).
5. Vurture, G.W. *et al.* GenomeScope: fast reference-free genome profiling from short reads. *Bioinformatics* **33**, 2202-2204 (2017).
6. Liu, B. *et al.* Estimation of genomic characteristics by analyzing *K*-mer frequency in de novo genome projects. Preprint at https://arxiv.org/abs/1308.2012 (2013).
7. Li, H. Minimap2: pairwise alignment for nucleotide sequences. *Bioinformatics* **34**, 3094-3100 (2018).
8. Swindell, S.R. & Plasterer, T.N. SEQMAN. Contig assembly. *Methods Mol. Biol.* **70**, 75-89 (1997).
9. Tillich, M. *et al.* GeSeq - versatile and accurate annotation of organelle genomes. *Nucleic Acids Res.* **45**, W6-W11 (2017).
10. Ruan, J. & Li, H. Fast and accurate long-read assembly with wtdbg2. *Nat. Meth.* **17**, 155-158 (2020).
11. Chakraborty, M., Baldwin-Brown, J.G., Long, A.D. & Emerson, J.J. Contiguous and accurate de novo assembly of metazoan genomes with modest long read coverage. *Nucleic Acids Res.* **44**, e147 (2016).
12. Walker, B.J. *et al.* Pilon: an integrated tool for comprehensive microbial variant detection and genome assembly improvement. *PLoS One* **9**, e112963 (2014).
13. Dudchenko, O. *et al.* De novo assembly of the *Aedes aegypti* genome using Hi-C yields chromosome-length scaffolds. *Science* **356**, 92-95 (2017).
14. Durand, N.C. *et al.* Juicebox provides a visualization system for Hi-C contact maps with unlimited zoom. *Cell Systems* **3**, 99-101 (2016).
15. Xu, G.C. *et al.* LR_Gapcloser: a tiling path-based gap closer that uses long reads to complete genome assembly. *Gigascience* **8**, giy157, doi:10.1093/gigascience/giy157 (2019).
16. Pryszcz, L.P. & Gabaldón, T. Redundans: an assembly pipeline for highly heterozygous genomes. *Nucleic Acids Res.* **44**, e113 (2016).
17. Boratyn, G.M. *et al.* Domain enhanced lookup time accelerated BLAST. *Biol. Direct* **7**, 12 (2012).
18. Ou, S., Chen, J. & Jiang, N. Assessing genome assembly quality using the LTR Assembly Index (LAI). *Nucleic Acids Res.* **46**, e126 (2018).
19. Simao, F.A., Waterhouse, R.M., Ioannidis, P., Kriventseva, E.V. & Zdobnov, E.M. BUSCO: assessing genome assembly and annotation completeness with single-copy orthologs. *Bioinformatics* **31**, 3210-3212 (2015).
20. Li, H. Aligning sequence reads, clone sequences and assembly contigs with BWA-MEM. Preprint at https://arxiv.org/abs/1303.3997 (2013).
21. Kim, D., Langmead, B. & Salzberg, S.L. HISAT: a fast spliced aligner with low memory requirements. *Nat. Meth.* **12**, 357-360 (2015).
22. Li, H. & Durbin, R. Fast and accurate short read alignment with Burrows-Wheeler transform. *Bioinformatics* **25**, 1754-1760 (2009).
23. Durand, N.C. *et al.* Juicer provides a one-click system for analyzing loop-resolution Hi-C experiments. *Cell Systems* **3**, 95-98 (2016).
24. Ellinghaus, D., Kurtz, S. & Willhoeft, U. LTRharvest, an efficient and flexible software for de novo detection of LTR retrotransposons. *BMC Bioinf.* **9**, 18

(2008).
25. Steinbiss, S., Willhoeft, U., Gremme, G. & Kurtz, S. Fine-grained annotation and classification of de novo predicted LTR retrotransposons. *Nucleic Acids Res.* **37**, 7002-7013 (2009).
26. Neumann, P., Novak, P., Hostakova, N. & Macas, J. Systematic survey of plant LTR-retrotransposons elucidates phylogenetic relationships of their polyprotein domains and provides a reference for element classification. *Mob DNA* **10**, 1 (2019).
27. Kielbasa, S.M., Wan, R., Sato, K., Horton, P. & Frith, M.C. Adaptive seeds tame genomic sequence comparison. *Genome Res.* **21**, 487-493 (2011).
28. Llorens, C. *et al.* The Gypsy Database (GyDB) of mobile genetic elements: release 2.0. *Nucleic Acids Res.* **39**, D70-D74 (2011).
29. Katoh, K. & Standley, D.M. MAFFT multiple sequence alignment software version 7: improvements in performance and usability. *Mol. Biol. Evol.* **30**, 772-780 (2013).
30. Kimura, M. A simple method for estimating evolutionary rates of base substitutions through comparative studies of nucleotide sequences. *J. Mol. Evol.* **16**, 111-120 (1980).
31. Koch, M.A., Haubold, B. & Mitchell-Olds, T. Comparative evolutionary analysis of chalcone synthase and alcohol dehydrogenase loci in Arabidopsis, Arabis, and related genera (Brassicaceae). *Mol. Biol. Evol.* **17**, 1483-1498 (2000).
32. Miele, V., Penel, S. & Duret, L. Ultra-fast sequence clustering from similarity networks with SiLiX. *BMC Bioinf.* **12**, 116 (2011).
33. Soza, V.L. *et al.* The *Rhododendron* genome and chromosomal organization provide insight into shared whole genome duplications across the heath family (Ericaceae). *Genome Biol. Evol.* **11**, 3357-3371 (2019).
34. Suyama, M., Torrents, D. & Bork, P. PAL2NAL: robust conversion of protein sequence alignments into the corresponding codon alignments. *Nucleic Acids Res.* **34**, W609-W612 (2006).
35. Wang, D., Zhang, Y., Zhang, Z., Zhu, J. & Yu, J. KaKs_Calculator 2.0: a toolkit incorporating gamma-series methods and sliding window strategies. *Genomics, Proteomics & Bioinformatics* **8**, 77-80 (2010).
36. Bolger, A.M., Lohse, M. & Usadel, B. Trimmomatic: a flexible trimmer for Illumina sequence data. *Bioinformatics* **30**, 2114-2120 (2014).
37. Martin, M. Cutadapt removes adapter sequences from high-throughput sequencing reads. *EMBnet Journal* **17**, 10-12 (2011).
38. Grabherr, M.G. *et al.* Full-length transcriptome assembly from RNA-Seq data without a reference genome. *Nat. Biotechnol.* **29**, 644-652 (2011).
39. Pertea, M. *et al.* StringTie enables improved reconstruction of a transcriptome from RNA-seq reads. *Nat. Biotechnol.* **33**, 290-295 (2015).
40. Fu, L., Niu, B., Zhu, Z., Wu, S. & Li, W. CD-HIT: accelerated for clustering the next-generation sequencing data. *Bioinformatics* **28**, 3150-3152 (2012).
41. Liao, Y., Smyth, G.K. & Shi, W. featureCounts: an efficient general purpose program for assigning sequence reads to genomic features. *Bioinformatics* **30**, 923-930 (2014).
42. Love, M.I., Huber, W. & Anders, S. Moderated estimation of fold change and dispersion for RNA-seq data with DESeq2. *Genome Biol.* **15**, 550 (2014).
43. Holt, C. & Yandell, M. MAKER2: an annotation pipeline and genome-database management tool for second-generation genome projects. *BMC*

Bioinf. **12**, 491 (2011).
44. Slater, G.S. & Birney, E. Automated generation of heuristics for biological sequence comparison. *BMC Bioinf.* **6**, 31 (2005).
45. Keller, O., Kollmar, M., Stanke, M. & Waack, S. A novel hybrid gene prediction method employing protein multiple sequence alignments. *Bioinformatics* **27**, 757-763 (2011).
46. Stanke, M., Diekhans, M., Baertsch, R. & Haussler, D. Using native and syntenically mapped cDNA alignments to improve de novo gene finding. *Bioinformatics* **24**, 637-644 (2008).
47. Lowe, T.M. & Eddy, S.R. tRNAscan-SE: a program for improved detection of transfer RNA genes in genomic sequence. *Nucleic Acids Res.* **25**, 955-964 (1997).
48. Lagesen, K. *et al.* RNAmmer: consistent and rapid annotation of ribosomal RNA genes. *Nucleic Acids Res.* **35**, 3100-3108 (2007).
49. Gardner, P.P. *et al.* Rfam: updates to the RNA families database. *Nucleic Acids Res.* **37**, D136-D140 (2009).
50. Kent, W.J. BLAT--the BLAST-like alignment tool. *Genome Res.* **12**, 656-664 (2002).
51. Bairoch, A. & Apweiler, R. The SWISS-PROT protein sequence database and its supplement TrEMBL in 2000. *Nucleic Acids Res.* **28**, 45-48 (2000).
52. Finn, R.D. *et al.* Pfam: the protein families database. *Nucleic Acids Res.* **42**, D222-D230 (2014).
53. Kanehisa, M. & Goto, S. KEGG: Kyoto Encyclopedia of Genes and Genomes. *Nucleic Acids Res.* **28**, 27-30 (2000).
54. Harris, M.A. *et al.* The Gene Ontology (GO) database and informatics resource. *Nucleic Acids Res.* **32**, D258-D261 (2004).
55. Quevillon, E. *et al.* InterProScan: protein domains identifier. *Nucleic Acids Res.* **33**, W116-W120 (2005).
56. Emms, D.M. & Kelly, S. OrthoFinder: phylogenetic orthology inference for comparative genomics. *Genome Biol.* **20**, 238 (2019).
57. Wei, C. *et al.* Draft genome sequence of *Camellia sinensis* var. *sinensis* provides insights into the evolution of the tea genome and tea quality. *Proc. Natl. Acad. Sci. U. S. A.* **115**, E4151-E4158 (2018).
58. Wang, J.P. *et al.* Two likely auto-tetraploidization events shaped kiwifruit genome and contributed to establishment of the Actinidiaceae family. *iScience* **7**, 230-240 (2018).
59. Zhang, L. *et al.* The draft genome assembly of *Rhododendron delavayi* Franch. var. *delavayi*. *Gigascience* **6**, 1-11 (2017).
60. Colle, M. *et al.* Haplotype-phased genome and evolution of phytonutrient pathways of tetraploid blueberry. *Gigascience* **8**, giz012 (2019).
61. Reyes-Chin-Wo, S. *et al.* Genome assembly with in vitro proximity ligation data and whole-genome triplication in lettuce. *Nat. Commun.* **8**, 14953 (2017).
62. Badouin, H. *et al.* The sunflower genome provides insights into oil metabolism, flowering and Asterid evolution. *Nature* **546**, 148-152 (2017).
63. Iorizzo, M. *et al.* A high-quality carrot genome assembly provides new insights into carotenoid accumulation and asterid genome evolution. *Nat. Genet.* **48**, 657-666 (2016).
64. Wuyun, T.N. *et al.* The hardy rubber tree genome provides insights into the evolution of polyisoprene biosynthesis. *Mol. Plant* **11**, 429-442 (2018).
65. Denoeud, F. *et al.* The coffee genome provides insight into the convergent

evolution of caffeine biosynthesis. *Science* **345**, 1181-1184 (2014).
66. Wang, L. *et al.* Genome sequencing of the high oil crop sesame provides insight into oil biosynthesis. *Genome Biol.* **15**, R39 (2014).
67. Sato, S. *et al.* The tomato genome sequence provides insights into fleshy fruit evolution. *Nature* **485**, 635-641 (2012).
68. Zhao, D. *et al.* De novo genome assembly of *Camptotheca acuminata*, a natural source of the anti-cancer compound camptothecin. *Gigascience* **6**, 1-7 (2017).
69. Edgar, R.C. MUSCLE: multiple sequence alignment with high accuracy and high throughput. *Nucleic Acids Res.* **32**, 1792-1797 (2004).
70. Capella-Gutiérrez, S., Silla-Martínez, J.M. & Gabaldón, T. trimAl: a tool for automated alignment trimming in large-scale phylogenetic analyses. *Bioinformatics* **25**, 1972-1973 (2009).
71. Nguyen, L.T., Schmidt, H.A., von Haeseler, A. & Minh, B.Q. IQ-TREE: a fast and effective stochastic algorithm for estimating maximum-likelihood phylogenies. *Mol. Biol. Evol.* **32**, 268-274 (2015).
72. Guindon, S. *et al.* New algorithms and methods to estimate maximum-likelihood phylogenies: assessing the performance of PhyML 3.0. *Syst. Biol.* **59**, 307-321 (2010).
73. Hoang, D.T., Chernomor, O., von Haeseler, A., Minh, B.Q. & Vinh, L.S. UFBoot2: improving the ultrafast bootstrap approximation. *Mol. Biol. Evol.* **35**, 518-522 (2018).
74. Minh, B.Q., Nguyen, M.A. & von Haeseler, A. Ultrafast approximation for phylogenetic bootstrap. *Mol. Biol. Evol.* **30**, 1188-1195 (2013).
75. Yang, Z. PAML 4: phylogenetic analysis by maximum likelihood. *Mol. Biol. Evol.* **24**, 1586-1591 (2007).
76. Collinson, M.E. & Crane, P.R. *Rhododendron* seeds from the Palaeocene of southern England. *Bot. J. Linn. Soc.* **76**, 195-205 (1978).
77. Nixon, K.C. & Crepet, W.L. Late Cretaceous fossil flowers of Ericalean affinity. *Am. J. Bot.* **80**, 616-623 (1993).
78. Li, H.T. *et al.* Origin of angiosperms and the puzzle of the Jurassic gap. *Nature Plants* **5**, 461-470 (2019).
79. De Bie, T., Cristianini, N., Demuth, J.P. & Hahn, M.W. CAFE: a computational tool for the study of gene family evolution. *Bioinformatics* **22**, 1269-1271 (2006).
80. Tian, F., Yang, D.C., Meng, Y.Q., Jin, J. & Gao, G. PlantRegMap: charting functional regulatory maps in plants. *Nucleic Acids Res.* **48**, D1104-D1113 (2019).
81. Jin, J. *et al.* An *Arabidopsis* transcriptional regulatory map reveals distinct functional and evolutionary features of novel transcription factors. *Mol. Biol. Evol.* **32**, 1767-1773 (2015).
82. Jin, J. *et al.* PlantTFDB 4.0: toward a central hub for transcription factors and regulatory interactions in plants. *Nucleic Acids Res.* **45**, D1040-D1045 (2017).
83. Dubos, C. *et al.* MYB transcription factors in *Arabidopsis*. *Trends Plant Sci.* **15**, 573-581 (2010).
84. Li, X. *et al.* Genome-wide identification, evolution and functional divergence of MYB transcription factors in Chinese white pear (*Pyrus bretschneideri*). *Plant Cell Physiology* **57**, 824-847 (2016).
85. Pires, N. & Dolan, L. Origin and diversification of basic-helix-loop-helix proteins in plants. *Mol. Biol. Evol.* **27**, 862-874 (2010).

86. Wei, K. & Chen, H. Comparative functional genomics analysis of bHLH gene family in rice, maize and wheat. *BMC Plant Biol.* **18**, 309 (2018).
87. Li, Q. *et al.* Genome-wide analysis of the WD-repeat protein family in cucumber and *Arabidopsis*. *Mol. Genet. Genomics* **289**, 103-124 (2014).
88. Nakano, T., Suzuki, K., Fujimura, T. & Shinshi, H. Genome-wide analysis of the ERF gene family in *Arabidopsis* and rice. *Plant Physiol.* **140**, 411-432 (2006).
89. Huang, S. *et al.* Genome-wide analysis of WRKY transcription factors in *Solanum lycopersicum*. *Mol. Genet. Genomics* **287**, 495-513 (2012).
90. Lamesch, P. *et al.* The Arabidopsis Information Resource (TAIR): improved gene annotation and new tools. *Nucleic Acids Res.* **40**, D1202-D1210 (2012).
91. Morariu, V.I., Srinivasan, B.V., Raykar, V.C., Duraiswami, R. & Davis, L.S. Automatic online tuning for fast Gaussian summation. in *Advances in Neural Information Processing Systems* 1113-1120 (2009).
92. Chen, C. *et al.* TBtools - an integrative toolkit developed for interactive analyses of big biological data. *Mol Plant* **13**, 1194-1202 (2020).
93. Chang, Y.M. *et al.* Comparative transcriptomics method to infer gene coexpression networks and its applications to maize and rice leaf transcriptomes. *Proc. Natl. Acad. Sci. U. S. A.* **116**, 3091-3099 (2019).
94. Forkmann, G., Heller, W. & Grisebach, H. Anthocyanin biosynthesis in flowers of *Matthiola incana* flavanone 3-and flavonoid 3′-hydroxylases. *Zeitschrift für Naturforschung C* **35**, 691-695 (1980).
95. Lescot, M. *et al.* PlantCARE, a database of plant cis-acting regulatory elements and a portal to tools for in silico analysis of promoter sequences. *Nucleic Acids Res.* **30**, 325-327 (2002).
96. Brennan, P. drawProteins: a Bioconductor/R package for reproducible and programmatic generation of protein schematics. *F1000 Research* **7**, 1105 (2018).
