# Supplementary material for: Chromosome-level genome assembly of a parent species of widely cultivated azaleas
Source: Nat Commun. 2020 Oct 19;11:5269. doi: 10.1038/s41467-020-18771-4 (PMC7572368; doi:10.1038/s41467-020-18771-4)
Supplement: Supplementary file 3 — Reporting Summary [file 41467_2020_18771_MOESM3_ESM.pdf]

nature research

Corresponding author(s): Jian-Feng Mao, Yves Van de Peer

Last updated by author(s): Aug 24, 2020

Reporting Summary

Nature Research wishes to improve the reproducibility of the work that we publish. This form provides structure for consistency and transparency in reporting. For further information on Nature Research policies, see our Editorial Policies and the Editorial Policy Checklist.

Statistics

For all statistical analyses, confirm that the following items are present in the figure legend, table legend, main text, or Methods section.

n/a	Confirmed	
☐	☒	The exact sample size (*n*) for each experimental group/condition, given as a discrete number and unit of measurement
☐	☒	A statement on whether measurements were taken from distinct samples or whether the same sample was measured repeatedly
☐	☒	The statistical test(s) used AND whether they are one- or two-sided<br>*Only common tests should be described solely by name; describe more complex techniques in the Methods section.*
☒	☐	A description of all covariates tested
☐	☒	A description of any assumptions or corrections, such as tests of normality and adjustment for multiple comparisons
☐	☒	A full description of the statistical parameters including central tendency (e.g. means) or other basic estimates (e.g. regression coefficient) AND variation (e.g. standard deviation) or associated estimates of uncertainty (e.g. confidence intervals)
☐	☒	For null hypothesis testing, the test statistic (e.g. F, t, r) with confidence intervals, effect sizes, degrees of freedom and P value noted<br>*Give P values as exact values whenever suitable.*
☒	☐	For Bayesian analysis, information on the choice of priors and Markov chain Monte Carlo settings
☒	☐	For hierarchical and complex designs, identification of the appropriate level for tests and full reporting of outcomes
☐	☒	Estimates of effect sizes (e.g. Cohen's d, Pearson's r), indicating how they were calculated

Our web collection on statistics for biologists contains articles on many of the points above.

Software and code

Policy information about availability of computer code

Data collection	No software was used for data collection.
Data analysis	SAMtools (version 1.2); OrthoFinder (version 2.3.1); CAFÉ (version 4.0); TO-GCN; fastp (version 0.19.3); SMARTdenovo (version 1.0); WTDBG (version 2.1); Canu (version 1.7); FALCON-Phase (version 0.1.0-beta); quickmerge (version 0.2); pilon (version 1.22); Juicebox (version 1.8); 3D-DNA (version 180922); LR_Gapcloser (version 1.1); BWA-MEM (version 0.7.17); minimap2 (version 2.11-r797); HiSat2 (version 2.1.0); Juicer (version 1.5); RepeatModeler (version 1.0.10); RepeatMasker (version 4.0.7, rmblast-2.2.28); codeml; tRNAscan-SE (version 1.3.1); RNAmmer (version 1.2); BLASTn (version 2.2.28+); blat (version 36); InterProScan (version 5.27-66.0); Centurion (version 0.1); MUSCLE (version 3.8.31); trimAl (version 1.2); IQ-TREE (version 1.6.7); PAML package (version 4.9h); DupGen_finder; KaKs_Calculator (version 2.0); PAL2NAL (version 14); Semi-Automated Validation Infrastructure (version 3.02); Ensemble Enzyme Prediction Pipeline (E2P2) package (version 3.1); PlantRegMap; Trimmomatic (version 0.36); Cutadapt (version 1.13); featureCounts (version 1.6.3); DEseq2; Cytoscape (version 3.5.1); PlantCARE; drawProteins; clusterProfiler (version 3.6.0).

For manuscripts utilizing custom algorithms or software that are central to the research but not yet described in published literature, software must be made available to editors and reviewers. We strongly encourage code deposition in a community repository (e.g. GitHub). See the Nature Research guidelines for submitting code & software for further information.

April 2020

Data

Policy information about availability of data

All manuscripts must include a data availability statement. This statement should provide the following information, where applicable:

- Accession codes, unique identifiers, or web links for publicly available datasets
- A list of figures that have associated raw data
- A description of any restrictions on data availability

The raw sequence data of R. simsii genome sequencing and RNA sequencing have been deposited in NCBI under the accession number SRP229032 (Bio-Project: PRJNA588298). Genome assembly, repeat and gene annotation, gene expression profiles could be downloaded and explored online under URL: http://rhododendron.plantgenie.org/. The source data underlying Figs. 1, 2a, b, d, 3, 4b, 5a, b, d, 6a, b, d, and Table 1, as well as Supplementary Figs. 5, 13-14, 16-18, and 21-24 are provided as a Source Data file. Global Biodiversity Information Facility database [https://www.gbif.org/]; database of retrotransposon protein domains (REXdb) [http://repeatexplorer.org/]; Gypsy database [http://gydb.org]; Actinidia chinensis [ftp://ftp.ncbi.nlm.nih.gov/genomes/all/GCA/003/024/255/GCA_003024255.1_Red5_PS1_1.69.0/]; Arabidopsis thaliana [https://phytozome-next.jgi.doe.gov/info/Athaliana_TAIR10]; Camellia sinensis [http://tpia.teaplant.org/download.html]; Camptotheca acuminata [https://datadryad.org/stash/dataset/doi:10.5061/dryad.nc8qr]; Coffea canephora [http://coffee-genome.org/]; Daucus carota [https://phytozome-next.jgi.doe.gov/info/Dcarota_v2_0]; Eucommia ulmoides [ftp://download.big.ac.cn/gwh/Plants/Eucommia_ulmoides_hardy_rubberv0_GWHAAAL00000000/]; Helianthus annuus [ftp://ftp.ncbi.nlm.nih.gov/genomes/all/GCF/002/127/325/GCF_002127325.1_HanXRQr1.0]; Lactuca sativa [ftp://ftp.ncbi.nlm.nih.gov/genomes/all/GCF/002/870/075/GCF_002870075.1_Lsat_Salinas_v7]; Primula vulgaris [https://opendata.earlham.ac.uk/opendata/data/primula/sci_reports_Cocker_et_al_2018/]; Rhododendron delavayi [http://gigadb.org/dataset/100331]; Rhododendron williamsianum [https://genomevolution.org/coge/GenomeInfo.pl?gid=51210]; Sesamum indicum [http://ocri-genomics.org/Sinbase/]; Solanum lycopersicum [https://phytozome-next.jgi.doe.gov/info/Slycopersicum_ITAG2_4]; Vaccinium corymbosum [http://gigadb.org/dataset/100537]; Vitis vinifera [https://www.ncbi.nlm.nih.gov/assembly/GCF_000003745.3].

Field-specific reporting

Please select the one below that is the best fit for your research. If you are not sure, read the appropriate sections before making your selection.

- [x] Life sciences
- [ ] Behavioural & social sciences
- [ ] Ecological, evolutionary & environmental sciences

For a reference copy of the document with all sections, see nature.com/documents/nr-reporting-summary-flat.pdf

Life sciences study design

All studies must disclose on these points even when the disclosure is negative.

Sample size	No sampling size estimation was required in our work. One individual tree was sampled for de novo genome assembly, and flower tissues of five individuals from the same population selected for genome sequencing were sampled for the RNA-seq experiments. We have performed 25 RAN-seq for flower tissues of five developmental stages, which was enough for constructing time-ordered gene co-expression networks.
Data exclusions	As a commonly applied step, data filtering was executed to read data and gene expression data, detailed data filtering processes were provided in the method section. No sample was excluded in such filtering process.
Replication	For the RNA-seq experiments generated in our study, five biological replicates were generated to ensure and valid biological interpretation of the gene expression analysis. All attempts at replication were successful, and results could be reproduced.
Randomization	Individuals for RNA-seq were sampled randomly from the same population but otherwise randomization was not applicable in this study.
Blinding	Blinding test was not performed as it was not relevant to this study.

Reporting for specific materials, systems and methods

We require information from authors about some types of materials, experimental systems and methods used in many studies. Here, indicate whether each material, system or method listed is relevant to your study. If you are not sure if a list item applies to your research, read the appropriate section before selecting a response.

Materials & experimental systems

n/a	Involved in the study
x	☐ Antibodies
x	☐ Eukaryotic cell lines
x	☐ Palaeontology and archaeology
x	☐ Animals and other organisms
x	☐ Human research participants
x	☐ Clinical data
x	☐ Dual use research of concern

Methods

n/a	Involved in the study
x	☐ ChIP-seq
x	☐ Flow cytometry
x	☐ MRI-based neuroimaging
