# Supplementary material for: Chromosome-level genome assembly of a parent species of widely cultivated azaleas
Source: Nat Commun. 2020 Oct 19;11:5269. doi: 10.1038/s41467-020-18771-4 (PMC7572368; doi:10.1038/s41467-020-18771-4)
Supplement: Supplementary file 2 — Peer Review [file 41467_2020_18771_MOESM2_ESM.pdf]

Reviewers' comments:

Reviewer #1 (Remarks to the Author):

Review: Chromosome-level genome assembly of Rhododendron simsii, a parent species of widely cultivated azaleas

The authors described the genome assembly of Rhododendron simsii and compared it with 16 other species, including the two other Rhododendron genomes available. They placed four ancient WGD for the Ericales order. They also reconstructed metabolic pathways and regulatory networks for flower-related genes, and show how different types of duplications can contribute to key innovation for Rhododendron.

While the manuscript is well written, we have several major issues with the paper.

Major comments:
The authors should be more careful about stating whether a transcription factor is a regulator of a pathway. Just because a transcription factor is co-expressed with an enzyme does not mean that it is regulating the enzyme (e.g., line 331)! Similarly, line 340 makes a strong claim about a regulatory relationship, but the provided reference (74) is referencing a completely irrelevant study 'PAML 4: phylogenetic analysis by maximum likelihood'. This is an unacceptable overinterpretation of the co-expression networks.

Figures 5-6: It is surprising that the enzymes (peripheral nodes) from the pathways in panels A-C are not connected, i.e., co-expressed. This indicates that perhaps the co-expression networks are not biologically relevant.

Minor comments:
Line 107 and 120: The authors give two different values for heterozygosity (~1.78% and ~1.07%). Please clarify in the main text which is the difference.

Line 121: the authors mentioned that SAMtools was used to detect SNPs, but in the main material and methods it is not described.

Regarding the methods used to reconstruct the species tree, the authors used 806 low-copy orthologs (line 531). Could the authors explain which were the filters used to define the low copy orthologs?, i.e. how many copies were allowed per species, or how many species with copies, or which copy was used (randomly selected?).

For the estimation of the divergence dates, the authors used Timetree, which is not the most common tool used for this kind of analysis. There are many softwares available that provide a wider range of parameters and choices. I am concerned about this part since the estimation of the dates of the events is an important part of the manuscript. It would be nice if the authors can explain why they use Timetree and if the results are comparable with other methods.

Figure 1B: The Ks distribution in the upper right from the orthologs between Primula vulgaris and Vitis vinifera shows a different pattern than the species tree and the other Ks distribution. This should be clarified or discussed.

For the discussion section I would suggest the authors to discuss their findings about the WGDs and the findings in Rhododendron williamsianum [Soza et al., 2019]

Line 46: Please break the large sentence into smaller sentences.

Figures 1+3: These figures are so packed that the text is unreadable. Please consider breaking the figure up into two figures, or moving some of the panels into the supplement.

Figure 3: What do the heatmaps to the left of the genes indicate? I assume it is gene expression, but valuable information does gene expression bring to the paper? What do the columns in the heatmap correspond to? Please discuss.

Line 273: The authors reference Figure 3A+B when discussing the duplication percentages for the metabolic pathway. However, these figures do not contain duplication percentages.

Figure 4B: Judging from the heatmap, it seems to me that L5 is a more mature stage than L6,7,8. Please consider revising the figure.

Figure 5: Since these genes are co-expressed, consider whether it is needed to show an expression heatmap for

Reviewer #2 (Remarks to the Author):

Rhododendron is an important ornamental plant, which has different cultivars with distinct colors and flowering periods. A high-quality genome sequence will help to decipher novel regulatory mechanisms. In this manuscript, the authors reported their chromosome-scale assembly of R. simsii genome, and their preliminary analysis. The results are interesting but also need further in-depth confirmation and functional analysis. My major concerns are:
1. The upstream part of the carotenoid biosynthetic pathway is wrong. Carotenoids are synthesized from the MEP pathway. Although the authors labeled "MEP" pathway in their figures, but the pathway in figures and tables is the "MVA" pathway, which generally does not provide the substrate for carotenoid biosynthesis. Therefore, the analysis of carotenoid biosynthesis and regulation is entirely wrong.
2. I doubt the annotation of UGTCs2 for crocin biosynthesis in this manuscript. I don't think Rhododendron synthesizes crocin. It is a question what these UGTCs2 members indeed do. They might take part in other metabolic pathways, but most probably not for crocin.
3. The tandem arrangement of duplicated genes is not restricted to UGTGCs2, 4CL, and a few other enzymes as the authors identified in the manuscript. I looked up the annotation results in the supplemental files, and found such tandem duplication also exists for a large number of transcription factors. For example, there are 13 members of GRAS from 13G0079200 to 13G0080600, 10 of NAC from 11G0114800 to 11G0117000. There are also clusters of ERFs and MYBs. I wish the authors could provide their cloning and Sanger sequencing results to prove the existence of these clusters, instead of some mistakes in their assembly.
Taken together, I think such a study is interesting and important itself, but the annotation of the metabolic pathways is not acceptable, and the presence of a large number of large gene clusters also definitely needs to be further confirmed before this manuscript can be considered for an acceptance.

Reviewer #3 (Remarks to the Author):

Yang Et al report a chromosome scale genome for the important floriculture crop Rhododendron. They utilize this high-quality genome along with comparative genomics and time-ordered gene expression to identify biosynthesis pathways and regulatory networks of key flower color pigmentation genes. Rhododendron has a high proportion of tandem or proximally duplicated genes in pigmentation

pathways and the authors hypothesize these are involved in the evolution of flower color diversity in this species. I read this paper with interest and found the genome assembly, annotation, comparative genomics, and expression analyses well-documented and robust, especially compared to other recent genome papers. I have no concerns related to the technical aspects of this manuscript. I have a few minor comments that I feel would strengthen or clarify the manuscript.

Lines 272-290: The findings on tandem duplication of flower coloration genes are interesting, but I have a few comments. Is the proportion of tandemly or proximally duplicated genes significantly different for flower related genes compared to the genome wide average? Typically 15-30% of genes in plant genomes are tandemly duplicated, so a formal statistical test will test if enriched duplication contributes to this trait.
It is also unclear if this proportion of TD flower coloration genes is different from other plant genomes.

Time-ordered expression dynamics of flower pigmentation are discussed in the next section, but it would be interesting to include expression analysis of TD/PDs at the single gene level in this section. TDs have high birth/death rates, with many having no expression at all, so it would be interesting to see if these duplicated genes are functioning during flower development.

Line 386: The number of flowering related genes is higher in Rhododendron that Arabidopsis, but how does this compare with other species? I'm not sure if orthologs from other species are well-characterized, but this may be testable using the Orthogroup results.

Line 458: How do the individuals used for the expression analysis compare to the individual used for genome assembly? Are they the same cultivar?

Line 488: FALCON-Phase was used for assembly, but the assembly of multiple haplotypes was not reported.

Two draft assemblies were merged together, but these are not discussed. It would be useful to list the two programs that produced the 'best' assembly and the metrics of the unmerged and merged versions.

Line 531 orthogroupsof to "orthogroups of"

Reviewer #4 (Remarks to the Author):

Review of Yang et al.

This paper reports the sequencing, assembly and annotation of the genome of Rhododendron simsii, a parent species of widely cultivated azaleas. The assembly used PacBio long reads and Illumina short reads and also H-C data. It achieved a chromosome-level assembly with 13 chromosomes covering 91% of the genome and with a scaffold N50 of 36 Mb. In addition, the authors reconstructed the entire metabolic pathways for anthocyanins and carotenoids and their regulatory networks, suggesting that transcription factors MYB, bHLH, and WD40 may collectively regulate anthocyanin accumulation in R. simsii, particularly at the initial stages of flower coloration, and WRKY transcription factors may control progressive flower coloring at later stages. While the paper is in general well written, I have a number of comments and questions.

First, the authors mentioned "The previous lack of a high-quality Rhododendron species whole-genome sequence", but did not give much detail of the quality of previous assemblies, so it is difficult to judge the reliability of the results of comparative analyses with other species. When a genome is not well assembled and annotated, it is difficult to know how many genes and how many copies of a

gene are there in the genome.

Second, I did not see how redundancy was removed from the assembly and how the authors distinguished between gene duplication and redundancy. This is important. For example, how do you know the 425 genes related to flowering-time control were all or almost all real, so that the number is really considerably larger than that (280) in Arabidopsis?

Third, the authors identified 5 WGDs among the 5 lineages studied in Ericales (Fig. 1). That implies very frequent WGD events. I wonder how reliable the inferences were. In particular, a WGD occurred soon after the divergence between the Camellia genus and the common ancestor of the Actinidia and Rhododendron genera and a WDG occurred in the lineage. Why they were not due to a single WDG event?

Fourth, the statement "we detected a 2:4 syntenic relationship between R. simsii and Actinidia chinensis29 (Fig. 1d), a 2:1 syntenic relationship between R. simsii and Vitis vinifera20, and a 1:4 syntenic relationship between V. vinifera and V. corymbosum, which provided additional proof for a shared WGD event among R. simsii, V. corymbosum, and A. chinensis" is not easy to understand. How about change it to, "we detected a 2:1 syntenic relationship between R. simsii and Vitis vinifera20, a 4:2 syntenic relationship between Actinidia chinensis and R. simsii29, and a 4:1 syntenic relationship between V. corymbosum and V. vinifera, which provided additional proof for a shared WGD event among R. simsii, V. corymbosum, and A. chinensis" ?

Fifth, in Fig. 1 and throughout the paper, there are many cases where Ks is larger than 2. One should be aware that a Ks > 2 is difficult to estimate reliably. And so are the ratios Ka/Ks.

Sixth, Lines 218-219: Please define "proximal", "transposed" and "dispersed" duplicates.

Seventh, Line 308 and through out the paper, you used "regulatory interactions" or "regulatory network". To be qualified for these terms, you must have identified the TF binding sites on the promoters of the putative target genes. It seems to me you have not done that and if this is case, you should write "co-expression network" rather than "regulatory network".

Eighth, Lines 489-491: Which assemblers gave the two optimal assemblies v0.3 and v0.4?

Ninth, Line 496: How did you decide "five rounds of Pillon polishing"?

Tenth, in MAKER2 gene predictions, which method(s) was used for ab initio predictions?

Reviewer #1 (Remarks to the Author):

1.1. The authors should be more careful about stating whether a transcription factor is a regulator of a pathway. Just because a transcription factor is co-expressed with an enzyme does not mean that it is regulating the enzyme (e.g., line 331)! Similarly, line 340 makes a strong claim about a regulatory relationship, but the provided reference (74) is referencing a completely irrelevant study 'PAML 4: phylogenetic analysis by maximum likelihood'. This is an unacceptable overinterpretation of the co-expression networks.

Response: We agree with the reviewer that co-expression is not proof of a regulatory relationship between a TF and a specific gene/pathway. Here, we replaced "regulatory" with "co-expression" throughout our revised manuscript, so as to avoid potential overinterpretation. We would like to point out that "74" (line 340, line 391 in the revised manuscript) is not referring to a reference, but is indicating the number of TFs found in the subnetwork related to anthocyanin/flavonol biosynthesis. We reformatted the sentence in order to avoid potential misunderstanding. Please see lines 376-384 in the main text of revised version (with track changes).

1.2. Figures 5-6: It is surprising that the enzymes (peripheral nodes) from the pathways in panels A-C are not connected, i.e., co-expressed. This indicates that perhaps the co-expression networks are not biologically relevant.

Response: With the construction of the gene co-expression networks, we wanted to focus on the co-expressed TFs and the structural genes (enzymatic genes), but not on the co-expression among structural genes. So, we only showed the edges (indicative of co-expression) among TFs and structural genes in the subnetworks in Figures 5&6. If one looks into the gene expression of each structural gene (indicated by the heatmaps under the gene IDs in Figures 5a+b,6a and Supplementary Fig. 22), it is clear that the structural genes are indeed co-expressed. We have now added the following sentence 'Edges between structural genes are not shown' to the legends of Fig. 5&6 and the corresponding Supplementary Figures in the revised version of our manuscript.

Minor comments:

1.3. Line 107 and 120: The authors give two different values for heterozygosity (~1.78% and ~1.07%). Please clarify in the main text which is the difference.

Response: Done. Please see lines 110-115 in the main text of the revised manuscript (with track changes).

1.4. Line 121: the authors mentioned that SAMtools was used to detect SNPs, but in the main material and methods it is not described.

Response: The SAMtools was run with default settings. We have now indicated this in the main text of the revised manuscript (see track changes, lines 111-114).

1.5. Regarding the methods used to reconstruct the species tree, the authors used 806 low-copy orthologs (line 531). Could the authors explain which were the filters used

to define the low copy orthologs? i.e. how many copies were allowed per species, or how many species with copies, or which copy was used (randomly selected?).

Response: In the revised version of our manuscript, we now provide more information on the identification of low-copy orthologs, alignment construction, and ML tree inference, and also phylogenetic dating. Please see lines 579-587 in the main text (track changes).

1.6. For the estimation of the divergence dates, the authors used Timetree, which is not the most common tool used for this kind of analysis. There are many softwares available that provide a wider range of parameters and choices. I am concerned about this part since the estimation of the dates of the events is an important part of the manuscript. It would be nice if the authors can explain why they use Timetree and if the results are comparable with other methods.

Response: For the revised version of our manuscript, we have used the MCMCTree programme in the PAML package (version 4.9h), together with two fossil data and a soft bound. This is now described in more detail in the M&M: "The ML tree was then used as an input tree to estimate the divergence time using the MCMCTree programme in the PAML package (version 4.9h) (Supplementary File 3) with two fossil dates and a soft bound at three nodes: (1) the stem node of Rhododendron (56 Mya) (Collinson & Crane, 1978), (2) the crown node of Ericales (89.8 Mya) (Nixon & Crepet, 1993), (3) asterids-rosids (116-126 Mya) (Li et al., 2019) as constraints for calibrating the age of our tree". Please see lines 591-596 in the main text of the revised version (track changes).

References to the fossil data and the soft bound:

Collinson, M.E. & Crane, P.R. *Rhododendron* seeds from the Palaeocene of southern England. *Bot. J. Linn. Soc.* **76**, 195-205 (1978).

Nixon, K.C. & Crepet, W.L. Late Cretaceous fossil flowers of Ericalean affinity. *Am. J. Bot.* **80**, 616-623 (1993).

Li, H.T. *et al.* Origin of angiosperms and the puzzle of the Jurassic gap. *Nature Plants* **5**, 461-470 (2019).

1.7. Figure 1B: The Ks distribution in the upper right from the orthologs between Primula vulgaris and Vitis vinifera shows a different pattern than the species tree and the other Ks distribution. This should be clarified or discussed.

Response: Yes. The quality of genome assemble for Primula vulgaris is very low and has a poor fidelity (~87% genome representativeness). In the revised version of our manuscript, we have removed *Primula vulgaris* from the Ks analysis and have updated Figure 1 and all related content.

1.8. For the discussion section I would suggest the authors to discuss their findings about the WGDs and the findings in *Rhododendron williamsianum* [Soza et al., 2019]

Response: In the revised version of our manuscript, we have now referenced the studies of Wei et al. (2018) and Soza et al. (2019) on WGD in or at the ancestry of the Ericaceae, Actinidiaceae, and Theaceae.

Wei, C. *et al.* Draft genome sequence of *Camellia sinensis* var. *sinensis* provides insights into the evolution of the tea genome and tea quality. *Proc. Natl. Acad. Sci. U. S. A.* **115**, E4151-e4158 (2018).
Soza, V.L. *et al.* The *Rhododendron* genome and chromosomal organization provide insight into shared whole genome duplications across the heath family (Ericaceae). *Genome Biol. Evol.* **11**, 3357-71 (2019).

1.9. Line 46: Please break the large sentence into smaller sentences.
Response: Done. See revised version of our manuscript.

1.10. Figures 1+3: These figures are so packed that the text is unreadable. Please consider breaking the figure up into two figures, or moving some of the panels into the supplement.
Response: We agree with the reviewer and have reorganized our figures. The previous Fig. 1a was moved to Supplementary Fig. 9a., and Fig. 1b was enlarged. Previous Figs. 1 c, d, were moved to Supplementary Fig. 7, and Fig. 3a was moved to Supplementary Fig. 17.

1.11. Figure 3: What do the heatmaps to the left of the genes indicate? I assume it is gene expression, but valuable information does gene expression bring to the paper? What do the columns in the heatmap correspond to? Please discuss.
Response: Yes. The heatmap visualizes the gene expression of each enzyme coding gene. Here, different columns are corresponding to different flowering stages, from left to right, for T1-T5. We expanded the figure legend. We think the gene expression heatmap provides many informative values, such as a direct reference for easily checking the correlation (or contribution) of different gene members to a specific metabolic biosynthesis process.

1.12. Line 273: The authors reference Figure 3A+B when discussing the duplication percentages for the metabolic pathway. However, these figures do not contain duplication percentages.
Response: True, we did not provide duplication percentages in the Figure 3, but we did label the duplicates. The duplication percentages were now presented in the main text. Please see lines 315-330 (track changes), and Supplementary Table 15.

1.13. Figure 4B: Judging from the heatmap, it seems to me that L5 is a more mature stage than L6,7,8. Please consider revising the figure.
Response: We obtained the time-ordered gene coexpression network with a published method, TO-GCN (Chang et al., 2019). With time-series RNA sequencing data, the method constructs the gene coexpression network (GCN) and determines the time order of the nodes in a GCN. The reliability of the method was demonstrated with its successful prediction of experimentally validated regulatory cascades.

Chang, Y.M. *et al.* Comparative transcriptomics method to infer gene coexpression networks and its applications to maize and rice leaf transcriptomes. *Proc. Natl. Acad. Sci. U. S. A.* **116**, 3091-3099 (2019).

1.14. Figure 5: Since these genes are co-expressed, consider whether it is needed to show an expression heatmap for

Response: We have added expression heatmaps to Figure 5. Here, the expression heatmaps for individual structural genes in Fig. 5&6 may be helpful to investigate the involvement of individual genes in the subnetworks. And the expression heatmaps are also helpful to answer questions raised in under Comment #1.2.

##

Reviewer #2 (Remarks to the Author):

2.1. The upstream part of the carotenoid biosynthetic pathway is wrong. Carotenoids are synthesized from the MEP pathway. Although the authors labeled "MEP" pathway in their figures, but the pathway in figures and tables is the "MVA" pathway, which generally does not provide the substrate for carotenoid biosynthesis. Therefore, the analysis of carotenoid biosynthesis and regulation is entirely wrong.

Response: We agree with the reviewer and we indeed wrongly ascribed the genes from the “MVA” pathway to the “MEP” pathway. This has been corrected in the revised version of the manuscript. The corrected full annotation of the “MEP” pathway is presented in the Supplementary Fig. 17.

2.2. I doubt the annotation of UGTCs2 for crocin biosynthesis in this manuscript. I don't think Rhododendron synthesizes crocin. It is a question what these UGTCs2 members indeed do. They might take part in other metabolic pathways, but most probably not for crocin.

Response: Yes, we agree with the reviewer and have now removed all parts related to “croin” in the Results and Discussion.

2.3. The tandem arrangement of duplicated genes is not restricted to UGTGCs2, 4CL, and a few other enzymes as the authors identified in the manuscript. I looked up the annotation results in the supplemental files, and found such tandem duplication also exists for a large number of transcription factors. For example, there are 13 members of GRAS from 13G0079200 to 13G0080600, 10 of NAC from 11G0114800 to 11G0117000. There are also clusters of ERFs and MYBs. I wish the authors could provide their cloning and Sanger sequencing results to prove the existence of these clusters, instead of some mistakes in their assembly.

Response: Thanks. This is a really good comment which could indeed help strengthening the tandem duplication part by adding additional verification. In addition to traditional cloning and Sanger sequencing, long-read sequencing technologies, such PacBio and ONT, provide us with a very efficient way to verify the existence of tandem duplicated genes. Here, we mapped our PacBio long-reads back to the assembly, and examined whether the pair of adjacent tandem duplicated genes

or the intergenic region could be fully or partially recovered by the mapped long-reads. We indeed found that most (79–87%) of the duplicated genes could be recovered, fully or partially, both for TF or anthocyanin/flavonol biosynthetic genes or in the full genome level. These results provide very good evidence for the true existence of tandem gene clusters. Please see lines 261-268 and the figure (Supplementary Fig.15) in the revised manuscript (track changes).

Supplementary Fig. 15: Verification of tandem gene duplicates with long-reads mapping. **a**. a visualization of different types of verification of tandem gene duplicates. Blue line is representing a linear genomic region conveying a pair of gene duplicates. Red squares labeled with "TD1" and "TD2" are the genic regions of a pair of two tandem gene duplicates. Pink lines represent the long-reads mapped to the duplicated region; **b**. a summary table showing all pairs of tandem gene duplicates.

a

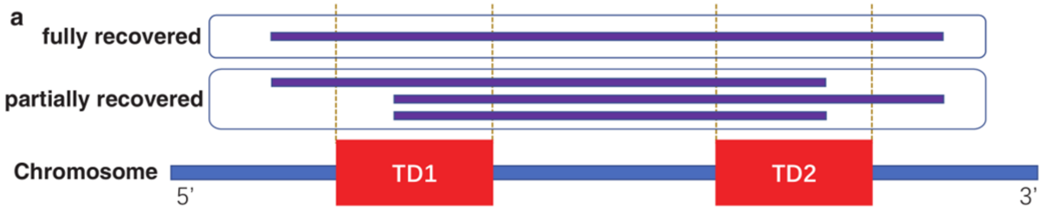

b

Whole genome	fully recovered	1830 (70.82%)
Whole genome	partially recovered	434 (16.80%)
TF	fully recovered	93 (75.61%)
TF	partially recovered	14 (11.38%)
Anthocyanin/flavonol	fully recovered	15 (53.57%)
Anthocyanin/flavonol	partially recovered	7 (25.00%)

##

Reviewer #3 (Remarks to the Author):

3.1. Lines 272-290: The findings on tandem duplication of flower coloration genes are interesting, but I have a few comments. Is the proportion of tandemly or proximally duplicated genes significantly different for flower related genes compared to the genome wide average? Typically 15-30% of genes in plant genomes are tandemly duplicated, so a formal statistical test will test if enriched duplication contributes to this trait.

It is also unclear if this proportion of TD flower coloration genes is different from other plant genomes.

Response: Thank you for this comment. For the revised version of our manuscript, we performed formal statistical tests and modified our text, accordingly. We now write: "Tandem duplicated (TD) genes were found enriched for GO terms related to secondary metabolic biosynthetic and modification processes. Furthermore, the proportion of TD/PD genes linked to flower coloration was significantly higher in *R. simsii* than that in related species (Supplementary Table 14)." in lines 311-315 (in the

revised manuscript, indicated by track changes).

3.2. Time-ordered expression dynamics of flower pigmentation are discussed in the next section, but it would be interesting to include expression analysis of TD/PDs at the single gene level in this section. TDs have high birth/death rates, with many having no expression at all, so it would be interesting to see if these duplicated genes are functioning during flower development.
Response: We have now added a description on gene expression of TD/PD duplicates. See revised manuscript, lines 332-334 (indicated by track changes). We agree that it will be important to clarify the mechanism governing gene expression differences among duplicates in further studies.

3.3. Line 386: The number of flowering related genes is higher in Rhododendron that Arabidopsis, but how does this compare with other species? I'm not sure if orthologs from other species are well-characterized, but this may be testable using the Orthogroup results.
Response: In the Arabidopsis flowering time database, FLOR-ID, a total of 295 (live update) protein-coding genes are categorized. All these genes are functionally verified and well-supported flowering time genes. In the present study, we identified flowering time genes in Azalea and other species by referring to the Orthogroups constructed by homology survey. If a gene was found in the same Orthogroup with a flowering time gene of Arabidopsis, then this gene was classified as a flowering time gene. Our sequence homology survey strategy may indeed present an overestimated number of flowering time genes. In fact, we detected 461 putative flowering related genes for Arabidopsis by referring to the FLOR-ID. Following this homology survey strategy, we found that the number of flowering related genes is not higher in Rhododendron than in other species. Thus, we have revised the result and deleted the sentence "…, a number that is considerably larger than the reported number for *Arabidopsis* (286 genes)", and have revised the discussion and deleted the sentence "Moreover, we predicted 424 flowering-time genes, considerably more than the reported number for Arabidopsis (286 genes), indicative of a greater complexity of the flower time regulatory network in Rhododendron".

3.4. Line 458: How do the individuals used for the expression analysis compare to the individual used for genome assembly? Are they the same cultivar?
Response: The individuals used for the expression analysis and those used for genome assembly are from the same wild population. We have added this information to the Materials and Methods of the revised version of our manuscript (track changes).

3.5. Line 488: FALCON-Phase was used for assembly, but the assembly of multiple haplotypes was not reported.
Response: The full genome assembly process was described in the Supplementary Note, and in lines 140-172 of this version there is a detailed description of how the nuclear genome was assembled step by step, as well as some related primary results.

The matrix of each assembly was presented in Supplementary Table 2.

3.6. Two draft assemblies were merged together, but these are not discussed. It would be useful to list the two programs that produced the 'best' assembly and the metrics of the unmerged and merged versions.
Response: This information is now available in the revised version of our manuscript (see previous comment).

3.7. Line 531 orthogroupsof to "orthogroups of"
Response: Corrected.

###
Reviewer #4 (Remarks to the Author):
4.1. First, the authors mentioned "The previous lack of a high-quality Rhododendron species whole-genome sequence", but did not give much detail of the quality of previous assemblies, so it is difficult to judge the reliability of the results of comparative analyses with other species. When a genome is not well assembled and annotated, it is difficult to know how many genes and how many copies of a gene are there in the genome.
Response: We have now compared our genome assembly with those previously reported for Rhododendron species in Table 1. We also summarized the main results in the Results section (lines 125-144, track changes).

4.2. Second, I did not see how redundancy was removed from the assembly and how the authors distinguished between gene duplication and redundancy. This is important. For example, how do you know the 425 genes related to flowering-time control were all or almost all real, so that the number is really considerably larger than that (280) in Arabidopsis?
Response: (1) On redundancy: We ran the assemblers, Canu, SMARTdenovo and WTDBG, to produce the haplotype-fused assembly under the default settings. SMARTdenovo and WTDBG were used to prepare the assembles v0.3 and v0.4, respectively. After evaluation, only these two primary assemblies were merged and then polished, and used for Hi-C scaffolding. After that, the assembly went through runs of gap-closing polishing. With chromatic contact data, redundancy was further removed by identifying the haplotigs (please see the following figure). To produce the final assembly, Redundans was used to further purge the redundancy of haplotigs.

The full genome assembly process was described in the Supplementary Note, and in lines 140-172 of the revised suppl. note one can now find a very detailed description of how the nuclear genome was assembled.

Response Figure Haplotigs (yellow square) identified chromatin contact data.

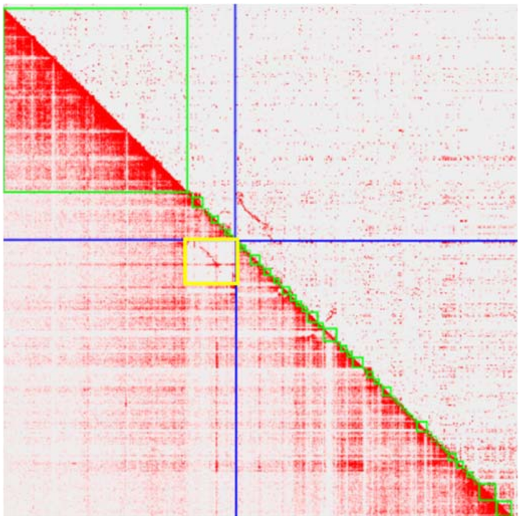

(2) Verification of duplicated genes with PacBio long-reads: Long-read sequencing technologies, such as PacBio and ONT, provide us with a very efficient way to verify the tandem duplicated genes. Here, we mapped our PacBio long-reads back to the assembly, and examined whether the pair of adjacent tandem duplicated genes or the intergenic region could be fully or partially recovered by the mapped long-reads. We found that 79-87% of those regions could be recovered, fully or partially, both for TF or anthocyanin/flavonol biosythetic genes or in the full genome level. These results provide very good verification for the existence of tandem gene clusters. Please see lines 265-272 of the revised version of our manuscript and the figure (Supplementary Fig.15).

(3) With respect to the number of flowering genes in Azalea, please see our response to one of the comments of reviewer 2.

4.3. Third, the authors identified 5 WGDs among the 5 lineages studied in Ericales (Fig. 1). That implies very frequent WGD events. I wonder how reliable the inferences were. In particular, a WGD occurred soon after the divergence between the Camellia genus and the common ancestor of the Actinidia and Rhododendron genera and a WDG occurred in the lineage. Why they were not due to a single WDG event?

Response: Ericales is a largely woody, economically important, order of ~12,000 species distributed in 21 or 22 families (APG IV), with an estimate of approximate 90 Mya for the minimum divergence. WGD is very common in angiosperms and here we did detect five WGD events in the order Ericales and Cornales. We believe that Ericaceae and Actinidiaceae share a WGD event, which is congruent with the results of Wei et al. (2018), while Soza et al. (2019) even suggested this WGD to have occurred in the common ancestor of Ericaceae, Actinidiaceae, and Theaceae. The incongruity might be partially due to different sampling or to the complex evolutionary history of Ericales. Further studies are necessary to clarify the problem. We emphasized this problem and discussed it in lines 422-425 (track changes).

Wei, C. *et al.* Draft genome sequence of *Camellia sinensis* var. *sinensis* provides insights into the evolution of the tea genome and tea quality. *Proc. Natl. Acad. Sci. U. S. A.* **115**, E4151-e4158 (2018).
Soza, V.L. *et al.* The *Rhododendron* genome and chromosomal organization provide insight into shared whole genome duplications across the heath family (Ericaceae). *Genome Biol. Evol.* **11**, 3357-71 (2019).

4.4. Fourth, the statement “we detected a 2:4 syntenic relationship between *R. simsii* and Actinidia chinensis29 (Fig. 1d), a 2:1 syntenic relationship between *R. simsii* and *Vitis vinifera*20, and a 1:4 syntenic relationship between *V. vinifera* and *V. corymbosum*, which provided additional proof for a shared WGD event among R. simsii, *V. corymbosum*, and *A. chinensis*” is not easy to understand. How about change it to, “we detected a 2:1 syntenic relationship between *R. simsii* and *Vitis vinifera*20, a 4:2 syntenic relationship between *Actinidia chinensis* and *R. simsii*29, and a 4:1 syntenic relationship between *V. corymbosum* and *V. vinifera*, which provided additional proof for a shared WGD event among *R. simsii*, *V. corymbosum*, and *A. chinensis*” ?
Response: We changed the expression accordingly. Please see lines 182-187.

4.5. Fifth, in Fig. 1 and throughout the paper, there are many cases where *K*s is larger than 2. One should be aware that a *K*s > 2 is difficult to estimate reliably. And so are the ratios *K*a/*K*s.
Response: Yes, we agree with the reviewer. We now focused our study only on cases of *K*s less than 2. Please see Figs 1, 2a and Supplementary Figures 12 and 14 in the revised version of our manuscript.

4.6. Sixth, Lines 218-219: Please define “proximal”, “transposed” and “dispersed” duplicates.
Response: Done. We provided short descriptions on how these types of duplicates were identified in M&M. Please see lines 602-606 in the main text with tracks.

4.7. Seventh, Line 308 and throughout the paper, you used “regulatory interactions” or “regulatory network”. To be qualified for these terms, you must have identified the TF binding sites on the promoters of the putative target genes. It seems to me you have not done that and if this is case, you should write “co-expression network” rather than “regulatory network”.
Response: We have corrected this by replacing “regulatory” with “co-expression” throughout the revised manuscript, so as to avoid potential overinterpretation.

4.8. Eighth, Lines 489-491: Which assemblers gave the two optimal assemblies v0.3 and v0.4?
Response: The full genome assembly process was described in the Supplementary Note, and in lines 140-172 of the revised version of the suppl. note, one can find a detailed description of how the nuclear genome was assembled into different versions

step by step. The matrix of each assemblies was presented in Supplementary Table 2.

4.9. Ninth, Line 496: How did you decide “five rounds of Pillon polishing”?
Response: Pilon was employed to improve the genome assembly based on Illumina reads aligned to the assembly. After each Pilon run, we calculated the inconsistencies (single base differences and structural differences) between the assembly and the evidence in the reads. We found a significant number of differences for 1-2 runs, while few for runs after the 3rd one. So we chose these 5 runs of Pilon polishing.

4.10. Tenth, in MAKER2 gene predictions, which method(s) was used for ab initio predictions?
Response: For *ab initio* gene prediction with MAKER2, we used AUGUSTUS (version 3.3). The full gene structural and functional annotation process was described in lines 280-324 of the Supplementary Note.

REVIEWERS' COMMENTS:

Reviewer #1 (Remarks to the Author):

Thank you for addressing my comments.

Reviewer #2 (Remarks to the Author):

This is a revision of the previously submitted manuscript describing the chromosome level genome of Rhododendron simsii. In this revision, the authors have replied to most of the questions and comments reasonably, and have made their improments accordingly. The manuscript is clearly written, and is now in a good shape.

I have a few comments for the authors.
1. The genome size estimated by K-mer (~500Mb) is quite different from the flow cytometry results (414.63Mb). Please provide a brief explanation.

2. Uncharacterized TEs occupy 19.24% of the genome, which is a relatively high ratio. I wish the authors could check their annotation and provide a reasonable explanation or postulation.

3. The authors made corrections to Supplementary Figure 17. However, there are still some mistakes. First, initial substrates for the MEP pathway are GAP and pyruvate, not GAP alone. Second, GPPS and FPPS are not involved in carotenoid biosynthesis. FPP is not related to the MEP pathway, and GPPS cannot be distinguished from GGPPS (and it seems most angiosperms don't have real GPPS). While Rhododendron simsii does not prode lactucaxanthin, this branch can be removed from the figure. Moreover, the heat maps for both CRTZ and BCH should be moved to the xanthophylls block.

Reviewer #3 (Remarks to the Author):

The authors have addressed my previous concerns

Reviewer #4 (Remarks to the Author):

The authors have satisfactorily responded to my comments. I have only two minor comments on the revised MS:

1. In lines 124-126, the authors wrote "the BUSCO value is much better for Rhododendron simsii than the assemblies for the two other species. However, their score of 93.68% is not much better than that (92.80%) for R. delavayi.

2. Line 541: Should TSD be TRD?

Reviewers' comments and our responses:
###
Reviewer #1:
No new comments were provided and the reviewer appreciated our response to his/her original comments.

###
Reviewer #2:
This is a revision of the previously submitted manuscript describing the chromosome level genome of *Rhododendron simsii*. In this revision, the authors have replied to most of the questions and comments reasonably, and have made their improvements accordingly. The manuscript is clearly written, and is now in a good shape.
I have a few comments for the authors.
1. The genome size estimated by *K*-mer (~500Mb) is quite different from the flow cytometry results (414.63Mb). Please provide a brief explanation.
Response: The high level of heterozygosity (1.78% from *K*-mer estimation) and the high rate of duplications (55.09% from *K*-mer estimation) of this azalea genome are expected to inflate the estimated *K* -mer and the assembled genome sizes. At the same time, dye type, dyeing time, cell/nuclei status and many other parameters might affect the flow cytometry results. Without further investigation, it is still difficult to discern the reason(s) for the observed discrepancy between the reported estimations. Furthermore, we have corrected the genome size from *K*-mer estimation to ~525 Mb.
2. Uncharacterized TEs occupy 19.24% of the genome, which is a relatively high ratio. I wish the authors could check their annotation and provide a reasonable explanation or postulation.
Response: We did double-check our entire computational pipeline and summary statistics of the repeat annotation and the results confirm our earlier reported values. We added the following sentence: "The uncharacterized (unknown) TEs may contain highly degenerated TE copies or lack distinct protein-coding sequences required for further classification. More work is needed to elucidate the structural and/or sequence diversity of the uncharacterized TEs." (see lines 187-190).
3. The authors made corrections to Supplementary Figure 17. However, there are still some mistakes. First, initial substrates for the MEP pathway are GAP and pyruvate, not GAP alone. Second, GPPS and FPPS are not involved in carotenoid biosynthesis. FPP is not related to the MEP pathway, and GPPS cannot be distinguished from GGPPS (and it seems most angiosperms don't have real GPPS). While *Rhododendron simsii* does not prode lactucaxanthin, this branch can be removed from the figure. Moreover, the heat maps for both CRTZ and BCH should be moved to the xanthophylls block.
Response: Yes, thank you for pointing this out. We agree with the reviewer and have corrected the Supplementary Figure 17. We added the initial substrate pyruvate to the pathway and removed FPP, FPPS, GPPS, and the branch leading to lactucaxanthin from the pathway. The heat maps have been adjusted accordingly to reflect this. Moreover, we changed the pathway to better reflect that isopentenyl diphosphate and dimethylallyl diphosphate are joined together by GGPPS to give geranylgeranyl diphosphate.

###
Reviewer #3:
No new comments were provided and the reviewer acknowledged our response to his/her original comments.
###
Reviewer #4:
The authors have satisfactorily responded to my comments. I have only two minor comments on the revised MS:
1. In lines 124-126, the authors wrote "the BUSCO value is much better for *Rhododendron simsii* than the assemblies for the two other species. However, their score of 93.68% is not much better than that (92.80%) for *R. delavayi*.
Response: Yes, we improved the expression by removing "much" (see line 128).
2. Line 541: Should TSD be TRD?
Response: Corrected.
